# Faunal persistence and ecological flexibility in Pleistocene Southeast Asia revealed through multi-isotope analysis

**DOI:** 10.1126/sciadv.adu3642

**Published:** 2025-10-15

**Authors:** Nicolas Bourgon, Tina Lüdecke, Jennifer N. Leichliter, Chris Baumann, Sven Brömme, Marissa Vink, Anne-Marie Bacon, Jeremy McCormack, Thi Mai Huong Nguyen, Anh Tuan Nguyen, Pierre-Olivier Antoine, Jean-Luc Ponche, Philippe Duringer, Fabrice Demeter, Souliphane Boualaphane, Thonglith Luangkhoth, Elise Dufour, Jean-Jacques Hublin, Klervia Jaouen, Thomas Tütken, Alfredo Martinez-Garcia, Patrick Roberts

**Affiliations:** ^1^ Department of Coevolution of Land Use and Urbanisation, Max Planck Institute of Geoanthropology, 07745 Jena, Germany.; ^2^ Department of Human Evolution, Max Planck Institute for Evolutionary Anthropology, 04103 Leipzig, Germany.; ^3^ Emmy Noether Group for Hominin Meat Consumption, Max Planck Institute for Chemistry, 55128 Mainz, Germany.; ^4^ Senckenberg Centre for Human Evolution and Palaeoenvironment at the University of Tübingen (SHEP Tübingen), AG Biogeology, 72074 Tübingen, Germany.; ^5^ Université Paris Cité, CNRS, BABEL, F-75012 Paris, France.; ^6^ Department of Geosciences, Goethe University Frankfurt, 60438 Frankfurt, Germany.; ^7^ Anthropological and Palaeoenvironmental Department, Institute of Archaeology, Hoan Kiem District, Ha Noi, Vietnam.; ^8^ Institut des Sciences de l’Évolution de Montpellier, UMR 5554, Université de Montpellier, CNRS, IRD, 34095 Montpellier Cedex 5, France.; ^9^ Laboratoire Image, Ville Environnement, UMR 7362, UdS CNRS, Université de Strasbourg, 67000 Strasbourg, France.; ^10^ Ecole et Observatoire des Sciences de La Terre, Institut de Physique du Globe de Strasbourg (IPGS), UMR 7516, CRNS, Université de Strasbourg, 67000 Strasbourg, France.; ^11^ Lundbeck Foundation GeoGenetics Centre, Globe Institute, University of Copenhagen, 1165 Copenhagen, Denmark.; ^12^ Eco-anthropologie (EA), Anthropologie biologique et Bio-archéologie, Muséum National d’Histoire Naturelle, CNRS, Université Paris Cité, Musée de L’Homme, 75016 Paris, France.; ^13^ Department of Heritage, Ministry of Information, Culture and Tourism, Vientiane, Lao Democratic People’s Republic.; ^14^ BioArch -BioArchéologie, Interactions Sociétés Environnements, UMR 7209, Muséum National d'Histoire Naturelle, CNRS, 75005 Paris, France.; ^15^ Chaire de Paléoanthropologie, CIRB, Collège de France, Université PSL, CNRS, 75005 Paris, France.; ^16^ Géosciences Environnement Toulouse, Observatoire Midi Pyrénées, 31400 Toulouse, France.; ^17^ Institute of Geosciences, Johannes Gutenberg University, 55128 Mainz, Germany.; ^18^ Climate Geochemistry Department, Max Planck Institute for Chemistry, 55128 Mainz, Germany.

## Abstract

Southeast Asia boasts some of the world’s most diverse ecosystems, yet it has experienced a dramatic decline in biodiversity due to extensive deforestation in recent decades. Understanding how species adapted to past habitat loss could provide valuable insights for developing effective conservation strategies to address current threats. We apply state-of-the-art isotope measurements of enamel-bound zinc (δ^66^Zn) and nitrogen (δ^15^N_enamel_) analyses to fossil mammal teeth from Coc Muoi [148 to 117 thousand years (ka) and Duoi U’Oi (70 to 60 ka)], Vietnam. Alongside published regional data (δ^66^Zn, δ^13^C, and δ^18^O), we explore how different taxa adapt their diets and behaviors in the face of climatic and environmental changes. We show that foraging in diverse habitats and diversifying food consumed is associated with extant species, while extinct or locally extirpated taxa show these traits to a lesser extent. We underscore the precariousness of certain species and the pressing need for robust conservation policies to safeguard Southeast Asia’s biodiversity.

## Teaser

Multi-isotope approaches provide valuable insights into mammalian ecological niches and factors contributing to extirpation.

## INTRODUCTION

Southeast Asia is home to some of the most species-rich ecosystems on Earth (*1*). However, its growing urban population (*2*), large-scale agricultural expansion (*3*), and infrastructural development (*4*) have caused severe loss, degradation, and fragmentation of its forests over the past decades (*5*), leading to a sharp biodiversity decline (*6*). A thorough understanding of how species adapted or failed to adapt to habitat loss (or change) in the past could prove valuable to designing effective conservation plans in the face of these contemporary threats. Mainland Southeast Asia witnessed a series of faunal extinctions over the course of the Pleistocene epoch, including, among the latest recorded taxa, that of the largest known primate ever to inhabit the Earth, *Gigantopithecus blacki*, the proboscidean *Stegodon orientalis*, and the giant tapir *Megatapirus augustus* (*7*). Similarly, local extirpations (i.e., taxa termination in a given geographic area) are recorded for a number of other taxa, many of which are listed as endangered species today, such as orangutan (*Pongo* sp.), rhinoceros (*Rhinoceros sondaicus*, *Rhinoceros unicornis*, and *Dicerorhinus sumatrensis*), the giant panda (*Ailuropoda melanoleuca*), or Malayan tapirs (*Tapirus indicus*) (*8*). Discussion of the factors behind these extinctions and extirpations often focuses on the stark climatic and environmental changes documented throughout the Pleistocene, oscillating between humid and drier periods at a high and irregular pace (*7*). However, taxa responses to climate change are complex and dependent on a variety of factors, including their life histories, their ability to adapt their diets and behaviors, and their interaction with other species (including humans), making detailed analyses of their niches across space and time essential.

Stable isotope analyses of carbon, oxygen, and nitrogen from animal tissues have proven themselves key for understanding the life history and dietary and habitat preferences of extinct and extant taxa (*7*, *9*, *10*) and, thus, represent ideal methods to directly explore the individualistic response of animals to climatic and environmental changes. For instance, δ^13^C values are especially powerful proxies in tropical environments because of the ecological distinction that emerged in the Late Neogene (*11*) between C_3_ and C_4_ plants, respectively, primarily associated with closed forested and open grassland environments in the tropics (*12*). The δ^13^C variability, alongside that seen in δ^18^O values, has also been used to explore niche partitioning along a vertical gradient in dense canopy forests (*13*). The δ^18^O values also allow us a glimpse into past climatic conditions, broadly relating to temperature and humidity, and, for herbivores, diet, as different plant parts will exhibit distinct values given changing evaporative potentials (*14*). Meanwhile, δ^15^N values can be used to tease apart different trophic levels (*15*). Differences in δ^15^N values of a food web’s producers (i.e., plants) are governed mainly by a balance between atmospheric fixation, denitrification, and ammonia volatilization within the soil (*16*). Local baselines can also be affected by various physical and chemical variables such as mean annual rainfall, soil maturity, and soil pH (*17*). Using bulk δ^15^N values as an indicator of trophic levels assumes a consistent and predictable difference in nitrogen isotopes between the diet and the examined tissue of a consumer (typically 2 to 6‰) (*18*–*21*), and, by analyzing sympatric animals as a frame of reference for each trophic level, trophic relations between specimens, species, or populations can be assessed. These proxies have been successfully applied to investigate the canopy feeding niches in extant arboreal primates (*22*) or to better understand the dietary and habitat preferences of extinct fossil taxa such as the giant ape *Gigantopithecus blacki* (*23*). However, assessing dietary and habitat preferences of extinct taxa can become especially challenging across space and time since shifts in isotopic values could equally result from various factors, such as changes in the diet, changes in the climatic and environmental conditions, or changes in the isotopic local baseline (*17*). Moreover, dentine or bone collagen, which is commonly analyzed for δ^13^C and δ^15^N, is often not preserved in tropical settings or in fossils older than 20 to 100 ka because of the rapid postmortem loss of organic matter.

An effective way to solve this issue is by using other independent proxies. In recent years, methods for reconstructing diet and trophic ecology in contexts where collagen is not preserved have emerged, such as the oxidation-denitrification method, allowing for tooth enamel nitrogen isotope analyses (*18*–*20*) or using zinc isotope ratios (*24*–*31*). Specifically, tooth enamel is the hardest tissue formed in animals and the least susceptible substance to postmortem diagenetic alteration due to its low porosity, low organic matter content, and large and compact bioapatite crystallites [see Wang and Cerling (*32*) and Kohn *et al.* (*33*) and references therein]. Enamel-bound organic δ^15^N analysis (denoted herein as δ^15^N_enamel_ to emphasize the novelty of using enamel-bound nitrogen compared to traditionally used collagen-bound) offers the possibility of tracking traditional trophic-level changes seen in δ^15^N over greater timescales, given the longevity of this intracrystalline–bound organic material over dentine or bone collagen (*34*, *35*). Meanwhile, δ^66^Zn analysis has emerged as an independent trophic proxy, notably thought to be able to tease apart omnivores from carnivores and herbivores (*27*, *28*). Variability in zinc stable isotopes in terrestrial food webs mostly reflects biological fractionation and natural variations in soils and plants. The δ^66^Zn values of soils vary according to factors such as the lithology of the underlying bedrock, the origin of organic matter, and the biogeochemical cycling of zinc [see Opfergelt *et al.* (*36*) and references therein]. Plants then obtain zinc from the soil, with fractionation occurring as heavier Zn isotopes are preferentially extracted out of the xylem [see Wiggenhauser *et al.* (*37*) and references therein]. This leads to isotopic distinctions between different parts of the same plant and among various plant species, whereby leaves generally exhibit lower δ^66^Zn values than stems, and higher-growing species, such as shrubs or trees, tend to have lower δ^66^Zn values compared to lower-growing ones like grasses or herbaceous plants. Zinc isotope composition in animals is then primarily controlled through the diet (*38*, *39*) and undergoes somewhat predictable mass-dependent fractionation within the body and along food chains (*38*–*43*). Muscles exhibit low values, and, as the primary consumed tissues of prey, successively lower δ^66^Zn values can be observed as trophic levels increase [0.45 to 0.60‰, which roughly corresponds to the offset between muscle and diet (*38*, *39*)]. Previous projects already used a combined δ^66^Zn and δ^15^N stable isotope approach on bone bioapatite and bone collagen, respectively, with great success (*29*), and highlighted the benefits of combining these two complementary methods by obtaining more detailed and refined dietary reconstruction.

**Fig. 1.**
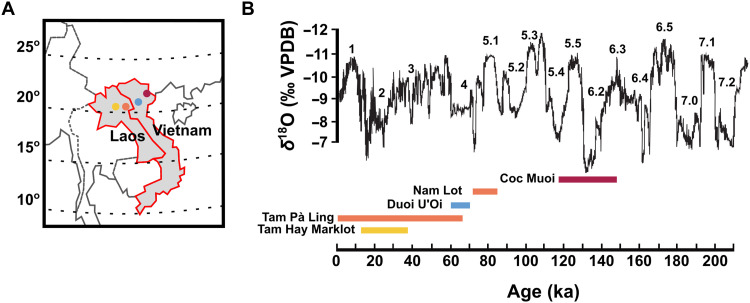
Location of sites in northern Laos (Tam Pà Ling, Nam Lot, and Tam Hay Marklot) and northern Vietnam (Coc Muoi and Duoi U’Oi), and the site ages versus δ^18^O speleothem records from Sanbao, Dongee, and Hulu Chinese caves. The fossil sites analyzed in this study are situated in northern Vietnam (Coc Muoi and Duoi U’Oi) and northeastern Laos (Tam Pà Ling, Nam Lot, and Tam Hay Marklot), all lying within the latitudinal range of 20° to 23°N (**A**).The curve of δ^18^O values [‰ Vienna Peedee belemnite (VPDB)] is modified from Wang *et al.* (*65*), whereby the fluctuations broadly correspond to changes in precipitation (i.e., a decrease in δ^18^O values corresponds to increasing amounts of precipitation) (**B**). The age intervals of each site have been placed in relation to the oscillation in the curve of δ^18^O records and marine isotope stages and substages (indicated by the numbers above the curve), from the oldest (right) to the youngest (left). Tam Pà Ling and Nam Lot are both located on the Pà Hang Mountain and thus share the same marker and color on the map. The color of each site on the map is the same as the one in the age intervals.

Here, we conduct a systematic comparison of multi-isotopic data from sites where δ^66^Zn, δ^15^N_enamel_, δ^13^C, and δ^18^O (the latter two from the carbonate fraction) values of tooth enamel from a wide range of large mammals are available (Fig. 1A). We report δ^15^N_enamel_, using the oxidation-denitrification method (*18*), and δ^66^Zn data of 141 specimens from two mammalian assemblages from northern Vietnam (Fig. 1 and table S1), namely the Coc Muoi site [148 to 117 ka (*44*); *n* = 84] covering in large part MIS 6 and the MIS-4 site of Duoi U’Oi [70 to 60 ka (*45*), *n* = 60], for which δ^13^C and δ^18^O values measured on the same aliquot are already available (table S2) (*10*). This study also makes use of already published δ^66^Zn, δ^13^C, and δ^18^O data from northern Laos (Fig. 1) from the MIS 3–2 site of Tam Hay Marklot [38.4 to 13.5 ka (*27*)] and the Pà Hang Mountain [composed of data from both the MIS 5 Nam Lot 86 to 72 ka (*28*, *46*)] sites, and those from the long stratigraphic sequence at Tam Pà Ling [70 to 1.1 ka (*28*)], as well as the only available δ^15^N_enamel_ values of Southeast Asia from Tam Hay Marklot (tables S3 and S4) (*19*). All assemblages are from karst cave infills and were subjected to similar taphonomic processes typical of karstic systems [see original publications and reference therein for a full description of sites and paleontological contents (*27*, *44*, *46*–*48*)].

Each of these five fossil assemblages is extensive in terms of taxonomic and specimen numbers, contains almost identical taxa, and, when combined, covers, albeit discontinuously, the last approximately 159 ka, a period that witnessed oscillations between stadials (cold phases) and interstadials (warmer climate phases) and key highly contrasted climatic events (including the Last Interglacial and the Last Glacial Maximum) (Fig. 1B). All sites are located in a narrow latitudinal belt running through the northern regions of Laos and Vietnam, between 23° and 20° (Fig. 1A), which minimizes environmental variations from the cline effect (temperature, distance from the coast, rainfall seasonality, and amount of rainfall) and allows for a more rigorous comparison between sites. The main abiotic discrepancy is elevation, ranging from lowland alluvial plain (Duoi U’Oi) to medium mountain sites (~1120 m at Pà Hang Mountain). All sites comprise a varying degree of mosaic environments with climatic conditions encapsulated by each site’s age, inducing varying proportions of the closed forest canopy, intermediate rainforest/woodland, and open environments of tropical wet and humid subtropical climates [see Bacon *et al.* (*10*) for a thorough environmental comparison between sites]. These faunal assemblages can thus provide valuable information on the range of adaptive capacities of taxa in response to changing climates across space and time (especially precipitation amounts and seasonality) in a tropical rainforest setting. In addition to grouping taxa by dietary categories (carnivorous, omnivorous, and herbivorous), we also divide taxa into extant, extirpated, and extinct categories based on their modern counterpart status in the studied region (i.e., Laos and Vietnam) to investigate their ecological response in relation to their International Union for Conservation of Nature (IUCN) regional conservation assessments.

## RESULTS

**Fig. 2.**
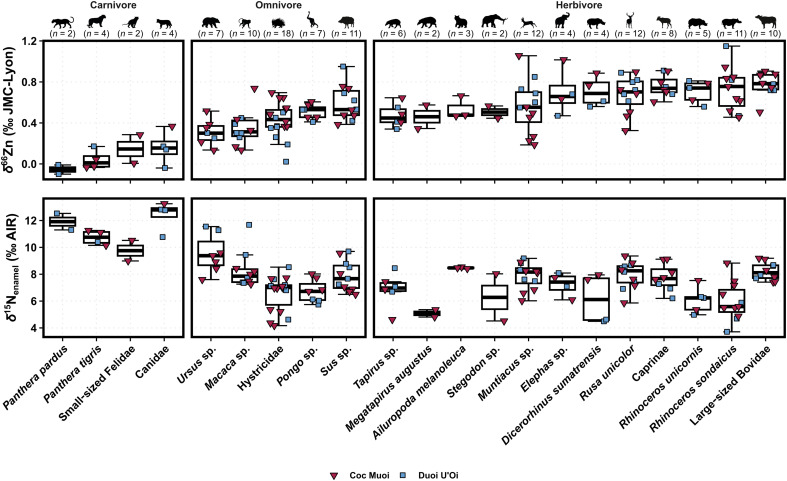
Box and whisker plots of δ^66^Zn values (‰ JMC-Lyon) and δ^15^N_enamel_ (‰ AIR) in tooth enamel for each taxon from Coc Muoi and Duoi U’Oi. Each of the colors and symbols represents specimens from different sites: Coc Muoi (148 to 117 ka; upside-down burgundy triangles) and Duoi U’Oi (70 to 60 ka; blue squares). The δ^66^Zn values of Coc Muoi are normalized to the average δ^66^Zn value of all other Southeast Asian sites (i.e., Tam Hay Marklot, Pà Hang Mountain, and Duoi U’Oi; see text for details). Overall, carnivores exhibit the lowest δ^66^Zn values (δ^66^Zn = 0.08 ± 0.14‰ 1σ, *n* = 12) and herbivores the highest values (δ^66^Zn = 0.66 ± 0.19‰ 1σ, *n* = 79), while δ^66^Zn values from omnivores are intermediate (δ^66^Zn = 0.44 ± 0.18‰ 1σ, *n* = 53). Herbivores and omnivores have the lowest δ^15^N_enamel_ values [δ^15^N_enamel_ = 7.3 ± 1.4‰ 1σ (*n* = 79) and δ^15^N_enamel_ = 7.6 ± 1.6‰ 1σ (*n* = 53), respectively], and carnivore values are the highest [δ^15^N_enamel_ = 11.3 ± 1.3‰ 1σ (*n* = 12)]. The boxes represent the 25th to 75th percentiles, with the median represented by a bold horizontal line. The taxa follow an ascending order per dietary group based on their δ^66^Zn values. The average δ^66^Zn and δ^15^N_enamel_ analytical repeatability of samples was 0.02 and 0.76‰, respectively.

All measured δ^66^Zn and δ^15^N_enamel_ values from Coc Muoi and Duoi U’Oi can be found in tables S5 and S6, and all values from the reference materials are reported in tables S7 and S8. The absence of a mixing line between Zn concentration and δ^66^Zn values (fig. S1) suggests no significant postmortem Zn uptake. An intersite comparison shows Coc Muoi’s δ^66^Zn values to be higher than in all other currently analyzed Southeast Asian sites by roughly 0.25‰ (fig. S2). Similar taxa, number of specimens, and especially consistent trophic spacings (fig. S2) across sites nonetheless allowed for Coc Muoi’s higher δ^66^Zn values to be easily normalized to the global mean of the other sites (Fig. 2 and fig. S2) to allow for direct comparisons between taxa across all sites (raw and corrected values are shown in fig. S3). Specifically, the overall mean δ^66^Zn value was calculated for all sites except Coc Muoi, the mean of which was determined separately. The difference between these two means was computed to obtain a correction factor, which was subsequently subtracted from the δ^66^Zn values of Coc Muoi, effectively adjusting its values to align with the baseline of the other sites. The normalized δ^66^Zn values (*n* = 144) obtained from tooth enamel range from −0.10 to 1.15‰ (Fig. 2 and fig. S2).

The relationship between N content and δ^15^N values was investigated and suggested that no postmortem uptake had occurred (fig. S4). The δ^15^N_enamel_ values from Coc Muoi and Duoi U’Oi (*n* = 144) range from 3.7 to 13.3‰ (Fig. 2). The values observed across plant parts and canopy height in tropical rainforests can vary by up to multiple typical trophic level spacing (*16*, *49*, *50*) and likely explains the large range of values given the diversity of different plant parts and canopy heights favored across taxa in the current study. The trophic spacing between carnivores and omnivores or herbivores is roughly +4.0‰, which is centered within the observed range of +2 to 6‰ per trophic level for δ^15^N identified in most tissues (*18*–*21*), while values from omnivores and herbivores are similar.

**Fig. 3.**
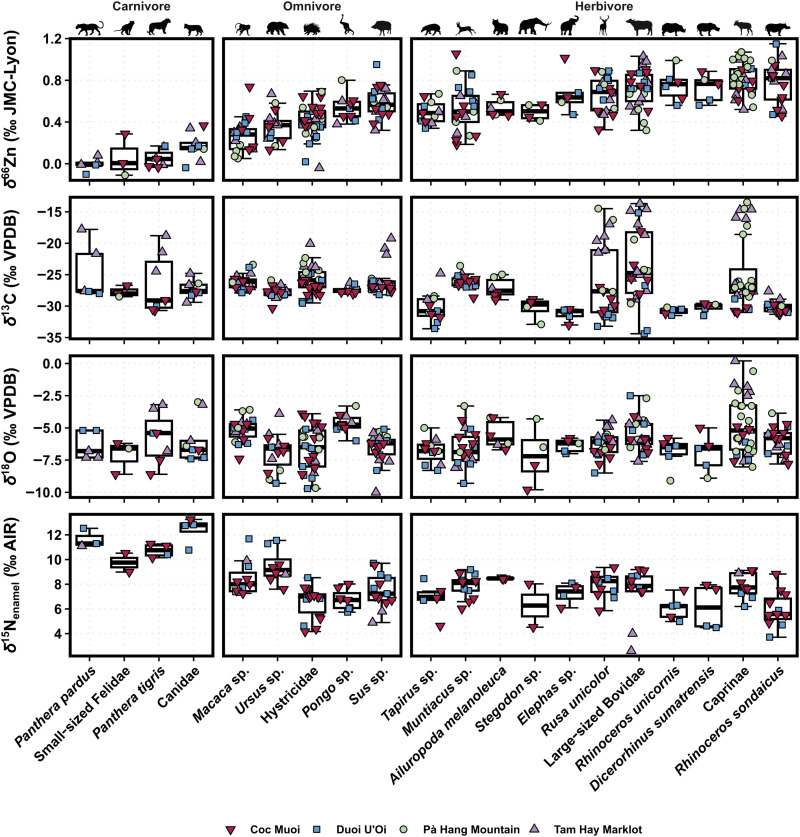
Box and whisker plots of δ^66^Zn values (‰ JMC-Lyon), δ^13^C (‰ VPDB), δ^18^O (‰ VPDB), and δ^15^N_enamel_ (‰ AIR) in tooth enamel for each taxon. Each of the colors and symbols represents specimens from different sites: upside-down burgundy triangles for Coc Muoi (148 to 117 ka), blue squares for Duoi U’Oi (70 to 60 ka), green circle for Pà Hang Mountain (which include Tam Pà Ling and Nam Lot, respectively 70 to 1.1 and 86 to 72 ka), and purple triangle for Tam Hay Marklot (38.4 to 13.5 ka). The δ^13^C values have been converted to those of the food web’s primary carbon sources for better comparability between species (see text S1), and both δ^13^C and δ^18^O values are taken from Bacon *et al.* (*10*, *46*) and Bourgon *et al.* (*27*, *28*). The δ^66^Zn values from Tam Hay Marklot are taken from Bourgon *et al.* (*27*), and those from Nam Lot and Tam Pà Ling from Bourgon *et al.* (*28*). The δ^15^N_enamel_ values from Tam Hay Marklot are taken from Leichliter *et al.* (*19*). The δ^66^Zn values of Coc Muoi are normalized to the average δ^66^Zn value of all other Southeast Asian sites (i.e., Tam Hay Marklot, Pà Hang Mountain, and Duoi U’Oi; see main text for details). The boxes represent the 25th to 75th percentiles, with the median represented by a bold horizontal line. The taxa follow an ascending order per dietary group based on their δ^66^Zn values.

While later-forming teeth were favored and targeted to reconstruct adult diet and thus avoid in utero, breastfeeding, and/or weaning signals, which could otherwise obscure other dietary signals, this was not possible in every case because of limited specimen availability. However, neither δ^66^Zn nor δ^15^N_enamel_ values correlate with tooth position and formation sequence (figs. S5 and S6), indicating sampled enamel mineralization postweaning, except, in both cases, with a single taxon for each proxy: the large-sized bovids showing a positive relationship between δ^66^Zn values and formation sequence, and the caprines showing a negative relation between δ^15^N_enamel_ values and formation sequence. This most likely results from sampling bias rather than an in utero or breastfeeding signal being recorded as it should otherwise be observed in both trophic level proxies for both taxa. Moreover, the full variability of later-forming teeth typically overlaps with that of early-forming teeth, suggesting that all δ^66^Zn and δ^15^N_enamel_ values can be used equally. Our compiled multi-isotope dataset (Fig. 3), which incorporates these latest δ^66^Zn and δ^15^N_enamel_ values as well as δ^66^Zn, δ^13^C, and δ^18^O from northeastern Laos (Tam Hay Marklot, Nam Lot, and Tam Pà Ling) (*27*, *28*, *46*), δ^13^C and δ^18^O from Coc Muoi and Duoi U’Oi (*10*), and a few δ^15^N_enamel_ values from Tam Hay Marklot (*19*), can be found in tables S2, S4, S5, and S6.

## DISCUSSION

**Fig. 4.**
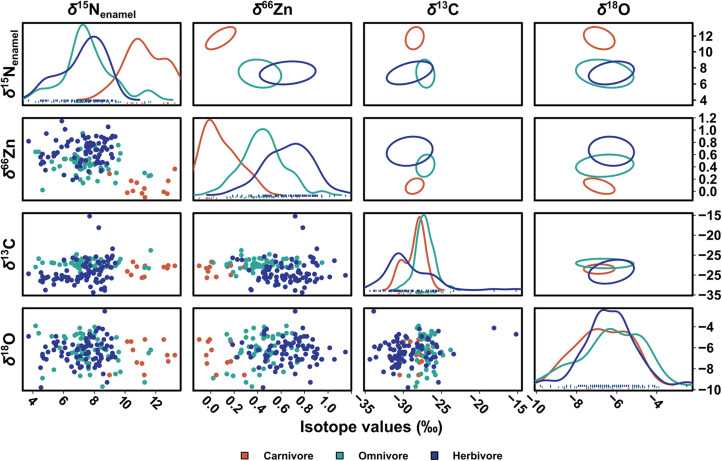
Scatterplots, density distribution, and 40% predictive ellipses of δ^15^N_enamel_ (‰ AIR), δ^66^Zn (‰ JMC-Lyon), δ^13^C (‰ VPDB), and δ^18^O (‰ VPDB) of dietary groups from Coc Muoi and Duoi U’Oi. The predictive ellipses (i.e., a region for predicting a new observation in the population) approximate a region that contains randomly selected 40% of the population of each dietary group. Hence, an inherent variability in how the ellipses are drawn exists, but all exhibit the same overall characteristics (fig. S8). When dietary groups (i.e., carnivorous taxa in red, omnivorous in teal, and herbivorous in blue) are compared, δ^15^N_enamel_ values cluster into high (composed primarily of carnivores) and low values (composed of herbivores and omnivores), while δ^66^Zn values separate into low (carnivores), intermediate (omnivores), and high (herbivores). The δ^66^Zn and δ^15^N_enamel_ values broadly offer comparable interpretations for carnivores’ and herbivores’ diets in these tropical rainforest environments and show similar relationships with δ^13^C and δ^18^O values. The δ^66^Zn values of Coc Muoi are normalized to the average δ^66^Zn value of all other Southeast Asian sites (i.e., Tam Hay Marklot, Pà Hang Mountain, and Duoi U’Oi). Note that the δ^13^C values are converted to those of the food web’s primary carbon sources (see text S1), and both δ^13^C and δ^18^O values are taken from Bacon *et al.* (*10*).

Our data confirm the utility of recent isotope approaches for teasing apart trophic partitioning in contexts where collagen is not preserved. Omnivores’ δ^66^Zn values clearly display an intermediate distribution between herbivores and carnivores, as opposed to δ^15^N_enamel_ values, which show a largely single-peak distribution overlapping with herbivores (Fig. 4). The considerable range of δ^66^Zn values observed so far in omnivorous taxa (*27*, *28*) reinforces that it is at least partially explained by the relative inclusion of animal matter in the diet. Omnivores’ δ^66^Zn and δ^15^N_enamel_ values are not significantly related [adjusted coefficient of determination (*R*^2^) = 0.02, *F*(1, 55) = 2.25, *P* = 0.14], except if wild boars (*Sus* sp.) are excluded [adjusted *R*^2^ = 0.11, *F*(1, 42) = 6.57, *P* = 0.01]. This could be related to the inclusion of plant underground organs in their diet (*51*), whose δ^66^Zn and δ^15^N values are both typically higher than other plant organs (*52*, *53*). As omnivore taxa are opportunists that can consume a large variety of resources, combining δ^66^Zn and δ^15^N_enamel_ values can offer a more nuanced dietary reconstruction than possible with any one proxy alone. Combining these methods presents undeniable benefits and, as is increasingly acknowledged for most isotope systems, profits from a combined use, particularly in deep-time contexts where such detailed, direct trophic insights have previously been lacking.

While varying δ^66^Zn baselines have been observed between ecosystems (*24*, *26*–*28*, *31*, *54*), the values obtained from Coc Muoi confirm baseline effects operating on regional scales and even within a particular biome. Previous suspicions of bedrock-induced δ^66^Zn variability (*27*, *28*, *55*) align with the fact that Duoi U’Oi and all three Laos sites sit on the Annamitic Mountain Range, while Coc Muoi is found on the Ha Lang domains. Notably, its higher δ^66^Zn values and the carbonate-dominated Ha Lang domain (*56*) agree with expectations for such geology (*57*). The solid agreement between trophic spacings (Kruskal-Wallis between diet pairs across sites: χ^2^ = 11, df = 11, *P* = 0.44; fig. S2) and the almost identical faunal assemblages across sites allowed for simple data normalization in this case. While δ^15^N_enamel_ values at Coc Muoi and Duoi U’Oi do not exhibit baseline differences, local δ^15^N baselines can still vary because of multiple environmental influences (*17*). This also highlights the importance and power of a multi-isotope approach to studying past animal niches, as baselines can vary differently from one isotope system to another, regardless of environment and climate.

With these insights into the operation of these isotopic systems in mind, by combining isotopic proxies, including published data from more traditional proxies focused on broad insights into climatic and environmental conditions, it is possible for us to compare, in detail, the diets and niches of different taxa across space and time (see text S2). The studied period (from 148 to 117 ka at Coc Muoi to 38.4 to 13.5 ka at Tam Hay Marklot) spans the Last Interglacial (127 to 119 ka) and in large part the Last Glacial Period (119 to 11.7 ka) (*58*), characterized by an overall cooling trend with repeated shifts between stadials (cold phases) and interstadials (warmer climate phases) (*59*), before the rapid warming at the onset of the Holocene. Thus, the sample set covers some of Southeast Asia’s major premodern Quaternary climatic changes and enables the investigation of different taxa’ ecological responses to these changes. Although the relative stability of plant communities across the Last Glacial Period is supported by a lack of major faunal turnover (*60*, *61*), global extinctions and regional extirpations of some large-bodied species are still attested for the region, thus demonstrating that these climatic changes were not without effect on faunal communities, either in themselves or in combination with a variety of factors [e.g., habitat fragmentation, reduced resource availability, interspecific competition (*62*)], including expanding human populations (*63*, *64*). While δ^13^C and δ^18^O values have been used to study climatic changes (*10*), the equifinality of these proxies makes it difficult to tease climatic and baseline impacts from animals’ foraging and dietary behavior. Here, the multi-isotope dataset assembled across space and time, notably including δ^66^Zn and δ^15^N_enamel_ values alongside these more traditional proxies, offers unprecedented resolution of the dynamic and highly variable responses from animals.

**Fig. 5.**
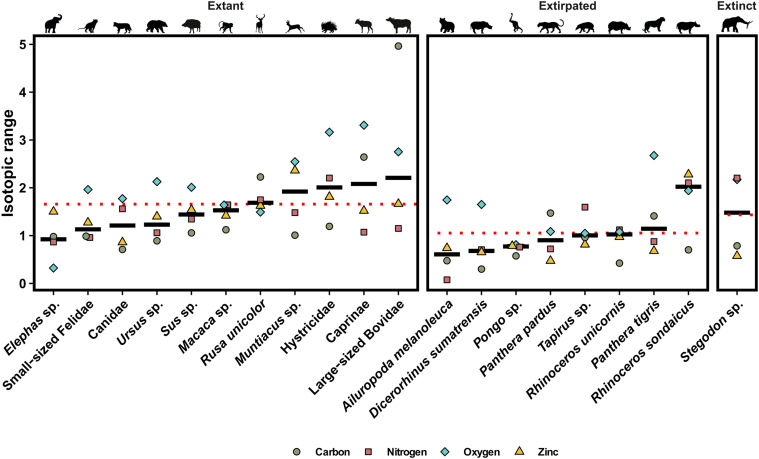
Average isotopic range of δ^15^N_enamel_ (‰ AIR), δ^66^Zn (‰ JMC-Lyon), δ^13^C (‰ VPDB), and δ^18^O (‰ VPDB) of extant, locally extirpated, and extinct taxa for the studied period. All isotopic values are standard-score transformed to allow direct comparison between the different isotope systems. Taxa found at only a single site were excluded as their isotopic ranges only capture intrasite variability rather than temporal changes across sites. The range of every isotopic system was calculated for each taxon at each site (Tam Hay Marklot, Pà Hang Mountain, Duoi U’Oi, and Coc Muoi) and across sites (i.e., absolute maximum—absolute minimum), and then averaged into a single value for each isotopic system. The ranges calculated from a single individual (i.e., *n* = 1 for any given taxa at a given site) were excluded. The δ^13^C values were converted to those of the food web’s primary carbon sources for better comparability between taxa (see text S1). Both δ^13^C and δ^18^O values are taken from Bacon *et al.* (*10*, *46*) and Bourgon *et al.* (*27*, *28*). The δ^66^Zn values from Tam Hay Marklot are taken from Bourgon *et al.* (*27*), and those from Tam Pà Ling and Nam Lot from Bourgon *et al.* (*28*). The δ^15^N_enamel_ values from Tam Hay Marklot are taken from Leichliter *et al.* (*19*). The δ^66^Zn values of Coc Muoi are normalized to the average δ^66^Zn value of all other Southeast Asian sites (i.e., Tam Hay Marklot, Pà Hang Mountain, and Duoi U’Oi). The median range of each taxa is represented by a bold horizontal line, and the red dotted line represent the average range for each panel (extant, locally-extirpated, and extinct).

**Fig. 6.**
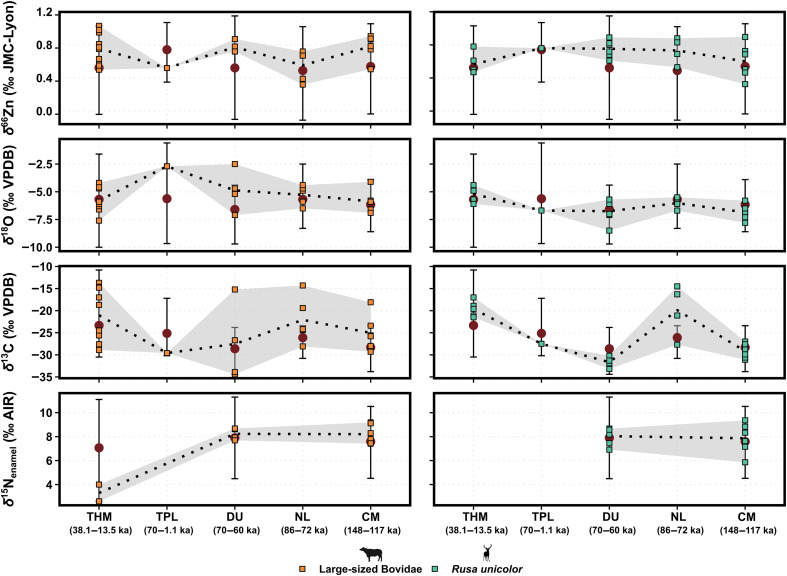
The δ^15^N_enamel_ (‰ AIR), δ^66^Zn (‰ JMC-Lyon), δ^13^C (‰ VPDB), and δ^18^O (‰ VPDB) values of large-sized bovids and sambar deer (*R. unicolor*) separated by sites. The large-sized bovids’ heterogeneous δ^13^C and δ^18^O values but homogenous δ^66^Zn and δ^15^N_enamel_ values suggest a strict diet, composed primarily of herbaceous plants and grasses given the C_4_-indicative δ^13^C values and the high δ^66^Zn and δ^15^N_enamel_ values. In the case of the sambar deer, the δ^66^Zn and δ^15^N_enamel_ values capture different plant resources consumed, while the low and narrow range of δ^13^C values associate them with understory plants. The δ^13^C values were converted to those of the food web’s primary carbon sources for better comparability between taxa (see text S1), and both δ^13^C and δ^18^O values are taken from Bacon *et al.* (*10*, *46*) and Bourgon *et al.* (*27*, *28*). The δ^66^Zn values from Tam Hay Marklot are taken from Bourgon *et al.* (*27*) and those from Tam Pà Ling and Nam Lot from Bourgon *et al.* (*28*). The δ^15^N_enamel_ values from Tam Hay Marklot are taken from Leichliter *et al.* (*19*). The δ^66^Zn values of Coc Muoi are normalized to the average δ^66^Zn value of all other Southeast Asian sites (i.e., Tam Hay Marklot, Pà Hang Mountain, and Duoi U’Oi). The whiskers represent the full range of values for all animals of each site (excluding outliers), and the red dots correspond to the mean of each site. The dotted line is the moving average of each taxa’ values between sites, while the shade area delimits the maximum and minimum values. Tam Hay Marklot is denoted with the abbreviation THM, Tam Pà Ling with TPL, Duoi U’Oi with DU, Nam Lot with NL, and finally, Coc Muoi with CM.

Our results show that species’ responses to changing environments in the tropical Southeast Asian context varied through the Last Interglacial and the Last Glacial Period, periods of lower precipitation and increased seasonality (*65*). Notably, taxa that still have extant counterparts in the study region share some degree of ecological flexibility, as illustrated by the large range in their isotopic values (Fig. 5), which is overall larger than that of taxa now extinct or extirpated from the region [linear mixed-effects model (LMM): β = 0.61, *P* = 0.01; tables S9 and S10]. Moreover, dietary categories do not appear to drive variation (*P* = 0.31), and taxon-level variation (included as a random effect) accounted for only a moderate proportion of variance (σ^2^ = 0.074, σ = 0.272). While the number of studied individuals per taxon, as well as their alpha-taxonomic assignment (i.e., species or genus level), can affect the range observed in their isotopic values, randomized resampling nonetheless suggests that extirpated taxa have lesser ecological flexibility (fig. S8, and, for a comprehensive overview of data normalization approaches and robustness checks, see text S3). This flexibility observed in extant taxa can be expressed as one or more of the following: (i) foraging on two or more trophic levels (i.e., omnivorous behaviors, attested by δ^66^Zn and δ^15^N_enamel_ values), (ii) foraging in different ecosystems (e.g., open and closed environments, mostly attested by δ^13^C values), or (iii) foraging on multiple resources of a trophic level (e.g., herbivorous taxa, essentially ruminants, consuming different plant species and plant organs; mostly attested by δ^66^Zn, δ^15^N_enamel_, and to a lesser degree δ^18^O values) (see text S2 for a detailed description of isotopic values for each extant taxa). For instance, the large-sized bovids exhibit variability in δ^13^C and δ^18^O values, with some values indicative of C_4_ plant consumption (i.e., foraging in different ecosystems), but a comparatively more restricted range in δ^66^Zn and δ^15^N_enamel_ values (Fig. 6). In contrast, the sambar deer (*Rusa unicolor*) show a very narrow range of δ^13^C values but relatively heterogenous δ^66^Zn and δ^15^N_enamel_ values (i.e., foraging on multiple resources of a trophic level), especially at Coc Muoi (Fig. 6). Large-sized bovid δ^66^Zn values suggest a strict diet on low-growing plants (e.g., grasses and shrubs) but foraged in diverse habitats, as indicated by the δ^13^C values, while the sambar deer’s δ^66^Zn values imply a variety of plant resources consumed, even when δ^13^C values suggest that they remained strictly in densely forested habitats. These two cases exemplify well different forms of dietary flexibility but also the need to decouple habitat-driven environmental signals and actual dietary intake when using δ^13^C as a proxy. This, consequently, highlights the benefits of performing multi-isotope investigations and how they could provide additional ecological insights into conservation efforts.

In contrast to Pleistocene taxa still found in the study region, taxa with extirpated modern counterparts exhibit dietary specialization characterized by narrow ranges of isotope values throughout the studied period (Fig. 5). The ecological responses of extirpated modern taxa appear limited; the narrow δ^13^C and δ^18^O ranges of these taxa indicates a close affinity to or preference for a given habitat, while their δ^66^Zn and δ^15^N_enamel_ values suggest a somewhat specialized diet (see text S2 for a detailed description of isotopic values for each extirpated taxon). One exception among the extirpated taxa is the Javan rhinoceros (*R. sondaicus*), which exhibits a high δ^66^Zn and δ^15^N_enamel_ variability (one of the highest of any taxon) and suggests diversity in its diet. This dietary flexibility conforms well with its modern behavior, showing them to be more adaptable feeders than other rhinoceros species, and aligns more closely with the extant foraging and dietary behavior of this taxon (*66*). One possible explanation for the species extirpation from Laos and Vietnam is likely attributable to the illegal and excessive demand for rhinoceros horn and other products for alternative medical practice in more recent times (*67*).

**Fig. 7.**
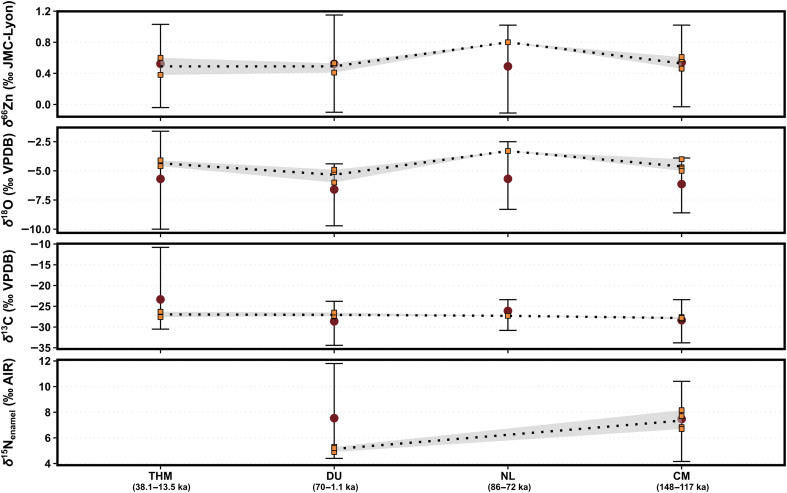
The δ^15^N_enamel_ (‰ AIR), δ^66^Zn (‰ JMC-Lyon), δ^13^C (‰ VPDB), and δ^18^O (‰ VPDB) values of orangutan (Pongo sp.) between sites. Orangutans’ δ^13^C values remain very close to one another for the whole studied period, possibly suggesting a specific habitat of forest environments. The δ^66^Zn values follow δ^18^O values very closely, with the highest δ^66^Zn value among orangutans from Nam Lot’s specimen exhibiting the highest δ^18^O values. Last, the δ^15^N_enamel_ values display a stark difference between Coc Muoi and Duoi U’Oi, with lower values at the latter also associated with the lowest δ^18^O values among orangutans. The δ^13^C values are converted to those of the food web’s primary carbon sources for better comparability between taxa [see text S1 and (*28*) for details], and both δ^13^C and δ^18^O values are taken from Bacon *et al.* (*10*, *46*) and Bourgon *et al.* (*27*, *28*). The δ^66^Zn values from Tam Hay Marklot are taken from Bourgon *et al.* (*27*) and those from Nam Lot from Bourgon *et al.* (*28*). The δ^66^Zn values of Coc Muoi are normalized to the average δ^66^Zn value of all other Southeast Asian sites (i.e., Tam Hay Marklot, Pà Hang Mountain, and Duoi U’Oi). The whiskers represent the full range of values for each site, and the red dots correspond to the mean of each site. The dotted line is the moving average of orangutans’ values between site, while the shade area delimits the maximum and minimum values for orangutans found between two sites. Tam Hay Marklot is denoted with the abbreviation THM, Duoi U’Oi with DU, Nam Lot with NL, and finally, Coc Muoi with CM.

A notable case within the extirpated taxa is that of the orangutan (*Pongo* sp.), whose three modern species’ populations (*Pongo pygmaeus*, *Pongo abelii*, and *Pongo tapanuliensis*) are now solely found on the islands of Borneo and Sumatra but formally covered a much larger geographical area from southern China to Java (*68*, *69*). Orangutan δ^66^Zn values are very similar across sites (Fig. 7), except for higher values found at Nam Lot. No published δ^66^Zn values are available for fruits, but, as lighter Zn isotopes are typically remobilized to growing organs (*52*), the generally low δ^66^Zn values compared to herbivorous taxa could suggest a diet including fruits as per modern ecological observation (*70*–*72*), or even more than modern Bornean *P. pygmaeus* (*73*), with Nam Lot’s higher values likely indicating a diet with more varied plant parts. Orangutan δ^66^Zn values follow very closely δ^18^O values [adjusted *R*^2^ = 0.35, *F*(1, 8) = 5.941, *P* = 0.04], supporting the observed correlation between the δ^66^Zn values in leaves and the height of the plants (*74*–*76*). Moreover, it suggests an unprecedented potential in the multi-isotope study of stable isotope vertical stratigraphy within a rainforest canopy, perhaps especially for the study of feeding niches in arboreal primates. Although available for only two sites, the δ^15^N_enamel_ values display a stark difference between Coc Muoi and Duoi U’Oi, with lower values at the latter by roughly 2‰, which could indicate a diet comprising more leaves or even woody elements (*49*).

Orangutan δ^13^C values show a small range (Fig. 7), similar to what was observed for the extinct *Pongo weidenreichi* during the Middle to Late Pleistocene (*23*), suggesting a specialized closed canopy niche and supporting the argument that maintaining a given habitat is the most crucial factor in orangutan distribution (*77*). Overall, our data offer additional insights into fossil orangutans’ dietary behaviors, which appear to have remained in a strict ecological niche of canopy forest with a diet predominantly composed of fruits but with fallback plant resources in drier periods, like that represented at the site of Duoi U’Oi marked by a change in forest composition with more temperate elements (*10*). These results closely mirror the behaviors of modern orangutans as, while they are predominately frugivores (*70*, *71*, *78*, *79*), their diet nonetheless includes the largest range of plant species of all the great apes (*79*) and can also include a variety of food types during periods when fruits are unavailable, including insects and occasionally small mammals (*72*, *78*, *79*). Nevertheless, amid modern-day large-scale deforestation, stable orangutan populations have been shown to persist only in landscapes with sufficient forest cover (*80*), and the limited ecological responses to changing climate and environments in the past highlight this as a long-term trend in the species’ population viability.

Our dataset contains little data on completely extinct taxa. The only two are the giant tapir (*M. augustus*) and the stegodon (*Stegodon* sp.), which are characterized by small sample sizes due to their low abundance in the assemblages, making assessments of their ecological response to climatic changes somewhat difficult. The giant tapir’s values are fairly similar to the tapirs’ [multivariate analysis of variance (MANOVA) test: Pillai’s trace = 0.73, *F*(4, 3) = 2.05, *P* = 0.29], suggesting a similar forest understory niche for that species, as reported elsewhere (*10*, *81*, *82*). However, giant tapir δ^15^N_enamel_ values are noticeably lower than those of the tapir, by around 3‰ and among the lowest values for both sites, and could suggest a large proportion of stems and bark in its diet (*49*, *50*). As for the stegodon, its δ^13^C and δ^18^O values are similar to the elephants’ (*Elephas* sp.) [MANOVA test: Pillai’s Trace = 0.83, *F*(4, 1) = 1.24, *P* = 0.58], as is often the case (*10*, *81*, *82*). While δ^13^C data have been used to argue that fossil elephants were mixed feeders in contrast to stegodons (*82*), individuals of both species predominantly exhibit low δ^13^C values indicative of canopy understory foraging (*10*, *81*, *82*). In the case of Coc Muoi and Duoi U’Oi, the slightly larger variability in δ^66^Zn values could suggest a more diversified diet for the elephants, but the same could be argued for the stegodon by the large difference in its δ^15^N_enamel_ values (3.5‰). Ultimately, the restricted sample size precludes solid assessment for both giant tapir and stegodon, but we are confident that additional δ^66^Zn or δ^15^N_enamel_ values could help elucidate the extinction of these very large herbivores, whose lack of dietary flexibility is often cited as a reason for their demise (*68*, *82*).

Overall, a prevailing diversification of resources consumed is often observed for species still present in the region today, although this is not intrinsically associated with a diversification in habitat niches. The isotopic variability among species with extant counterparts likely reflects two context-dependent ecological responses to climate-driven habitat shifts. Some taxa exhibit greater isotopic variability during periods favoring lowland rainforests (e.g., *Muntiacus* sp., *Macaca* sp., and *Ursus* sp.), possibly due to increased dietary opportunities associated with higher resource diversity. Others (e.g., Canidae, Caprinae, and Hystricidae), by contrast, display reduced isotopic variability in those same periods but broaden their dietary niche during intervals favoring more open, seasonal forests. Some taxa (e.g., *Sus* sp., *R. unicolor*, and large-sized Bovidae) also show both behavior. While these responses may appear contradictory, both likely represent forms of context-dependent niche plasticity, either through niche expansion under reduced competition (ecological release) or through dietary broadening in response to competitive displacement (*83*–*86*). Ecological generalism or specialism is not fixed but rather modulated by habitat structure, resource availability, and interspecific interactions (*85*, *86*). The fact that these dual responses are observed exclusively in taxa with extant representatives further suggests that dietary flexibility may have played a key role in their persistence across climatic fluctuations, but also highlights the need for time-transgressive assessments of species, as their flexibility may change through time.

In contrast, extirpated taxa exhibit limited variability in their isotope values over the studied period. The small sample size for some of these taxa hinders more thorough assessments. Additional work is required to better understand individual species’ ecological flexibility, specifically because of the general paucity of isotope data for modern-day species in Southeast Asia. By integrating multiple isotopic proxies, we capture a more nuanced picture of ecological adaptation than would be possible through trophic level (δ^66^Zn and δ^15^N_enamel_) or environmental (δ^13^C) indicators alone. However, interpreting multi-isotope datasets presents several challenges, particularly when combining proxies that differ in ecological resolution, statistical properties, and scale. For instance, δ^13^C values often exhibit bimodal distributions due to C_3_/C_4_ plant distinctions, while δ^66^Zn and δ^15^N values reflect more relative ecological gradients. These differences complicate direct comparisons and require careful consideration of normalization methods and statistical assumptions. In addition, equifinality, site-specific baselines, sample size, and species-specific ecology can all influence observed variability and should be considered when evaluating ecological patterns across systems.

Moreover, obtaining insights from regions where species have persisted (e.g., orangutans in Sumatra and Borneo) will be key in understanding how locally extirpated animals adapted or, perhaps did not, to a changing environment. While the current dataset offers the chance of assessing ecological response through time under similar clinal and environmental conditions, additional data (including from modern specimens) could help better assess the full scale of ecological responses of each species and perhaps highlight causes of extirpation on a much wider scale, including the potential influence of past human activities. Future studies should also aim to evaluate the potential role of past anthropogenic pressures, most notably hunting pressure but also landscape modification (e.g., fire regime), in shaping these extinction and extirpation patterns, particularly in relation to ecological and climatic factors.

While rates of extirpation and extinction likely vary across taxa (*87*) due to a variety of factors (e.g., life history, gestation period, body size, etc.), the current accelerated pace of climate change and habitat loss poses a substantial threat to many (*88*). Despite this, the relation between ecological flexibility and the presence of extant relatives remains relevant and highlights the vulnerability of mammals such as Canidae (dhole, *Cuon alpinus*), small-sized Felidae (possibly clouded leopards, *Neofelis nebulosa*, or leopard cats, *Prionailurus bengalensis*), and elephants (*Elephas* sp.). Our results further emphasize the precariousness of certain taxa, highlighting their inflexibility to adapt to changing environments, an issue that will become increasingly problematic in the 21st century amid anthropogenic impacts on climate and tropical environments (*5*, *6*, *88*). Moreover, even those mammals that did adapt flexibly in the past are likely to come under pressure in the face of increasingly rapid land use changes, which inhibit mobility or niche flexibility beyond anthropogenic settings. Southeast Asia holds the highest number of endemic species in the world (*6*), but it is also home to the highest number of threatened species and faces the greatest rate of deforestation of any tropical region (*5*). The Republic of Singapore notably illustrates well the consequences and impacts of large-scale deforestation, having experienced more than 95% habitat loss and a decline of more than 28% of its biodiversity (with 881 recorded species) over the past decades (*89*, *90*). It has been shown that habitat loss, fragmentation, and deforestation lead to considerable loss of biodiversity and species abundance (*6*). Preserving the remaining habitats to reduce further losses is thus paramount, but the effectiveness of such actions relies on robust policies and effective local law enforcement, which are often insufficient (*91*). Developing a long-term multi-isotope ecological assessment for threatened species could help bridge the gap for adequate conservation efforts.

## MATERIALS AND METHODS

### Site descriptions and dating

The fossil localities examined in this study are located in northern Vietnam (Coc Muoi and Duoi U’Oi) and northeastern Laos (Tam Pà Ling, Nam Lot, and Tam Hay Marklot), all within the 23° to 20°N latitudinal belt (*27*, *44*, *46*–*48*). These sites are situated in classic tower karst landscapes, where caves formed in massive Carboniferous to Triassic limestone. Sedimentary infillings are consistent across sites and include well-cemented breccias adhering to cave walls and ceilings, as well as silty to sandy clays covering the cave floors. These deposits were largely transported and deposited via endokarstic processes, with contributions from exokarstic sources (*92*).

#### Coc Muoi, Northeast Vietnam

Situated at 361-m elevation in Lang Son province, Coc Muoi yielded sandy clay deposits dated by SG-OSL and pIR-IRSL to between 148 to 117 ka (*44*).

#### Duoi U’Oi, Northwest Vietnam

Located near the Laos border, this lowland site [~5 m above sea level (a.s.l.)] produced muddy calcareous breccias, containing faunal assemblage, and calcitic floor dated to 70 to 60 ka via TL, SG-OSL, and U-series dating (*46*, *47*).

#### Tam Hay Marklot, Northeast Laos

Faunal remains were recovered from sandy to gravelly clays covering most of the cave floor. Combined ESR/U-series dating of five teeth yields an age range of 38.4 to 13.5 ka (*27*).

#### Nam Lot, Northeast Laos

This high-elevation site (1,120 m a.s.l.) on Pà Hang Mountain contains breccia and silty to sandy clays. TL, SG-OSL, and U-series dating suggest the fauna dates to 86 to 72 ka (*46*).

#### Tam Pà Ling, Northeast Laos

Located at 1170 m a.s.l. atop Pà Hang Mountain in Huà Pan Province, TPL is a high-elevation cave formed in Carboniferous-Permian limestone. The site preserves finely laminated silty clay and sandy deposits, primarily transported through low-energy slopewash from the cave entrance. Sediments span a long chronostratigraphic range (86 to 1.1 ka) and have been dated using multiple methods, including U-series, OSL, pIR-IRSL, ESR, and ^14^C [Freidline *et al.* (*48*) and reference therein]. Full description of sites and dating, nature of the fossiliferous deposits, and fossil assemblages and taphonomy, can be found in the original publication and references therein (*27*, *44*, *46*–*48*).

### Sample collection and sampling

Fossil material analyzed in the current study includes only specimens examined for isotopic Zinc data from the Coc Muoi and Duoi U’Oi cave sites in northern Vietnam, alongside previously published material from Nam Lot, Tam Pà Ling, and Tam Hay Marklot (*27*, *28*, *46*). The Coc Muoi fossils were excavated between 2013 and 2015 during collaborative fieldwork conducted by a Vietnamese-French team, under the invitation and supervision of the Vietnamese authorities (the Institute of Archaeology in Hanoi and the Lang Son Museum). The Duoi U’Oi material was recovered during a previous campaign in December 2003 under a Vietnamese-French-Japanese collaboration.

Specimens of the fauna were taxonomically identified by A.-M.B. (Université Paris Cité, France) and P.-O.A. (Institut des Sciences de l’Évolution de Montpellier, France) in collaboration with the members of the Anthropological and Palaeoenvironmental Department of the Institute of Archaeology, Ha Noi, Vietnam. No additional authentication procedures were required beyond standard zooarchaeological identification, and the processing was carried out in collaboration with national heritage authorities. All fossil remains from Coc Muoi and Duoi U’Oi are respectively housed at the Lang Son Museum in Lang Son province and the Institute of Archaeology in Hanoi. These collections are accessible to researchers upon request and in accordance with Vietnamese regulations.

In this study, animals were grouped on the basis of their broad dietary behaviors (carnivorous, omnivorous, and herbivorous). Omnivore taxa are defined for the purpose of this study as animals that exhibit a systematic degree of consumption of animal matter (invertebrates or vertebrates) for sustenance purposes, whether it is regular, minor, or solely seasonal. In addition, all taxa were also classified into three distinct groups based on their latest (i.e., at the time the current study was performed) modern-day conservation status assessment in the region of interest (Laos and Vietnam) as listed by The IUCN Red List of Threatened Species (https://iucnredlist.org): extant, extirpated, and extinct. Extirpation and extinction rates are expected to differ among taxa (*87*, *88*) due to various factors such as life history traits, gestation periods, body size, climate change, and habitat destruction, and the timing of extirpation and extinction of species varies. Nevertheless, grouping species based on the current status (extant, extirpated, and extinct) of their modern counterpart allows for an overview of a species’ isotopic variability and vulnerability. In the case of the current dataset, the following taxa are defined as having extant modern-day counterparts in the studied region: large-sized Bovidae, small-sized Felidae, Hystricidae, *Muntiacus* sp., *Macaca* sp., *Sus* sp. *Ursus* sp., Caprinae, *R. unicolor*, *Elephas* sp., and Canidae (see text S2). The “extirpated” group is composed of the following: *A. melanoleuca*, *Pongo* sp., *Tapirus* sp., *Panthera pardus*, *Panthera tigris*, *R. sondaicus*, *R. unicornis*, and *D. sumatrensis* (see text S2). The “extinct” group includes only two taxa: *M. augustus* and *Stegodon* sp.

Before sampling, each tooth was subjected to light mechanical cleaning using a handheld drill equipped with a diamond-tipped burr to remove both adhering external material and a superficial layer of enamel. Around 20 mg of tooth enamel powder samples from 19 different mammalian taxa (among Artiodactyla, Perissodactyla, Proboscidea, Carnivora, Primates, and Rodentia) of Coc Muoi (*n* = 84) and Duoi U’Oi (*n* = 60) fossil assemblages (table S1) were then taken from the full height of the crown to obtain an average dietary signal for the time of tooth mineralization, using a handheld drill using a diamond-tipped burr. Regions showing any visible signs of discoloration, porosity, or possible secondary carbonate deposits were intentionally avoided during sampling to minimize the potential impact of diagenesis. These bulk samples were then homogenized and separated into two aliquots for the various isotope analyses: 5 to 10 mg for zinc and 5 to 10 mg for nitrogen.

In addition, already-published δ^66^Zn data from northern Laos from the MIS 3–2 Tam Hay Marklot [38.4 to 13.5 ka (*27*)] and the Pà Hang Mountain composed of data from both the MIS 5 Nam Lot [86 to 72 ka (*28*, *46*)], and those from the long stratigraphic sequence at Tam Pà Ling [70 to 1.1 ka (*28*)], and a few δ^15^N_enamel_ values from Tam Hay Marklot (*19*) were used for the current study (table S4).

### Zinc chemical purification and measurement

Following previous key publications describing the method (*24*, *93*), samples were subjected to chemical purification in a clean laboratory using acids and ion exchange resins, whereby the element of interest (i.e., Zn) is collected. This desired separation is achieved by combining specific acids, molarity, and resin, leading to different kinematics for each element traveling through the resin.

Enamel [11.05 ± 1.48 mg (1σ), *n* = 144], NIST SRM 1400 bone ash reference material (*n* = 9), and/or in-house AZE bovine liver reference material (*n* = 10) were digested in 1 ml of HCl 1.0 M, evaporated, and then redissolved in 1 ml of HBr 1.5 M. Zinc chemical purification was then achieved by ion-exchange column chromatography, using preconditioned microcolumns on 1 ml of AG-1x8 resin (200 to 400 dry mesh size, 106- to 180-μm wet bead size). Two milliliters of HBr 1.5 M was used for matrix residue elution, followed by 5 ml of HNO_3_ 0.5 M for Zn elution. Samples were then dried and redissolved in 1 ml of HBr 1.5 M to undergo the procedure again to fully remove the phosphate matrix. Last, samples were dried and redissolved in 1 ml of HNO_3_ 0.5 M before measurements. A procedural blank was also included and prepared alongside every chemical purification.

Zinc isotope measurements were performed on a Thermo Fisher Scientific Neptune Multi-collector inductively coupled plasma mass spectrometry at Max Planck Institute for Evolutionary Anthropology (Leipzig, Germany), using the Cu-doping protocol of Toutain *et al.* (*94*). Zinc isotope abundances are presented in δ (delta) notation expressed as deviation per mil (‰), as follows: δ^66^Zn = [(^66^Zn/^64^Zn_sample_)/(^66^Zn/^64^Zn_standard_) ─ 1] × 1000. The reference material Zn Alfa Aesar solution was used for standard bracketing, with reproducibility within a session of 0.02 (1σ). All δ^66^Zn values are expressed relative to the Johnson Matthey standard solution (JMC)-Lyon standard with a mass-dependent Alfa Aesar offset of +0.27‰ for δ^66^Zn (*24*, *95*). Reference material NIST SRM 1400 and in-house bovine liver AZE were prepared and analyzed with the samples, and respectively had δ^66^Zn values of 0.94 ± 0.02‰ (*n* = 9) and 1.59 ± 0.03‰ (*n* = 10) (see table S7), similar to reported values (*25*–*31*, *38*, *39*, *54*, *55*). Estimates for Zn concentrations of samples were obtained with a regression equation based on the ^64^Zn signal intensity (V) of three solutions with known concentrations (150, 300, and 600 μg/g). All samples and reference materials show a Zn mass-dependent isotope fractionation (i.e., the absence of spectral interferences) as δ^66^Zn versus δ^68^Zn values, respectively, onto lines with slopes close to the theoretical mass fractionation values of 2. The average Zn content from the chemistry blanks ranged from 0.56 to 3.81 μg/g [average = 1.32 ± 1.04 μg/g (1σ), *n* = 10]. Therefore, the isotopic composition measured for each sample and reference material is highly unlikely to have been influenced by the blanks, as the potential Zn contribution is too low [average 0.15% of the total Zn content for samples (average sample Zn content= 909 ± 1054 μg, *n* = 144)]. Repeated analyses of some specimens (*n* = 102) and reference materials (*n* = 11) were performed to determine the homogeneity of samples, and the overall average analytical repeatability for samples and reference material was ±0.02‰ (1σ).

### Nitrogen oxidation-denitrification and measurement

About 1.72 to 8.9 mg [5.97 ± 0.88 mg (1σ), *n* = 191] of tooth enamel samples of 144 specimens were measured for δ^15^N_enamel_ over seven analytical batches using the oxidation-denitrification method (*18*), including 47 replicates. Tooth enamel powder was first subjected to a reductive-oxidative cleaning to remove potential metal oxides and exogenous organic matter (*96*), and the remaining powder (after 10 to 90% sample loss) was demineralized using 4 M HCl to release endogenous organic matter (i.e., intra-and intercrystalline bound N), which was subsequently oxidized to nitrate using a persulfate oxidizing reagent (0.67 to 0.70 g of four times recrystallized potassium persulfate added to 4 ml of 6.25 M NaOH solution in 95 ml of Milli-Q water) (*97*). The resulting nitrate was then quantitatively converted to N_2_O via denitrifying bacteria (*Pseudomonas chlororaphis*) (*98*) and extracted for nitrogen isotopic composition on a custom multivalves system coupled to a Thermo Fisher Scientific 253 Plus isotope ratio mass spectrometer. While isobaric interference with CO_2_ could arise, this was avoided by two stages of cryo-isolation and gas chromatography columns (*98*, *99*), which result in the full separation of CO_2_ and N_2_O peaks, as seen in each sample chromatogram. International materials (USGS 40, USGS 65, USGS 34, and IAEA-NO-3), in-house coral standards (CF-1 and PO-2), and tooth enamel (AG-Lox and Mammy) were included in every run and each step of the method to allow monitoring measurement stability and the possibility of matrix-based effects during cleaning (see table S8). In addition, blank N concentration and δ^15^N_enamel_ were measured for each analysis batch and then used to correct N content and δ^15^N_enamel_ values of samples from the associated batch. Over the seven analytical batches, the blank N content was between 0.14 and 0.53 nmol/ml (average = 0.27 ± 0.11 nmol/ml), which corresponds to an average blank contribution of 3% [± 3% (1σ), *n* = 191]. Repeated analyses of some specimens (*n* = 47) were performed to determine the homogeneity of samples, and the overall average analytical repeatability for samples was ±0.76‰ (1σ).

### Statistical analyses

All statistical analyses were conducted using the open-source program R software [R version 4.0.2 (*100*)] using an alpha level for significance (i.e., *P* values) of 0.05. When applicable, preliminary tests and inspection of the data were conducted to ensure that the dataset fulfilled the specific tests’ assumptions (e.g., assumptions of normally distributed and homogeneous residuals), and *R*^2^ and *P* values were adjusted using multitesting correction when needed. To investigate isotopic variation between extant, extirpated and extinct taxon, we fitted an LMM. The model included status (extant versus extirpated and extinct) and diet (carnivore, omnivore, and herbivore) as fixed effects, with taxon as a random intercept to account for intraspecies variation. To help balance the model yet account for all data, extirpated and extinct taxa were grouped together as they both represent extinction (local versus global). Model parameters were estimated using restricted maximum likelihood, and *P* values were computed using Satterthwaite’s approximation. The following R packages were used in the current study: nicheROVER (*101*), tidyverse (*102*), cowplot (*103*), rstatix (*104*), lme4 (*105*), and lmerTest (*106*).

## Supplementary Materials

### This PDF file includes:

Figs. S1 to S30 Tables S1 to S13 Supplementary Text S1 to S3 References
