# Supplementary material for: Faunal persistence and ecological flexibility in Pleistocene Southeast Asia revealed through multi-isotope analysis
Source: Sci Adv. 2025 Oct 15;11(42):eadu3642. doi: 10.1126/sciadv.adu3642 (PMC12525774; doi:10.1126/sciadv.adu3642)
Supplement: Supplementary file 1 — Figs. S1 to S30 Tables S1 to S13 Supplementary Text S1 to S3 References [file sciadv.adu3642_sm.pdf]

Science Advances
AAAS

Supplementary Materials for

Faunal persistence and ecological flexibility in Pleistocene Southeast Asia revealed through multi-isotope analysis

Nicolas Bourgon *et al.*

Corresponding author: Nicolas Bourgon, bourgon@gea.mpg.de

Sci. Adv. **11**, eadu3642 (2025)
DOI: 10.1126/sciadv.adu3642

This PDF file includes:

Figs. S1 to S30
Tables S1 to S13
Supplementary Text S1 to S3
References

Supplementary Material – Figures

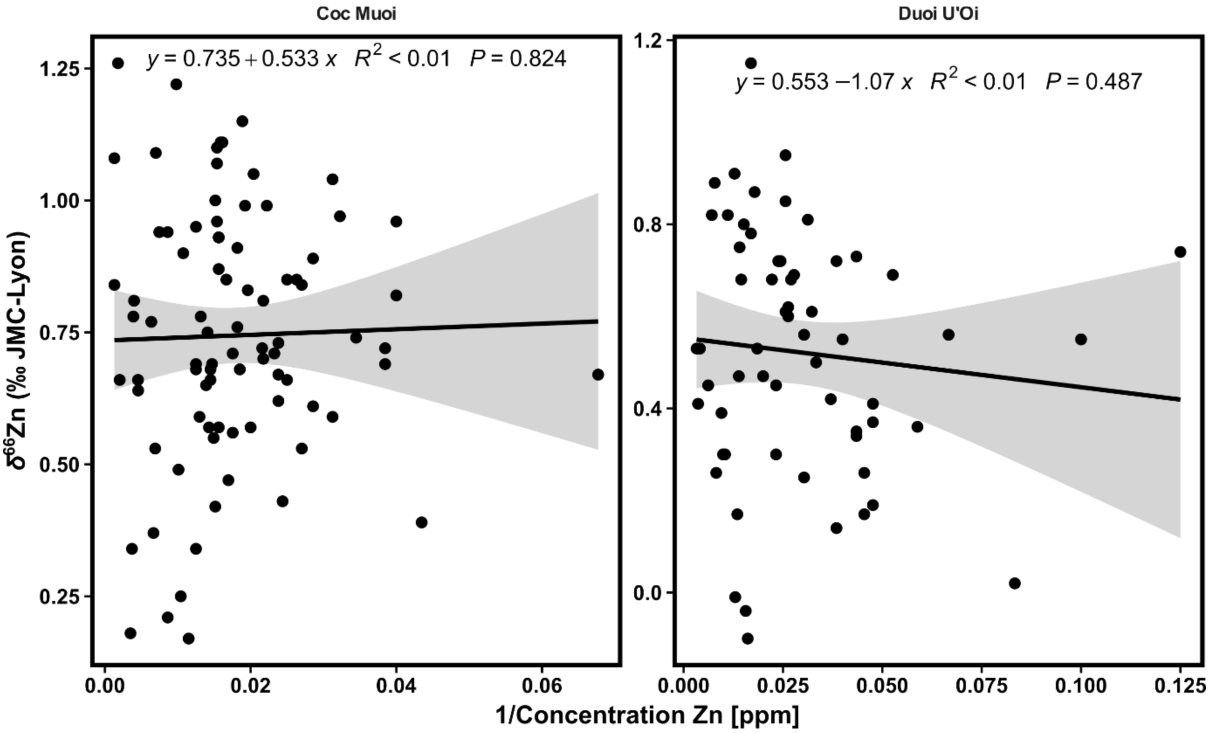

Figure S1. Zinc concentration and δ^{66}Zn values. Non-significant relationship between the Zn concentration (1/Conc [µg/g]) and δ^{66}Zn values in enamel of fossil teeth from Coc Muoi and Duoi U'Oi, with regression line, R^2, *p*-value, and confidence interval (shaded areas). Data is only separated by site and not by taxa, as sampling bias can affect concentrations; since the outer-most enamel layer is the most Zn-rich *(*107*)*, animals with thicker enamel, and thus potentially sampled at varying depths, are more likely to exhibit heterogeneous Zn concentration.

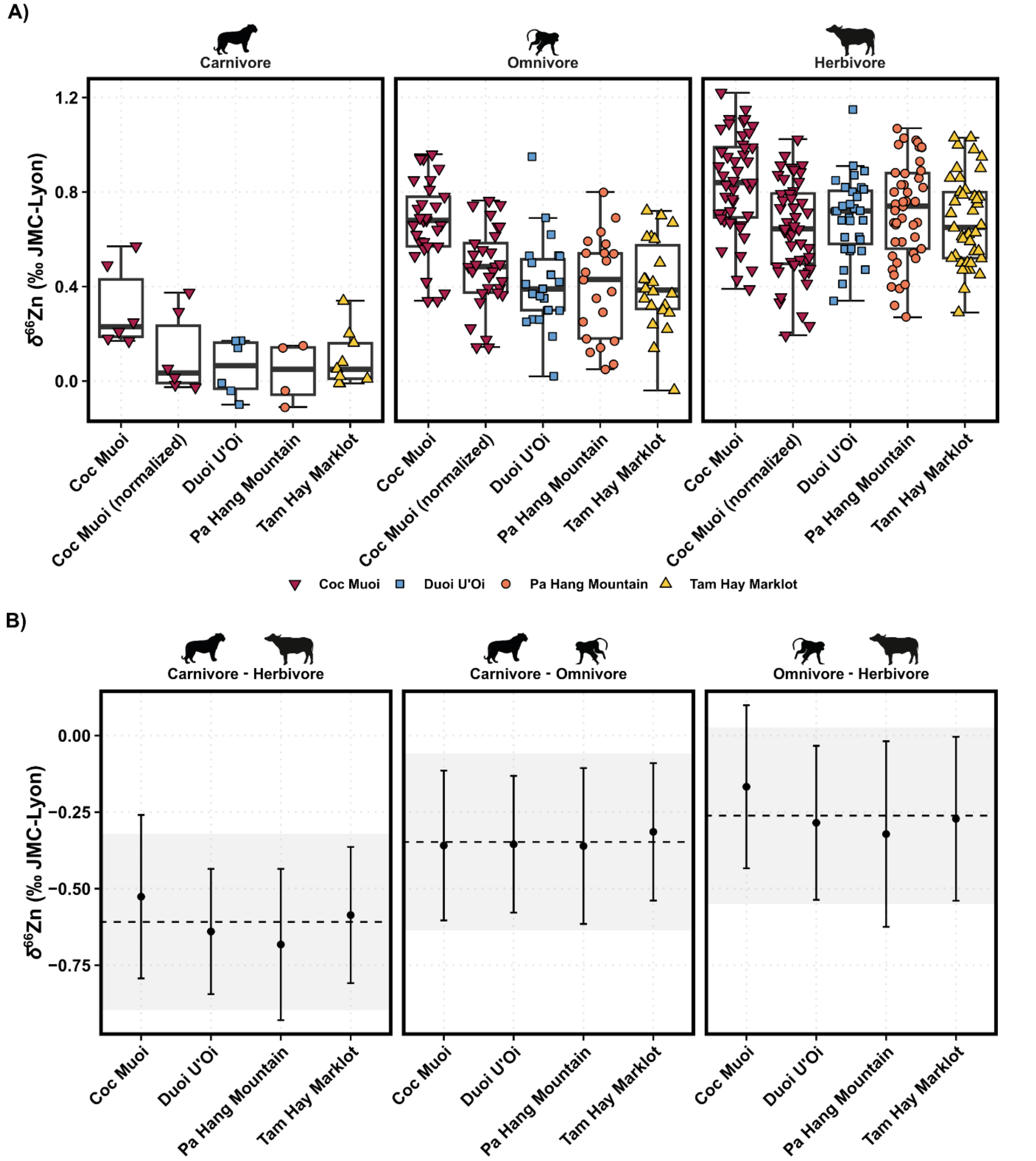

Figure S2. Comparison of (A) δ^{66}Zn values and (B) trophic spacing between sites and trophic level, including Coc Muoi with normalized value to the mean of all other sites. At the top a), boxplots presenting the range of δ^{66}Zn values (relative to the JMC-Lyon Zn isotope standard) of tooth enamel from each site for carnivores (excluding the bone-eating carnivore *Crocuta crocuta* from Nam Lot), omnivores, and herbivores. The color of each plot represents specimens coming from different sites: Coc Muoi (burgundy upside-down triangles), Duoi U'Oi (light blue squares), previously published data from Pà Hang Mountain (Nam Lot and Tam Pà Ling combined; orange circle), and Tam Hay Marklot (yellow triangles). The δ^{66}Zn values from Tam Hay Marklot are taken from Bourgon et al. *(*27*)* and those from Tam Pà Ling and Nam Lot from Bourgon et al. *(*28*)*. The boxes from the box and whisker plots represent the 25th–75th percentiles, with the median represented by a bold horizontal line. Below b), the δ^{66}Zn trophic spacings are expressed between combinations of dietary categories where each point represents the mean and the whiskers the standard deviation (1σ) for each site, whereas the dashed line shows the average and the shaded area shows the 95% confidence interval across sites.

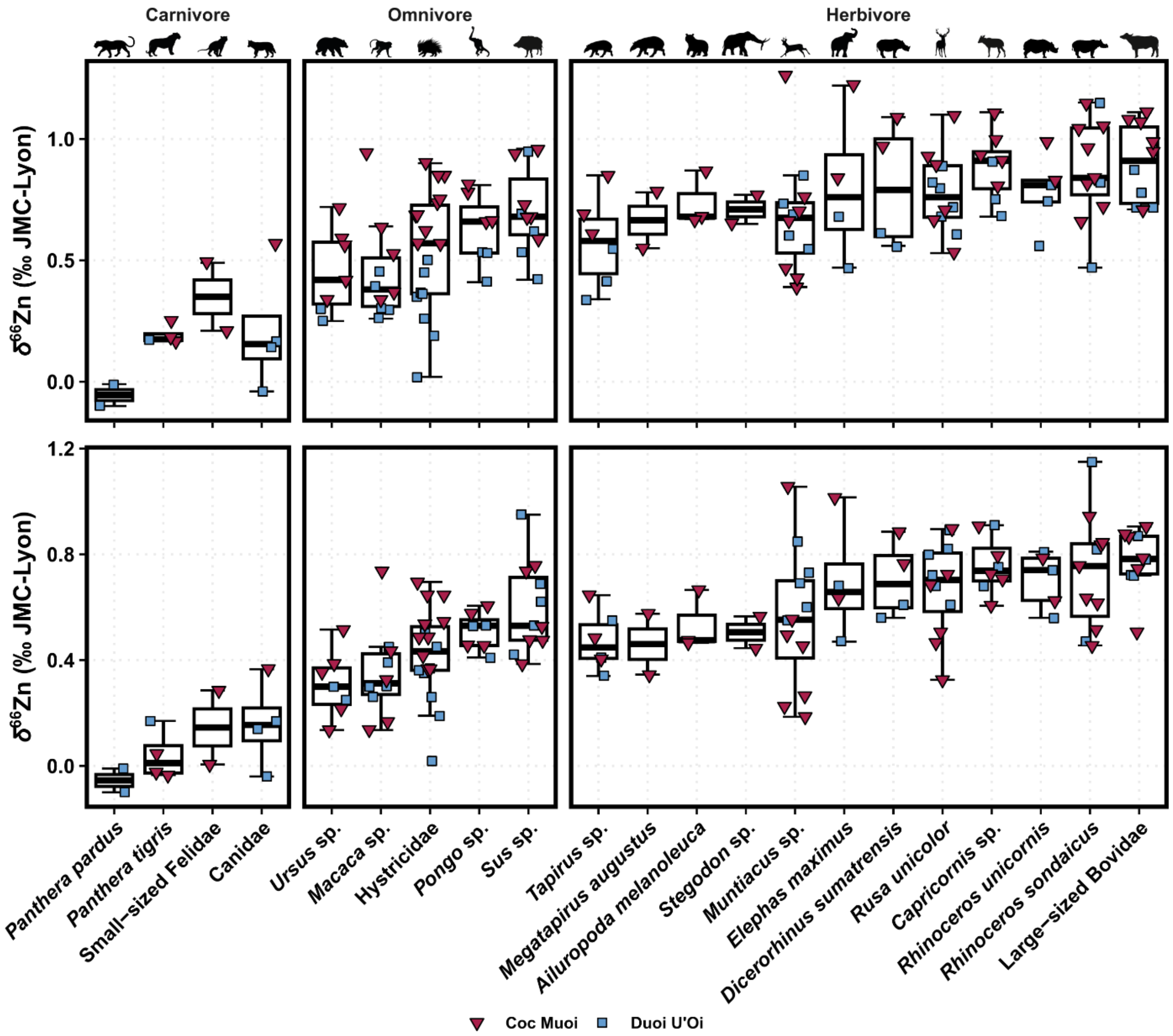

Figure S3. The δ^{66}Zn values from Coc Muoi and Duoi U'Oi, including Coc Muoi with normalized value to the mean of all other sites (below). Boxplots presenting the range of δ^{66}Zn values (relative to the JMC-Lyon Zn isotope standard) of tooth enamel from each site for carnivores (excluding the bone-eating carnivore *Crocuta crocuta* from Nam Lot), omnivores, and herbivores, both with Coc Muoi unstandardized values (top) and standardized values (bottom). The color of each plot represents specimens coming from different sites: Coc Muoi (burgundy upside-down triangles) and Duoi U'Oi (light blue squares). The boxes from the box and whisker plots represent the 25th–75th percentiles, with the median represented by a bold horizontal line.

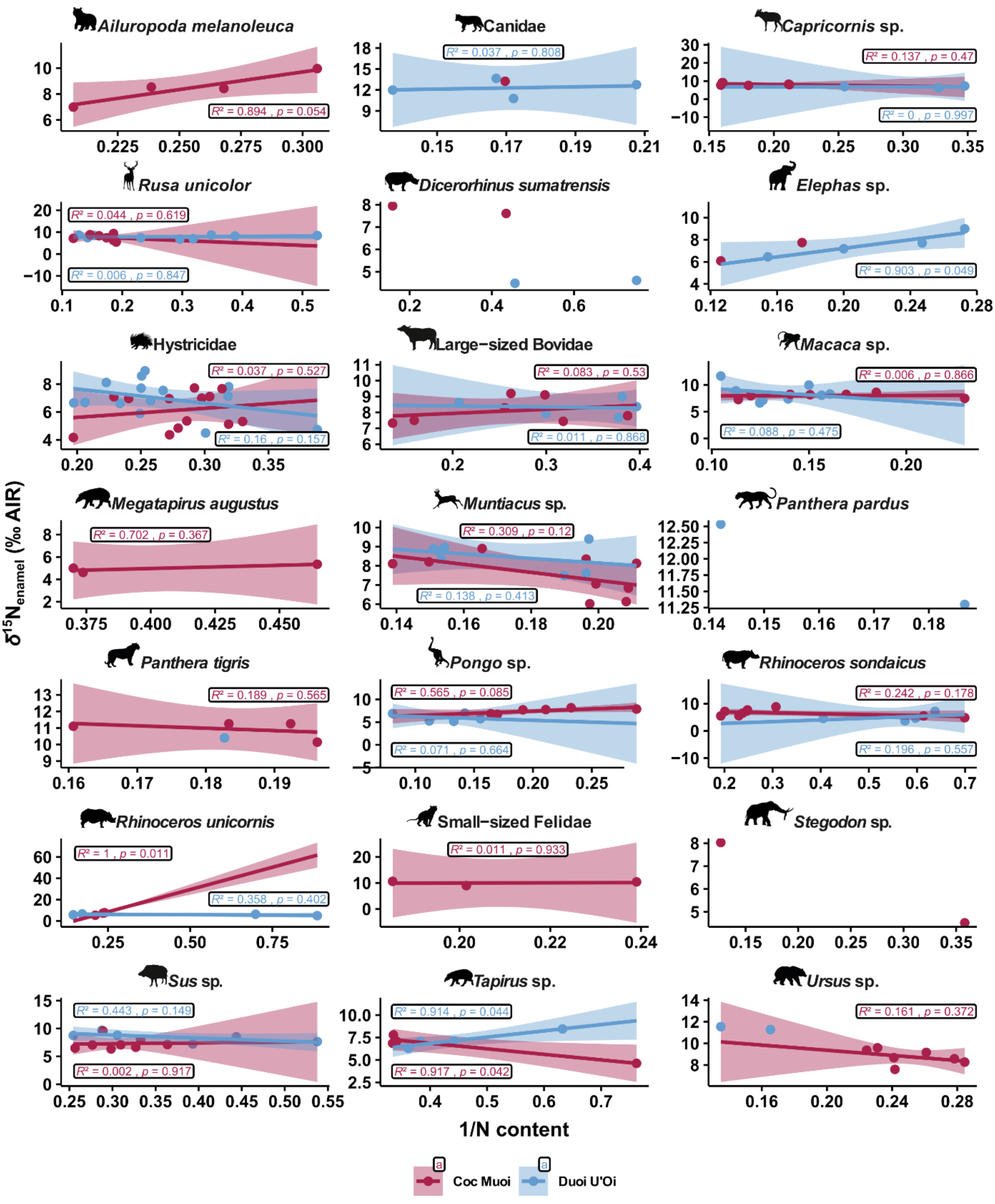

Figure S4. Nitrogen content and $\delta^{15}N_{enamel}$ values. Relationship between the N content (1/Content [nmol/ml]) and $\delta^{15}N_{enamel}$ values of fossil teeth for each taxon from Coc Muoi (red) and Duoi U'Oi (blue), with regression line, R^2, p-value, and confidence interval (shaded areas). While a few species (*Ailuropoda melanoneuca* and *Rhinoceros unicornis* from Coc Muoi, and *Tapirus indicus* from broth sites) show a significant relationship between N content (1/Content [nmol/ml]) and $\delta^{15}N_{enamel}$, the overall lack of consistent relationship within sites and across all taxa rather suggests that no post-mortem uptake occurred. Trend line, R^2, and p-values are not shown for taxa with too few observations ($n < 3$). Note that the scales of the y and x-axis vary from one facet (i.e., individual taxa plot) to another.

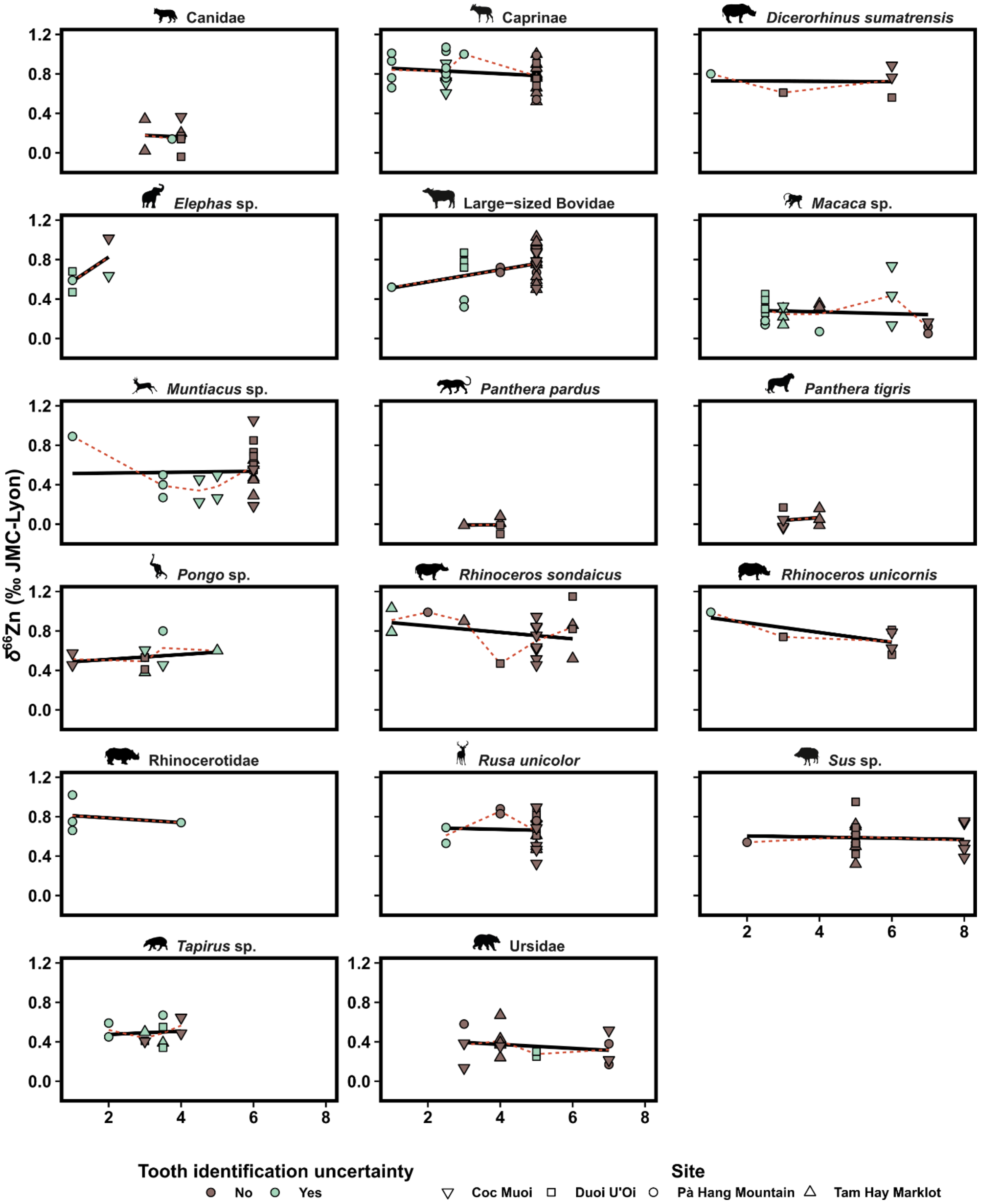

Figure S5. Relationship between δ^{66}Zn values and tooth eruption sequence. Relationship between δ^{66}Zn values (n = 236) and tooth formation sequence for all Southeast Asian sites: Coc Muoi (data normalized to the mean of all other sites), Duoi U'Oi, previously published data from Nam Lot and Tam Pà Ling, and Tam Hay Marklot. Tooth formation sequence was not available for all taxa *(*108–113*)* (1 being the earliest teeth formed), and some were assigned to phylogenetically-close species (e.g., *Bos taurus* for large-sized bovids). For each taxon, the black line represents the linear regression between δ^{66}Zn values and tooth formation sequence, while the red dashed line represents the moving average between δ^{66}Zn values and tooth formation sequence. Uncertainty in tooth position (e.g., "p/m", "m" rather than "m3") is expressed through points of different color: brown denotes uncertain tooth position (e.g., "p/m", "m", etc.) and teal denotes certain tooth position (e.g., "m3", "p2", etc.). Sites are denoted by different shapes: upside-down triangle for Coc Muoi, square for Duoi U'Oi, circle for Pà Hang Mountain (which includes Tam Pà Ling and Nam Lot), and triangle for Tam Hay Marklot. The δ^{66}Zn values from Tam Hay Marklot are taken from Bourgon et al. *(*27*),* and those from Pà Hang Mountain (Tam Pà Ling and Nam Lot) from Bourgon et al. *(*28*)*.

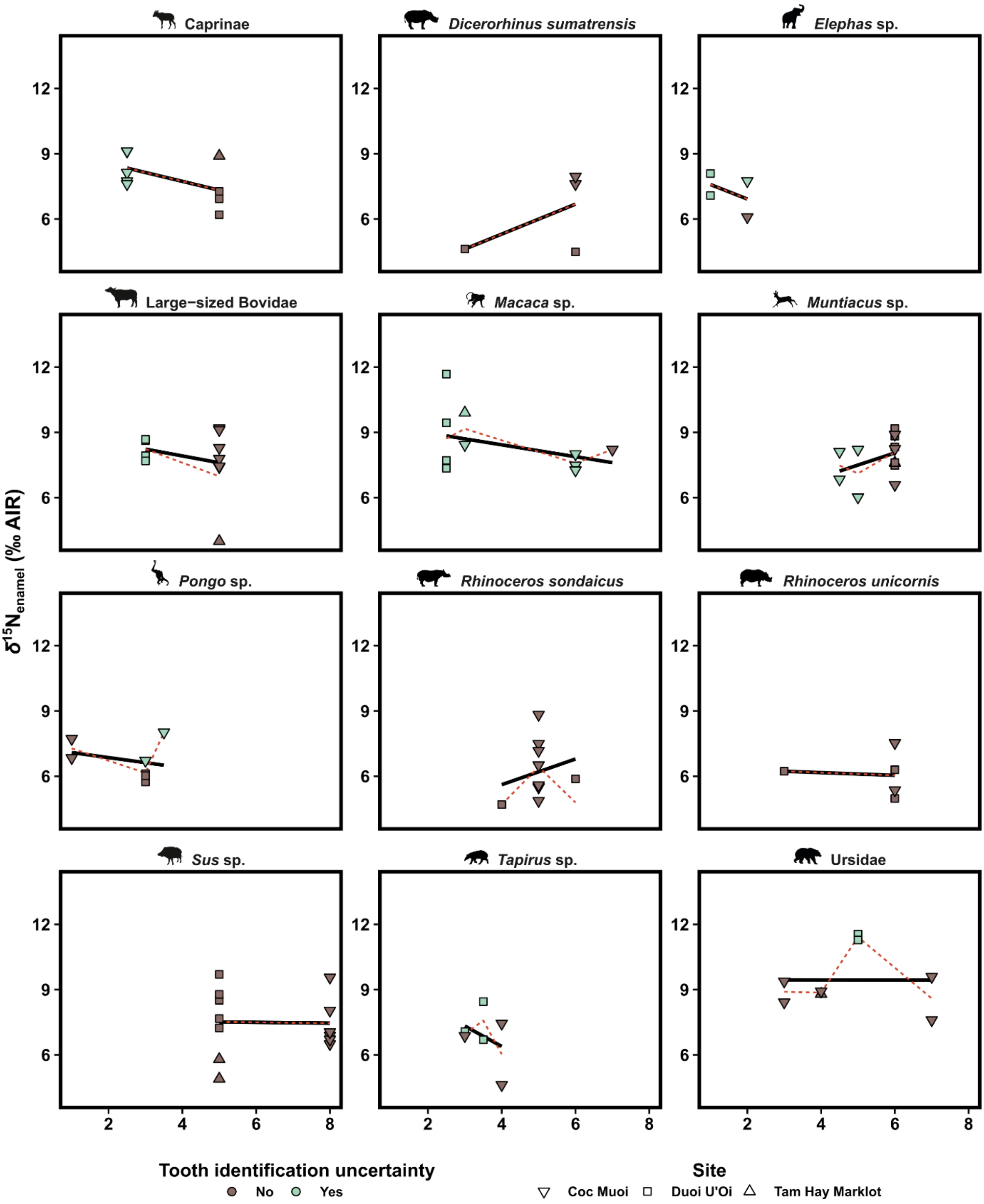

Figure S6. Relationship between δ^{15}N values and tooth eruption sequence. Relationship between $\delta^{15}N_{enamel}$ values (n = 103) and tooth formation sequence for Coc Muoi and Duoi U'Oi. Tooth formation sequence was not available for all taxa *(*108–113*)* (1 being the earliest teeth formed), and some were assigned to phylogenetically-close species (e.g., *Bos taurus* for large-sized bovids). Taxa with a single level of tooth formation were excluded. For each taxon, the black line represents the linear regression between $\delta^{15}N_{enamel}$ values and tooth formation sequence, while the red dashed line represents the moving average between $\delta^{15}N_{enamel}$ values and tooth formation sequence. Uncertainty in tooth position (e.g., "p/m", "m" rather than "m3") is expressed through points of different color: brown denotes uncertain tooth position (e.g., "p/m", "m", etc.) and teal denotes certain tooth position (e.g., "m3", "p2", etc.). The $\delta^{15}N_{enamel}$ values from Tam Hay Marklot are taken from Leichliter et al. *(*19*)*. Sites are denoted by different shapes: upside-down triangle for Coc Muoi, and square for Duoi U'Oi.

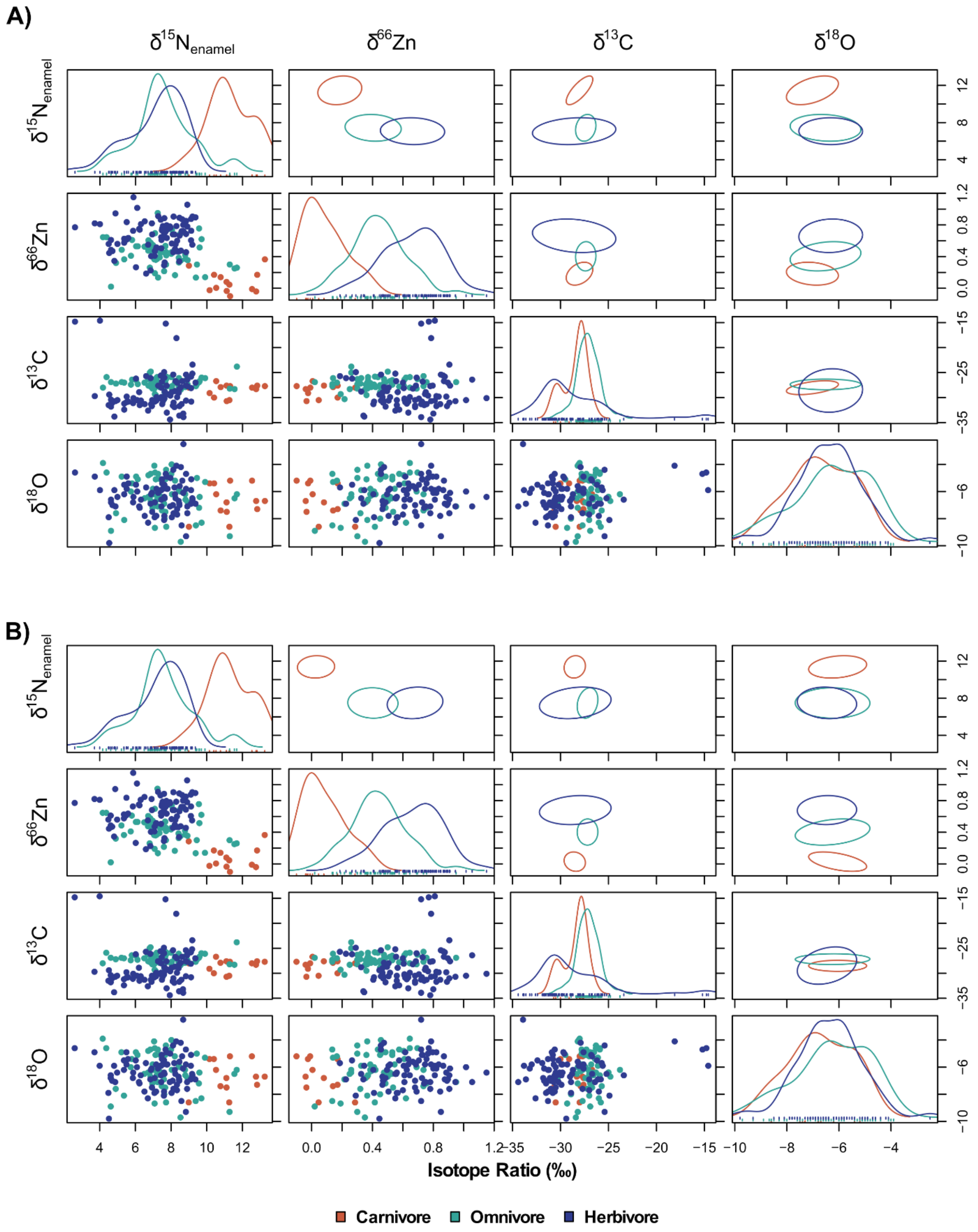

Figure S7. Two different iterations (A and B) of scatterplots, density distribution, and 40% predictive ellipses of $\delta^{15}N_{enamel}$ (‰ AIR), $\delta^{66}Zn$ (‰ JMC-Lyon), $\delta^{13}C$ (‰ VPDB), and $\delta^{18}O$ (‰ VPDB) of dietary groups from Coc Muoi and Duoi U'Oi. The predictive ellipses (i.e., a region for predicting a new observation in the population) approximate a region that contains randomly selected 40% of the population of each dietary group. As such, an inherent variability in how the ellipses are drawn exists, but all exhibit the same overall characteristics (**fig. S7A (above) and B (below), Figure 4**). When dietary groups (i.e., carnivorous, omnivorous, and herbivorous) are compared, $\delta^{15}N_{enamel}$ values cluster into high (composed primarily of carnivores) and low values (composed of herbivores and omnivores), while $\delta^{66}Zn$ values separate into low (carnivores), intermediate (omnivores), and low (herbivores). The $\delta^{66}Zn$ and $\delta^{15}N_{enamel}$ values broadly offer comparable interpretations for carnivores' and herbivores' diets in these tropical rainforest environments, and show similar relationships with $\delta^{13}C$ and $\delta^{18}O$ values. Carnivorous taxa data are represented in red, omnivorous in teal, and herbivorous in blue. The $\delta^{13}C$ values are converted to those of the food web's primary carbon sources (see Bacon et al. *(*10*)* for details), and both $\delta^{13}C$ and $\delta^{18}O$ values are taken from Bacon et al. *(*10*)*.

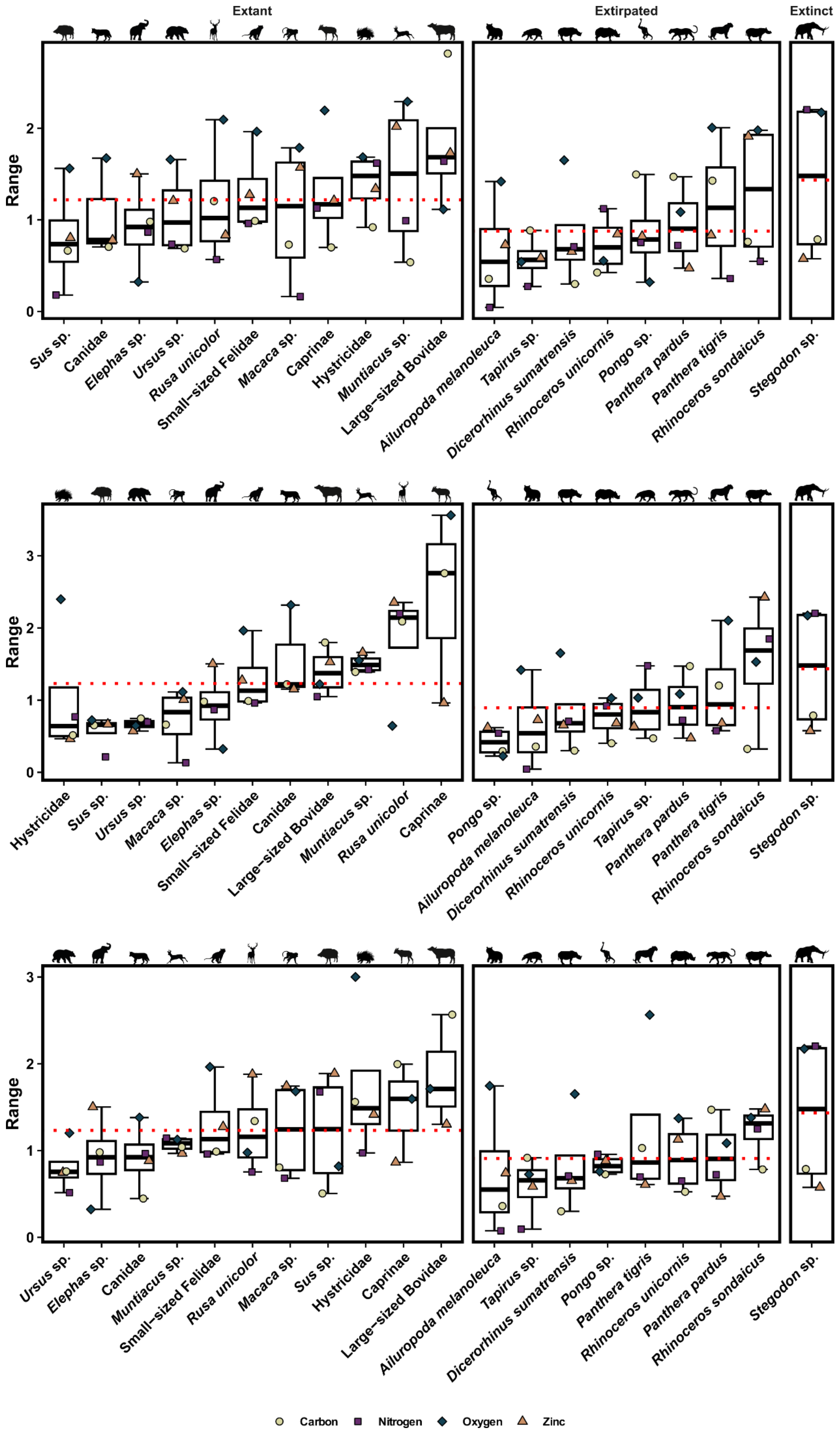

Extant
Extirpated
Extinct
Range
0
1
2
Sus sp.
Canidae
Elephas sp.
Ursus sp.
Rusa unicolor
Small-sized Felidae
Macaca sp.
Caprinae
Hystricidae
Muntiacus sp.
Large-sized Bovidae
Ailuropoda melanoleuca
Tapirus sp.
Dicerorhinus sumatrensis
Rhinoceros unicornis
Pongo sp.
Panthera pardus
Panthera tigris
Rhinoceros sondaicus
Stegodon sp.
Range
0
1
2
3
Hystricidae
Sus sp.
Ursus sp.
Macaca sp.
Elephas sp.
Small-sized Felidae
Canidae
Large-sized Bovidae
Muntiacus sp.
Rusa unicolor
Caprinae
Pongo sp.
Ailuropoda melanoleuca
Dicerorhinus sumatrensis
Rhinoceros unicornis
Tapirus sp.
Panthera pardus
Panthera tigris
Rhinoceros sondaicus
Stegodon sp.
Range
0
1
2
3
Ursus sp.
Elephas sp.
Canidae
Muntiacus sp.
Small-sized Felidae
Rusa unicolor
Macaca sp.
Sus sp.
Hystricidae
Caprinae
Large-sized Bovidae
Ailuropoda melanoleuca
Tapirus sp.
Dicerorhinus sumatrensis
Pongo sp.
Panthera tigris
Rhinoceros unicornis
Panthera pardus
Rhinoceros sondaicus
Stegodon sp.
Carbon
Nitrogen
Oxygen
Zinc

Figure S8. Average range of $\delta^{15}N_{enamel}$ (‰ AIR), $\delta^{66}Zn$ (‰ JMC-Lyon), $\delta^{13}C$ (‰ VPDB), and $\delta^{18}O$ (‰ VPDB) of extant, extirpated, and extinct taxa for the studied period after randomized resampling. Five observations were randomly selected for each taxon with $n > 5$, and this was performed three times (i.e., each graphic row). All isotopic values are standard-score transformed ((x-µ)/ σ) to allow direct comparison between the different isotope systems. The ranges (max-min) are then calculated for each site (Tam Hay Marklot, Pà Hang Mountain (comprised of Tam Pà Ling and Nam Lot), Duoi U'Oi, and Coc Muoi) and then averaged in order to alleviate the impact of outliers. Taxa present at a single site are excluded, as their range does not represent an overall one over time. The range (maximum – minimum values) of every isotopic system was calculated for each taxon at each site and across sites (i.e., absolute maximum – absolute minimum), and then averaged into a single value for each isotopic system. The ranges calculated from a single individual (i.e., $n = 1$ for any given taxon at a given site) were excluded. The $\delta^{13}C$ values were converted to those of the food web's primary carbon sources for better comparability between taxa (see Bacon et al. *(*10*)* and Bourgon et al. *(*28*)* for details), and both $\delta^{13}C$ and $\delta^{18}O$ values are taken from Bacon et al. *(*10*,* 46*)* and Bourgon et al. *(*27*,* 28*)*. The $\delta^{66}Zn$ values from Tam Hay Marklot are taken from Bourgon et al. *(*27*),* and those from Tam Pà Ling and Nam Lot from Bourgon et al. *(*28*)*. The $\delta^{15}N_{enamel}$ values from Tam Hay Marklot are taken from Leichliter et al. *(*19*)*. The $\delta^{66}Zn$ values of Coc Muoi are normalized to the average $\delta^{66}Zn$ value of all other Southeast Asian sites (i.e., Tam Hay Marklot, Nam Lot, Tam Pà Ling, and Duoi U'Oi). The boxes represent the 25th–75th percentiles, with the median represented by a bold horizontal line, and the red dotted line represents the average range for each group (extant, extirpated, and extinct).

Supplementary Material – Tables

S-EVA	Original number	Site	Taxon	Status	Teeth	Teeth formation order	Diet
34818	DU876	Duoi U'Oi	*Sus* sp.	Extant	p4 left	5	Omnivore
34819	DU890	Duoi U'Oi	*Sus* sp.	Extant	p4 left	5	Omnivore
34820	DU905	Duoi U'Oi	*Sus* sp.	Extant	p4 left	5	Omnivore
34821	DU906	Duoi U'Oi	*Sus* sp.	Extant	p4 left	5	Omnivore
34822	DU913	Duoi U'Oi	*Sus* sp.	Extant	p4 left	5	Omnivore
34823	DU546	Duoi U'Oi	*Rusa unicolor*	Extant	m3 left	5	Herbivore
34824	DU557	Duoi U'Oi	*Rusa unicolor*	Extant	m3 left	5	Herbivore
34825	DU567	Duoi U'Oi	*Rusa unicolor*	Extant	m3 left	5	Herbivore
34826	DU608	Duoi U'Oi	*Rusa unicolor*	Extant	m3 left	5	Herbivore
34827	DU990	Duoi U'Oi	*Rusa unicolor*	Extant	m3 left	5	Herbivore
34828	DU1087	Duoi U'Oi	*Rusa unicolor*	Extant	m3 left	5	Herbivore
34829	DU437	Duoi U'Oi	*Muntiacus* sp.	Extant	m3 left	6	Herbivore
34830	DU461	Duoi U'Oi	*Muntiacus* sp.	Extant	m3 left	6	Herbivore
34831	DU511	Duoi U'Oi	*Muntiacus* sp.	Extant	m3 left	6	Herbivore
34832	DU433	Duoi U'Oi	*Muntiacus* sp.	Extant	m3 left	6	Herbivore
34833	DU392	Duoi U'Oi	*Muntiacus* sp.	Extant	m3 left	6	Herbivore
34834	DU574	Duoi U'Oi	Caprinae	Extant	m3 right	5	Herbivore
34835	DU613	Duoi U'Oi	Caprinae	Extant	m3 right	5	Herbivore
34836	DU538	Duoi U'Oi	Caprinae	Extant	m3 left	5	Herbivore
34837	DU992	Duoi U'Oi	Large-sized Bovidae	Extant	m	3	Herbivore
34838	DU983	Duoi U'Oi	Large-sized Bovidae	Extant	m	3	Herbivore
34839	DU984	Duoi U'Oi	Large-sized Bovidae	Extant	m	3	Herbivore
34840	DU560	Duoi U'Oi	Large-sized Bovidae	Extant	m	3	Herbivore
34841	no N°	Duoi U'Oi	Hystricidae	Extant	i	NA	Omnivore
34842	no N°	Duoi U'Oi	Hystricidae	Extant	i	NA	Omnivore
34843	no N°	Duoi U'Oi	Hystricidae	Extant	i	NA	Omnivore
34844	no N°	Duoi U'Oi	Hystricidae	Extant	i	NA	Omnivore
34845	no N°	Duoi U'Oi	Hystricidae	Extant	i	NA	Omnivore
34846	no N°	Duoi U'Oi	Hystricidae	Extant	i	NA	Omnivore
34847	no N°	Duoi U'Oi	Hystricidae	Extant	i	NA	Omnivore
34848	DU7	Duoi U'Oi	Hystricidae	Extant	i	NA	Omnivore
34849	DU728	Duoi U'Oi	*Ursus* sp.	Extant	I3 right	5	Omnivore
34850	DU729	Duoi U'Oi	*Ursus* sp.	Extant	I3 right	5	Omnivore
34851	DU1152	Duoi U'Oi	Canidae	Extant	P4 left	4	Carnivore
34852	DU1153	Duoi U'Oi	Canidae	Extant	P4 left	4	Carnivore
34853	DU77	Duoi U'Oi	Canidae	Extant	P4 left	4	Carnivore
34854	DU68	Duoi U'Oi	*Panthera pardus*	Extirpated	p4 left	4	Carnivore
34855	DU86	Duoi U'Oi	*Panthera pardus*	Extirpated	p4 left	4	Carnivore

34856	DU707	Duoi U'Oi	*Panthera tigris*	Extirpated	m1 right	3	Carnivore
34857	DU326	Duoi U'Oi	*Macaca* sp.	Extant	i	2.5	Omnivore
34858	DU331	Duoi U'Oi	*Macaca* sp.	Extant	i	2.5	Omnivore
34859	DU339	Duoi U'Oi	*Macaca* sp.	Extant	i	2.5	Omnivore
34860	DU343	Duoi U'Oi	*Macaca* sp.	Extant	i	2.5	Omnivore
34861	DU322	Duoi U'Oi	*Macaca* sp.	Extant	i	2.5	Omnivore
34862	DU32	Duoi U'Oi	*Rhinoceros unicornis*	Extirpated	M2 right	3	Herbivore
34863	DU26	Duoi U'Oi	*Rhinoceros unicornis*	Extirpated	M3 right	6	Herbivore
34864	DU28	Duoi U'Oi	*Rhinoceros unicornis*	Extirpated	M3 right	6	Herbivore
34865	DU30	Duoi U'Oi	*Rhinoceros sondaicus*	Extirpated	M3 right	6	Herbivore
34866	DU31	Duoi U'Oi	*Rhinoceros sondaicus*	Extirpated	M3 right	6	Herbivore
34867	DU38	Duoi U'Oi	*Rhinoceros sondaicus*	Extirpated	p2 right	4	Herbivore
34868	DU27	Duoi U'Oi	*Dicerorhinus sumatrensis*	Extirpated	M3 right	6	Herbivore
34869	DU24	Duoi U'Oi	*Dicerorhinus sumatrensis*	Extirpated	M2 right	3	Herbivore
34870	DU47	Duoi U'Oi	*Tapirus* sp.	Extirpated	M1/M2 left	3	Herbivore
34871	DU43	Duoi U'Oi	*Tapirus* sp.	Extirpated	P/M left	3.5	Herbivore
34872	DU53	Duoi U'Oi	*Tapirus* sp.	Extirpated	P/M left	3.5	Herbivore
34873	DU1021	Duoi U'Oi	*Pongo* sp.	Extirpated	m3 right	3	Omnivore
34874	DU1022	Duoi U'Oi	*Pongo* sp.	Extirpated	m3 right	3	Omnivore
34875	DU1023	Duoi U'Oi	*Pongo* sp.	Extirpated	m3 right	3	Omnivore
34876	DU634	Duoi U'Oi	*Elephas* sp.	Extant	d	1	Herbivore
34877	CM169	Coc Muoi	*Muntiacus* sp.	Extant	m3 left	6	Herbivore
34878	CM307	Coc Muoi	*Muntiacus* sp.	Extant	m3 left	6	Herbivore
34879	CM357	Coc Muoi	*Muntiacus* sp.	Extant	m3 left	6	Herbivore
34880	Cm313	Coc Muoi	*Muntiacus* sp.	Extant	P2/P3 left	5	Herbivore
34881	CM175	Coc Muoi	*Muntiacus* sp.	Extant	P3/P4 left	4.5	Herbivore
34882	CM176	Coc Muoi	*Muntiacus* sp.	Extant	P3/P4 left	4.5	Herbivore
34883	CM324	Coc Muoi	*Muntiacus* sp.	Extant	P2/P3 left	5	Herbivore
34884	CM450	Coc Muoi	*Sus* sp.	Extant	m3	8	Omnivore
34885	CM676	Coc Muoi	*Sus* sp.	Extant	m3 left	8	Omnivore
34886	CM677	Coc Muoi	*Sus* sp.	Extant	m3 left	8	Omnivore
34887	CM678	Coc Muoi	*Sus* sp.	Extant	m3 left	8	Omnivore
34888	CM748	Coc Muoi	*Sus* sp.	Extant	m3 left	8	Omnivore
34889	CM746	Coc Muoi	*Sus* sp.	Extant	m3 left	8	Omnivore
34890	CM543	Coc Muoi	*Ailuropoda melanoleuca*	Extirpated	M1 left	NA	Herbivore
34891	CM544	Coc Muoi	*Ailuropoda melanoleuca*	Extirpated	M1 left	NA	Herbivore
34892	CM564	Coc Muoi	*Ailuropoda melanoleuca*	Extirpated	M1 right	NA	Herbivore
34893	CM415	Coc Muoi	*Ursus* sp.	Extant	M1 left	3	Omnivore
34894	CM518	Coc Muoi	*Ursus* sp.	Extant	m3 right	7	Omnivore

34895	CM519	Coc Muoi	*Ursus* sp.	Extant	m3 left	7	Omnivore
34896	CM521	Coc Muoi	*Ursus* sp.	Extant	M1 left	3	Omnivore
34897	CM525	Coc Muoi	*Ursus* sp.	Extant	M2 left	4	Omnivore
34898	CM508	Coc Muoi	*Pongo* sp.	Extirpated	I1 left	3.5	Omnivore
34899	CM579	Coc Muoi	*Pongo* sp.	Extirpated	M1 right	1	Omnivore
34900	CM551	Coc Muoi	*Pongo* sp.	Extirpated	m left	3	Omnivore
34901	CM553	Coc Muoi	*Pongo* sp.	Extirpated	m1 left	1	Omnivore
34902	CM509	Coc Muoi	*Macaca* sp.	Extant	c left	6	Omnivore
34903	CM577	Coc Muoi	*Macaca* sp.	Extant	c	6	Omnivore
34904	CM614	Coc Muoi	*Macaca* sp.	Extant	m1/m2 left	3	Omnivore
34905	CM618	Coc Muoi	*Macaca* sp.	Extant	M3 left	7	Omnivore
34906	CM555	Coc Muoi	*Macaca* sp.	Extant	c left	6	Omnivore
34907	CM534	Coc Muoi	*Panthera tigris*	Extirpated	P3 left	3	Carnivore
34908	CM566	Coc Muoi	*Panthera tigris*	Extirpated	P3 left	3	Carnivore
34909	CM632	Coc Muoi	*Panthera tigris*	Extirpated	m1 left	3	Carnivore
34910	CM571	Coc Muoi	Canidae	Extant	P4 right	4	Carnivore
34911	CM535	Coc Muoi	Small-sized Felidae	Extant	P4 right	3	Carnivore
34912	CM536	Coc Muoi	Small-sized Felidae	Extant	P4 right	3	Carnivore
34913	CM421	Coc Muoi	Hystricidae	Extant	i	NA	Omnivore
34914	CM472	Coc Muoi	Hystricidae	Extant	i	NA	Omnivore
34915	CM902	Coc Muoi	Hystricidae	Extant	i	NA	Omnivore
34916	CM903	Coc Muoi	Hystricidae	Extant	i	NA	Omnivore
34917	CM904	Coc Muoi	Hystricidae	Extant	i	NA	Omnivore
34918	CM910	Coc Muoi	Hystricidae	Extant	i	NA	Omnivore
34919	CM911	Coc Muoi	Hystricidae	Extant	i	NA	Omnivore
34920	CM954	Coc Muoi	Hystricidae	Extant	i	NA	Omnivore
34922	CM983	Coc Muoi	Hystricidae	Extant	i	NA	Omnivore
34923	CM984	Coc Muoi	Hystricidae	Extant	i	NA	Omnivore
34924	CM75	Coc Muoi	*Rusa unicolor*	Extant	m3 right	5	Herbivore
34925	CM244	Coc Muoi	*Rusa unicolor*	Extant	m3 left	5	Herbivore
34926	CM245	Coc Muoi	*Rusa unicolor*	Extant	m3 left	5	Herbivore
34927	CM246	Coc Muoi	*Rusa unicolor*	Extant	m3 left	5	Herbivore
34928	CM247	Coc Muoi	*Rusa unicolor*	Extant	m3 right	5	Herbivore
34929	CM249	Coc Muoi	*Rusa unicolor*	Extant	m3 right	5	Herbivore
34930	CM287	Coc Muoi	Caprinae	Extant	m1/m2 right	2.5	Herbivore
34931	CM173	Coc Muoi	Caprinae	Extant	m1/m2 left	2.5	Herbivore
34932	CM286	Coc Muoi	Caprinae	Extant	m1/m2 left	2.5	Herbivore
34933	CM147	Coc Muoi	Caprinae	Extant	m1/m2 right	2.5	Herbivore
34934	CM149	Coc Muoi	Caprinae	Extant	m1/m2 left	2.5	Herbivore
34935	CM370	Coc Muoi	Large-sized Bovidae	Extant	m3 right	5	Herbivore
34936	CM225	Coc Muoi	Large-sized Bovidae	Extant	m3 right	5	Herbivore
34937	CM226	Coc Muoi	Large-sized Bovidae	Extant	m3 right	5	Herbivore
34938	CM227	Coc Muoi	Large-sized Bovidae	Extant	m3 right	5	Herbivore

34939	CM46	Coc Muoi	Large-sized Bovidae	Extant	m3 right	5	Herbivore
34940	CM152a	Coc Muoi	Large-sized Bovidae	Extant	m3 right	5	Herbivore
34941	CM1067	Coc Muoi	*Rhinoceros sondaicus*	Extirpated	p4	5	Herbivore
34942	CM1122	Coc Muoi	*Rhinoceros sondaicus*	Extirpated	p4	5	Herbivore
34943	CM1120	Coc Muoi	*Rhinoceros sondaicus*	Extirpated	p4	5	Herbivore
34944	CM1068	Coc Muoi	*Rhinoceros sondaicus*	Extirpated	p4	5	Herbivore
34945	CM1229	Coc Muoi	*Rhinoceros sondaicus*	Extirpated	p4	5	Herbivore
34946	CM1137	Coc Muoi	*Rhinoceros sondaicus*	Extirpated	p4	5	Herbivore
34947	CM1339	Coc Muoi	*Rhinoceros sondaicus*	Extirpated	p4	5	Herbivore
34948	CM998	Coc Muoi	*Rhinoceros sondaicus*	Extirpated	p4	5	Herbivore
34949	CM1151	Coc Muoi	*Rhinoceros unicornis*	Extirpated	m3	6	Herbivore
34950	CM1278	Coc Muoi	*Rhinoceros unicornis*	Extirpated	m3	6	Herbivore
34951	CM1035	Coc Muoi	*Dicerorhinus sumatrensis*	Extirpated	m3	6	Herbivore
34952	CM1096	Coc Muoi	*Dicerorhinus sumatrensis*	Extirpated	m3	6	Herbivore
34953	CM1349	Coc Muoi	*Megatapirus augustus*	Extinct	m	NA	Herbivore
34954	CM514	Coc Muoi	*Megatapirus augustus*	Extinct	p3	NA	Herbivore
34955	CM515	Coc Muoi	*Tapirus* sp.	Extirpated	p3	3	Herbivore
34956	CM516	Coc Muoi	*Tapirus* sp.	Extirpated	p4	4	Herbivore
34957	CM1351	Coc Muoi	*Tapirus* sp.	Extirpated	p4	4	Herbivore
34958	CM726	Coc Muoi	*Stegodon* sp.	Extinct	d	1	Herbivore
34959	CM637	Coc Muoi	*Stegodon* sp.	Extinct	d	1	Herbivore
34960	CM728	Coc Muoi	*Elephas* sp.	Extant	m	2	Herbivore
34961	420	Coc Muoi	*Elephas* sp.	Extant	m1	2	Herbivore
35039	no N°	Duoi U'Oi	*Elephas* sp.	Extant	d	1	Herbivore

Table S1. Vietnam samples. Full list of Late Pleistocene fossil tooth specimens from Coc Muoi and Duoi U’Oi cave (Vietnam) used in this study, with corresponding laboratory internal S-EVA number, original sample number, broad conservation status (extant, extirpated, and extinct), tooth type and position, tooth formation order *(*108–113*)* (1 being the earliest teeth formed), and broad dietary category (carnivore, omnivore, and herbivore). Taxonomic units range from species to family levels.

S-EVA	$\delta^{13}C_{carbon\ source}$	$\delta^{13}C$	$\delta^{18}O$
34818	-27.4	-14.2	-5.1
34819	-26	-12.8	-8.3
34820	-26.9	-13.7	-7.1
34821	-28.2	-15	-6
34822	-27.9	-14.7	-6.2
34823	-30.2	-16.6	-7.1
34824	-33.2	-19.6	-6.1
34825	-31.7	-18.1	-6.1
34826	-31.7	-18.1	-7
34827	-31.9	-18.3	-5.7
34828	-31.3	-17.7	-8.5
34829	-25.4	-13.2	-9.3
34830	-27	-14.8	-7.7
34831	-26.8	-14.6	-5.1
34832	-26	-13.8	-7.8
34833	-25.2	-13	-6.5
34834	-28.9	-15.8	-7.1
34835	-30.5	-17.4	-7.5
34836	-27.6	-14.5	-6.1
34837	-34.4	-19.8	-7.1
34838	-26.7	-12.1	-5.2
34839	-15.2	-0.6	-4.7
34840	-33.9	-19.3	-2.5
34841	-28.9	-16.7	-8
34842	-24.8	-12.6	-6.7
34843	-28.4	-16.26	-9.71
34844	-25	-12.8	-6.39
34845	-29.3	-17.08	-8.68
34846	-25.9	-13.7	-6.6
34847	-29.5	-17.3	-5.1
34848	-26.9	-14.7	-8.9
34849	-28.3	-15	-6.5
34850	-28.2	-14.9	-9.3
34851	-27.6	-14.2	-7.3
34852	-28.3	-14.9	-6.7
34853	-27.9	-14.5	-7.4
34854	-28	-14.2	-5.2
34855	-27.7	-13.9	-5.2
34856	-30	-15.5	-5.4
34857	-23.8	-11.9	-6.2
34858	-27.5	-15.6	-5.1
34859	-26.3	-14.4	-4.4
34860	-27.3	-15.4	-5
34861	-27.9	-16	-6.3

34862	-31.5	-17.1	-7.2
34863	-30.5	-16.1	-6.4
34864	-30.6	-16.2	-6.6
34865	-30.1	-15.7	-6.4
34866	-29.7	-15.3	-4.9
34867	-31	-16.6	-7
34868	-31.5	-17.5	-7.9
34869	-30.4	-16.4	-6.6
34870	-30.8	-17.3	-7.9
34871	-33.6	-20.1	-6.9
34872	-32.6	-19.1	-8.3
34873	-27.4	-14.6	-6
34874	-27.3	-14.5	-5.1
34875	-26.5	-14.3	-4.9
34876	-31.6	-16.9	-7
34878	-26.7	-14.5	-6.5
34879	-25.7	-13.5	-5.7
34880	-26.2	-14	-5.5
34881	-26.1	-13.9	-6.9
34882	-25.4	-13.2	-4.4
34883	-28.7	-16.5	-6.2
34884	-27.7	-14.5	-6.2
34885	-27.1	-13.9	-6.9
34886	-27	-13.8	-6.5
34887	-26.3	-13.1	-6.4
34888	-26.9	-13.7	-7.2
34889	-27.1	-13.9	-6.2
34890	-27.9	-17.4	-4.2
34891	-28.2	-17.7	-5.6
34893	-27.8	-14.5	-5.8
34894	-30.3	-17	-6.9
34896	-27.7	-14.4	-8.5
34897	-27.4	-14.1	-6.8
34898	-28.1	-15.3	-4
34899	-27.8	-15	-4.7
34900	-27.6	-14.8	-4.8
34901	-27.8	-15	-5
34902	-25.9	-14	-4.7
34903	-25.7	-13.8	-7.4
34904	-26.1	-14.2	-5.9
34905	-27	-15.1	-5.2
34906	-27.3	-15.4	-4.7
34907	-30.7	-16.2	-7.5
34908	-29.1	-14.6	-5.4
34909	-30.5	-16	-8.6
34910	-27.7	-14.3	-6.7

34911	-28	-14.5	-8.6
34912	-26.7	-13.2	-6.2
34913	-27	-14.8	-8.1
34914	-28	-15.8	-3.9
34915	-28.2	-16	-7.2
34916	-26.7	-14.5	-7.5
34917	-28.2	-16	-4.1
34918	-25.8	-13.6	-8.6
34919	-25.9	-13.7	-4.6
34920	-26.9	-14.7	-4.9
34922	-26.8	-14.6	-5.8
34923	-26.3	-14.1	-4.6
34924	-31.1	-17.5	-6.8
34925	-27	-13.4	-6.9
34926	-28.8	-15.2	-6.5
34927	-27.6	-14	-5.8
34928	-30.6	-17	-7.8
34929	-29.9	-16.3	-7.3
34930	-28.1	-15	-5.9
34931	-30.8	-17.7	-5.1
34932	-31	-17.9	-7.6
34933	-27.6	-14.5	-4.9
34934	-30.2	-17.1	-5.5
34935	-29.3	-14.8	-5.8
34936	-23.4	-8.9	-6.6
34937	-25.6	-11.1	-5.9
34938	-25.8	-11.3	-6.9
34939	-18.1	-3.6	-4.1
34940	-28	-13.4	-5.9
34941	-31.3	-17	-5.4
34942	-30.9	-16.6	-4.9
34943	-29.9	-15.6	-5.8
34944	-29.8	-15.5	-5.8
34945	-30.6	-16.3	-4.7
34946	-31.3	-17	-7.8
34947	-30.8	-16.5	-5.4
34948	-30.7	-16.4	-7.1
34949	-30.7	-16.3	-5.8
34950	-31.2	-16.8	-6.2
34951	-29.8	-15.8	-6.4
34952	-29.6	-15.6	-5
34953	-33.8	-20.1	-5.9
34954	-32.4	-18.7	-5.5
34955	-31.3	-17.8	-6.2
34956	-31.8	-18.3	-6.8
34957	-29.9	-16.4	-6.6

34958	-28.9	-14.2	-7.9
34959	-29.4	-14.7	-9.8
34960	-33	-18.3	-5.8
34961	-30.5	-15.8	-6
35039	-30.6	-15.9	-6.8

Table S2. Carbon and oxygen isotope data from Duoi U'Oi and Coc Moi. Corresponding laboratory number, $\delta^{13}C_{carbon\ source}$, $\delta^{13}C$, and $\delta^{18}O$ values (‰ VPDB) for each sample from Bacon et al. (*10*).

S-EVA	Original number	Site	Taxon	Status	Teeth	Teeth formation order	Diet
34438	NL-8	Nam Lot	Caprinae	Extant	m3 right	5	Herbivore
34439	NL-9	Nam Lot	Caprinae	Extant	m3 right	5	Herbivore
34441	NL-19	Nam Lot	*Rusa unicolor*	Extant	m1/m2 left	2.5	Herbivore
34442	NL-29	Nam Lot	*Rusa unicolor*	Extant	p3 right	4	Herbivore
34443	NL-22	Nam Lot	*Rusa unicolor*	Extant	p3 left	4	Herbivore
34444	NL-24	Nam Lot	*Rusa unicolor*	Extant	m1/m2 left	2.5	Herbivore
34445	NL-63-1	Nam Lot	*Muntiacus* sp.	Extant	M. right	3.5	Herbivore
34446	NL-65-1	Nam Lot	*Muntiacus* sp.	Extant	M. right	3.5	Herbivore
34447	NL-69	Nam Lot	*Muntiacus* sp.	Extant	M. left	3.5	Herbivore
34448	NL-116	Nam Lot	Large-sized Bovidae	Extant	m3 left	5	Herbivore
34449	NL-117	Nam Lot	Large-sized Bovidae	Extant	m.	3	Herbivore
34450	NL-125	Nam Lot	Large-sized Bovidae	Extant	M.	3	Herbivore
34451	NL-161	Nam Lot	Rhinocerotidae indet.	Extirpated	cheek teeth inf. left	4	Herbivore
34452	NL-162	Nam Lot	Rhinocerotidae indet.	Extirpated	d left	1	Herbivore
34453	NL-254-1-1	Nam Lot	Rhinocerotidae indet.	Extirpated	d. left	1	Herbivore
34454	NL-256-1	Nam Lot	Rhinocerotidae indet.	Extirpated	d.	1	Herbivore
34455	NL-139	Nam Lot	Large-sized Bovidae	Extant	p4 right	4	Herbivore
34456	NL-143	Nam Lot	Large-sized Bovidae	Extant	p4 left	4	Herbivore
34457	NL-186	Nam Lot	*Ailuropoda melanoleuca*	Extirpated	m1 right	NA	Herbivore
34458	NL-277	Nam Lot	*Ailuropoda melanoleuca*	Extirpated	M2 left	NA	Herbivore
34459	NL-162	Nam Lot	*Sus* sp.	Extant	m1 right	2	Omnivore
34460	NL-208	Nam Lot	*Sus* sp.	Extant	p4 right	5	Omnivore
34461	NL-216	Nam Lot	*Sus* sp.	Extant	p4 right	5	Omnivore
34462	NL-218	Nam Lot	*Sus* sp.	Extant	p4 right	5	Omnivore
34463	NL-SS-1	Nam Lot	*Sus* sp.	Extant	frag.	5	Omnivore
34465	NL-258	Nam Lot	*Tapirus* sp.	Extirpated	m. left	3.5	Herbivore
34466	NL-259	Nam Lot	*Tapirus* sp.	Extirpated	i1 left	2	Herbivore
34467	NL-260	Nam Lot	*Tapirus* sp.	Extirpated	I1 left	2	Herbivore
34468	NL-286	Nam Lot	Canidae	Extant	p.	3.75	Carnivore
34469	NL-368	Nam Lot	Canidae	Extant	p3 left	4	Carnivore
34470	NL-269	Nam Lot	*Ursus* sp.	Extant	m3 right	7	Omnivore
34471	NL-271	Nam Lot	*Ursus* sp.	Extant	m3 right	7	Omnivore
34472	NL-275	Nam Lot	*Ursus* sp.	Extant	P4 left	3	Omnivore
34476	NL-HS	Nam Lot	*Pongo* sp.	Extirpated	i	3.5	Omnivore
34478	NL-297	Nam Lot	*Macaca* sp.	Extant	i	2.5	Omnivore
34479	NL-314	Nam Lot	*Macaca* sp.	Extant	m3 right	7	Omnivore

34480	NL-323	Nam Lot	*Macaca* sp.	Extant	m3 right	7	Omnivore
34481	NL-357	Nam Lot	*Macaca* sp.	Extant	i	2.5	Omnivore
34482	NL-362	Nam Lot	*Elephas* sp.	Extant	d	1	Herbivore
34483	NL-365	Nam Lot	*Stegodon* sp.	Extinct	d	1	Herbivore
34484	NL-367	Nam Lot	*Stegodon* sp.	Extinct	d	1	Herbivore
34485	NL-369	Nam Lot	Hystricidae	Extant	p./m.	NA	Omnivore
34486	NL-385	Nam Lot	Hystricidae	Extant	i	NA	Omnivore
34487	NL-392	Nam Lot	Hystricidae	Extant	p./m.	NA	Omnivore
34488	NL-397	Nam Lot	Hystricidae	Extant	p./m.	NA	Omnivore
34489	MI-20	Tam Hay Marklot	Caprinae	Extant	m3 right	5	Herbivore
34490	MI-21	Tam Hay Marklot	Caprinae	Extant	m3 right	5	Herbivore
34491	MI-22	Tam Hay Marklot	Caprinae	Extant	m3 right	5	Herbivore
34492	MI-23	Tam Hay Marklot	Caprinae	Extant	m3 right	5	Herbivore
34493	MI-24	Tam Hay Marklot	Caprinae	Extant	m3 right	5	Herbivore
34494	MI-25	Tam Hay Marklot	Caprinae	Extant	m3 left	5	Herbivore
34495	MI-26	Tam Hay Marklot	Caprinae	Extant	m3 left	5	Herbivore
34496	MI-27	Tam Hay Marklot	Caprinae	Extant	m3 left	5	Herbivore
34497	MI-28	Tam Hay Marklot	Caprinae	Extant	m3 left	5	Herbivore
34498	MI-103	Tam Hay Marklot	*Ursus* sp.	Extant	M2 left	4	Omnivore
34499	MI-121	Tam Hay Marklot	*Ursus* sp.	Extant	M2 left	4	Omnivore
34500	MI-117	Tam Hay Marklot	*Ursus* sp.	Extant	M2 right	4	Omnivore
34501	MI-119	Tam Hay Marklot	*Ursus* sp.	Extant	M2 left	4	Omnivore
34502	MI-122	Tam Hay Marklot	*Ursus* sp.	Extant	M2 left	4	Omnivore
34503	MI-134	Tam Hay Marklot	*Panthera pardus*	Extirpated	P3 right	3	Carnivore
34504	MI-135	Tam Hay Marklot	*Panthera pardus*	Extirpated	p4 left	4	Carnivore
34505	MI-136	Tam Hay Marklot	*Panthera pardus*	Extirpated	P4 left	4	Carnivore
34506	MI-166	Tam Hay Marklot	*Rusa unicolor*	Extant	m3 left	5	Herbivore
34507	MI-180	Tam Hay Marklot	*Rusa unicolor*	Extant	m3 left	5	Herbivore
34508	MI-185	Tam Hay Marklot	*Rusa unicolor*	Extant	m3 left	5	Herbivore
34509	MI-187	Tam Hay Marklot	*Rusa unicolor*	Extant	m3 left	5	Herbivore
34510	MI-191	Tam Hay Marklot	*Rusa unicolor*	Extant	m3 left	5	Herbivore
34515	MI-627	Tam Hay Marklot	*Muntiacus* sp.	Extant	m3 right	6	Herbivore

34516	MI-628	Tam Hay Marklot	*Muntiacus* sp.	Extant	m3 right	6	Herbivore
34517	MI-629	Tam Hay Marklot	*Muntiacus* sp.	Extant	m3 right	6	Herbivore
34518	MI-630	Tam Hay Marklot	*Muntiacus* sp.	Extant	m3 right	6	Herbivore
34519	MI-631	Tam Hay Marklot	*Muntiacus* sp.	Extant	m3 right	6	Herbivore
34520	MI-650	Tam Hay Marklot	Large-sized Bovidae	Extant	p2 (p3?) right	5	Herbivore
34521	MI-651	Tam Hay Marklot	Large-sized Bovidae	Extant	p2 (p3?) right	5	Herbivore
34522	MI-652	Tam Hay Marklot	Large-sized Bovidae	Extant	p2 (p3?) left	5	Herbivore
34523	MI-653	Tam Hay Marklot	Large-sized Bovidae	Extant	p2 (p3?) left	5	Herbivore
34524	MI-654	Tam Hay Marklot	Large-sized Bovidae	Extant	p2 (p3?) left	5	Herbivore
34525	MI-655	Tam Hay Marklot	Large-sized Bovidae	Extant	p2 right	5	Herbivore
34526	MI-656	Tam Hay Marklot	Large-sized Bovidae	Extant	p2 right	5	Herbivore
34527	MI-657	Tam Hay Marklot	Large-sized Bovidae	Extant	p2 right	5	Herbivore
34528	MI-658	Tam Hay Marklot	Large-sized Bovidae	Extant	p2 left	5	Herbivore
34529	MI-659	Tam Hay Marklot	Large-sized Bovidae	Extant	p2 left	5	Herbivore
34530	MI-130	Tam Hay Marklot	*Panthera tigris*	Extirpated	p4 right	4	Carnivore
34531	MI-693	Tam Hay Marklot	*Panthera tigris*	Extirpated	P4 left	4	Carnivore
34532	MI-694	Tam Hay Marklot	*Panthera tigris*	Extirpated	P4 left	4	Carnivore
34533	MI-662	Tam Hay Marklot	*Sus* sp.	Extant	p4 left	5	Omnivore
34534	MI-663	Tam Hay Marklot	*Sus* sp.	Extant	p4 left	5	Omnivore
34535	MI-664	Tam Hay Marklot	*Sus* sp.	Extant	p4 left	5	Omnivore
34536	MI-665	Tam Hay Marklot	*Sus* sp.	Extant	p4 left	5	Omnivore
34537	MI-666	Tam Hay Marklot	*Sus* sp.	Extant	p4 left	5	Omnivore
34538	MI-667	Tam Hay Marklot	*Sus* sp.	Extant	p4 left	5	Omnivore
34539	MI-131	Tam Hay Marklot	Canidae	Extant	m1 right	3	Carnivore
34540	MI-681	Tam Hay Marklot	Canidae	Extant	P3 right	4	Carnivore
34541	MI-683	Tam Hay Marklot	*Pongo* sp.	Extirpated	C	5	Omnivore
34542	MI-685	Tam Hay Marklot	*Pongo* sp.	Extirpated	M left	3	Omnivore
34543	MI-682	Tam Hay Marklot	Canidae	Extant	M1 left	3	Carnivore

34544	MI-684	Tam Hay Marklot	*Ailuropoda melanoleuca*	Extirpated	m2 right	NA	Herbivore
34545	MI-691	Tam Hay Marklot	*Tapirus* sp.	Extirpated	c left	3	Herbivore
34546	MI-692	Tam Hay Marklot	*Tapirus* sp.	Extirpated	cheek teeth	3.5	Herbivore
34547	MI-695	Tam Hay Marklot	*Macaca* sp.	Extant	m1/m2 left	3	Omnivore
34548	MI-696	Tam Hay Marklot	*Macaca* sp.	Extant	m1/m2 left	3	Omnivore
34549	MI-697	Tam Hay Marklot	*Macaca* sp.	Extant	pm right	4	Omnivore
34550	MI-698	Tam Hay Marklot	*Macaca* sp.	Extant	m1/m2 left	3	Omnivore
34551	MI-699	Tam Hay Marklot	*Macaca* sp.	Extant	pm right	4	Omnivore
34552	MI-700	Tam Hay Marklot	Hystricidae	Extant	i	NA	Omnivore
34553	MI-701	Tam Hay Marklot	Hystricidae	Extant	i	NA	Omnivore
34554	MI-702	Tam Hay Marklot	Hystricidae	Extant	i	NA	Omnivore
34555	MI-703	Tam Hay Marklot	Hystricidae	Extant	i	NA	Omnivore
34556	MI-686	Tam Hay Marklot	*Rhinoceros sondaicus*	Extirpated	m3 left	6	Herbivore
34557	MI-687	Tam Hay Marklot	*Rhinoceros sondaicus*	Extirpated	m3 left	6	Herbivore
34558	MI-688	Tam Hay Marklot	*Rhinoceros sondaicus*	Extirpated	m2 left	3	Herbivore
34559	MI-689	Tam Hay Marklot	*Rhinoceros sondaicus*	Extirpated	d3 left	1	Herbivore
34560	MI-690	Tam Hay Marklot	*Rhinoceros sondaicus*	Extirpated	d3 right	1	Herbivore
35416	M33	Tam Pà Ling	Caprinae	Extant	m1/m2 left	2.5	Herbivore
35417	M31	Tam Pà Ling	Caprinae	Extant	M left	3	Herbivore
35418	M35	Tam Pà Ling	Caprinae	Extant	m1/m2 right	2.5	Herbivore
35419	M31	Tam Pà Ling	Caprinae	Extant	m1/m2 left	2.5	Herbivore
35420	K32	Tam Pà Ling	Caprinae	Extant	m1/m2 right	2.5	Herbivore
35421	K32	Tam Pà Ling	Caprinae	Extant	M3 left	5	Herbivore
35422	M35	Tam Pà Ling	Large-sized Bovidae	Extant	D3 left	1	Herbivore
35423	L33	Tam Pà Ling	Caprinae	Extant	d2 left	1	Herbivore
35425	K32	Tam Pà Ling	*Macaca* sp.	Extant	M left	4	Omnivore
35426	L36	Tam Pà Ling	Caprinae	Extant	d3 left	1	Herbivore
35427	M31	Tam Pà Ling	Rhinocerotidae	Extirpated	d2 left	1	Herbivore

35428	M31	Tam Pà Ling	Rhinocerotidae	Extirpated	D3 left	1	Herbivore
35429	K33	Tam Pà Ling	Caprinae	Extant	m1/m2	2.5	Herbivore
35945	NLII-5	Nam Lot	Small-sized Felidae	Extant	P3 left	3	Carnivore
35946	H33	Tam Pà Ling	Caprinae	Extant	m3 left	5	Herbivore
35947	M32	Tam Pà Ling	Caprinae	Extant	d3 right	1	Herbivore
35948	M32	Tam Pà Ling	Hystricidae	Extant	i	NA	Omnivore
35949	M29-30	Tam Pà Ling	Hystricidae	Extant	i	NA	Omnivore
35950	K33	Tam Pà Ling	Hystricidae	Extant	i	NA	Omnivore
35951	L32	Tam Pà Ling	*Rusa unicolor*	Extant	m3 (frag)	5	Herbivore
35952	K33	Tam Pà Ling	Caprinae	Extant	i	2	Herbivore
35954	L32	Tam Pà Ling	*Muntiacus* sp.	Extant	D3 right	1	Herbivore
35955	J31	Tam Pà Ling	Caprinae	Extant	D2 right	1	Herbivore
35957	O32	Tam Pà Ling	Large-sized Felidae	Extant	I3 left	2	Carnivore
35958	K35	Tam Pà Ling	*Rhinoceros sondaicus*	Extirpated	M1 left	2	Herbivore

Table S3. Laos samples. Full list of Late Pleistocene fossil tooth specimens from Tam Hay Marklot, Nam Lot, and Tam Pà Ling used in this study from Bacon et al. *(*10*,* 46*)* and Bourgon et al. *(*27*,* 28*)*, with corresponding laboratory internal S-EVA number, original sample number, broad conservation status (extant, extirpated, and extinct), tooth type and position, tooth formation order *(*108–113*)* (1 being the earliest teeth formed), and broad dietary category (carnivore, omnivore, and herbivore). Taxonomic units range from species to family levels.

S-EVA	$\delta^{13}C_{carbon\ source}$	$\delta^{13}C$	$\delta^{18}O$	$\delta^{66}Zn$	$\delta^{15}N_{enamel}$
34438	-27.7	-14.6	-4.1	0.82	
34439	-26.1	-13	-3.3	0.88	
34441	-21.1	-7.5	-5.8	0.69	
34442	-27.7	-14.1	-6.7	0.88	
34443	-14.5	-0.9	-5.5	0.83	
34444	-16.3	-2.7	-6.1	0.53	
34445	-25.6	-13.4	-6.4	0.40	
34446	-26	-13.8	-7.3	0.27	
34447	-26.1	-13.9	-3.3	0.5	
34448	-24.4	-9.8	-5.9	0.67	
34449	-14.3	0.3	-4.9	0.39	
34450	-28.1	-13.5	-4.7	0.32	
34451	-27.6	-13.3	-5.9	0.74	
34452	-27.4	-13.1	-2.5	1.02	
34453	-27	-12.7	-6.7	0.66	
34454.1	-29.4	-15.1	-6.6	0.75	
34455	-19.4	-4.7	-6.5	0.72	
34456	-24.2	-9.5	-4.4	0.67	
34457	-25	-14.5	-6.2	0.61	
34458	-25.4	-14.9	-4.2	0.47	
34459	-26.9	-13.7	-5.7	0.54	
34460	-25.6	-12.4	-6	0.51	
34461	-26	-12.8	-5.3	0.54	
34462	-27.3	-14.1	-6.8	0.63	
34463	-26.3	-13.1	-6	0.59	
34465	-31	-17.5	-6.4	0.67	
34466	-28.4	-14.9	-6.8	0.59	
34467	-28.8	-15.3	-5	0.45	
34468	-26.7	-13.3	-6.4	0.14	
34469	-26.4	-13	-3	0.15	
34470	-28.6	-15.3	-9	0.17	
34471	-27.4	-14.1	-8.3	0.38	
34472	-25.9	-12.6	-6.5	0.58	
34474	-27.7	-13.7	-6.4	0.52	
34475	-27.6	-13.6	-6.1	0.57	
34476	-27.3	-14.5	-3.3	0.8	
34478	-26	-14.1	-4.8	0.14	
34479	-23.4	-11.5	-3.7	0.12	
34480	-26.7	-14.8	-6.1	0.05	
34481	-26.5	-14.6	-3.6	0.18	
34482	-30.8	-16.1	-6.2	0.59	
34483	-32.9	-18.2	-4.3	0.41	
34484	-30.1	-15.4	-6.5	0.56	
34485	-25.8	-13.6	-6.5	0.43	

34486	-24.3	-12.1	-5.7	0.46	
34487	-26.3	-14.1	-5.2	0.25	
34488	-25.5	-13.3	-5.7	0.29	
34489	-26.5	-13.4	-2.2	0.52	
34490	-27.8	-14.7	-5.2	0.8	
34491	-28.3	-15.2	-5.2	0.66	
34492	-26.6	-13.5	-7	0.61	
34493	-27.7	-14.6	-7.6	0.78	8.9
34494	-14.6	-2.4	0.2	0.86	
34495	-16.1	-3.9	-2.5	0.9	
34496	-15.9	-3.7	-1.8	1.00	
34497	-14.7	-2.5	-1.6	0.76	
34498	-27.3	-14.7	-3.9	0.67	
34499	-27.5	-14.9	-5.5	0.24	
34500	-28.7	-15.4	-7.4	0.43	
34501	-26.6	-13.3	-6.6	0.37	8.8
34502	-27.7	-14.4	-6.3	0.39	
34503	-21.7	-7.9	-7.3	-0.01	
34504	-17.8	-4	-7.3	0.01	
34505	-27.6	-13.8	-6.8	0.08	11.1
34506	-21.1	-7.5	-5.7	0.47	
34507	-18.9	-5.3	-4.4	0.5	
34508	-19.6	-6	-4.9	0.47	
34509	-17	-3.4	-4.9	0.78	
34510	-21.5	-7.9	-6.1	0.61	
34511	-10.8	2.4	-5.3	0.55	
34512	-11.4	1.8	-3.1	0.71	
34513	-13.3	-0.8	-5.8	0.72	
34514	-13.1	-0.6	-5.3	0.62	
34515	-26	-13.8	-7.4	0.6	
34516	-27	-14.8	-8.1	0.47	
34517	-24.9	-12.7	-8.1	0.29	7.6
34518	-26.6	-14.4	-5	0.45	
34519	-26.7	-14.5	-7.5	0.65	
34520	-25	-10.3	-6	0.59	
34521	-18.7	-4	-6.6	0.5	
34522	-13.7	1	-4.7	0.63	
34523	-25.6	-10.9	-6.3	0.55	
34524	-14.6	0.1	-5.9	0.81	4.0
34525	-17	-2.5	-4.2	0.75	
34526	-28.9	-14.4	-6.1	0.95	
34527	-14.8	-0.3	-4.6	0.77	2.6
34528	-24.5	-10	-7.6	1.03	
34529	-27.6	-13.1	-5.9	0.98	
34530	-18.8	-4.3	-3.2	-0.01	
34531	-21.4	-6.9	-3.5	0.05	

34532	-24.5	-10	-6.8	0.16	
34533	-20.8	-7.6	-7.5	0.7	
34534	-21.8	-8.6	-10	0.5	
34535	-19.2	-6	-5.9	0.72	
34536	-26.7	-13.5	-5.8	0.61	
34537	-27.2	-14	-7.6	0.32	4.9
34538	-26.4	-13.2	-5.4	0.61	5.8
34539	-29.4	-16	-7.3	0.02	
34540	-24.8	-11.4	-3.2	0.2	
34541	-27.6	-14.8	-4.1	0.6	
34542	-26.3	-13.5	-4.6	0.38	
34543	-26.6	-13.2	-6	0.34	
34544	-27.2	-16.7	-6.3	0.53	
34545	-29	-15.5	-5.9	0.5	
34546	-24.8	-11.3	-7.9	0.39	
34547	-25.8	-13.9	-5.3	0.22	
34548	-26.1	-14.2	-5.3	0.14	9.9
34549	-27	-15.1	-5.6	0.35	
34550	-25.2	-13.3	-4.7	0.3	
34551	-24.8	-12.9	-4.6	0.32	
34552	-23.7	-11.5	-7.6	0.42	
34553	-23.7	-11.5	-5.5	0.29	
34554	-20.1	-7.9	-5.2	0.43	
34555	-23.8	-11.6	-8.5	-0.04	
34556	-30.5	-16.2	-6.5	0.52	
34557	-27.6	-13.3	-5.5	0.86	
34558	-29.9	-15.6	-6.3	0.9	
34559	-29.6	-15.3	-7.1	0.79	
34560	-28.9	-14.6	-7.5	1.03	
35416	-26	-12.9	-5.8	0.76	
35417	-26.7	-13.6	-6.2	1.00	
35418	-17.2	-5	-3.1	1.03	
35419	-28.5	-16.3	-2.1	0.8	
35420	-14.5	-2.3	-3.3	1.07	
35421	-26.9	-14.7	-5.8	0.54	
35422	-29.6	-15	-2.7	0.52	
35423	-18.6	-5.5	-0.6	0.93	
35425	-26.2	-14.3	-5	0.07	
35426	-26.8	-14.6	-3.3	0.76	
35427	-30.2	-15.8	-9.1	0.99	
35428	-29.8	-15.8	-8.9	0.80	
35429	-27.1	-14.03	-8.03	0.86	
35941	-28.3	-14.3	-6.6	0.31	
35942	-26.5	-12.5	-6.8	0.24	
35943	-29.1	-15.1	-6.2	0.47	
35944	-25.8	-11.8	-7.2	0.43	

35945	-28.5	-15	-6.6	-0.11	
35946	-27.1	-13.97	-6.82	0.99	
35947	-27.8	-14.65	-6.64	0.66	
35948	-23.2	-10.99	-6.68	0.69	
35949	-22.3	-10.11	-9.67	0.35	
35950	-24.6	-12.37	-5.76	0.54	
35951	-27.5	-13.9	-6.69	0.76	
35952	-27	-13.89	-4.45	0.83	
35954	-23.6	-11.41	-6.88	0.89	
35955	-13.5	-1.21	-6.83	1.01	
35957	-28.6	-14.33	-6.77	-0.04	
35958	-30	-15.71	-3.86	0.99	

Table S4. Carbon, oxygen, zinc, and nitrogen isotope data from Tam Hay Marklot, Nam Lot, and Tam Pà Ling, northern Laos. The $\delta^{13}C_{\text{carbon source}}$, $\delta^{13}C$, and $\delta^{18}O$ values are taken from Bacon et al. *(*10*,* 46*)* and Bourgon et al. (27, 28). The $\delta^{66}Zn$ values from Tam Hay Marklot are taken from Bourgon et al. (27), and those from Tam Pà Ling and Nam Lot from Bourgon et al. (28). The $\delta^{15}N_{\text{enamel}}$ values from Tam Hay Marklot are taken from Leichliter et al. (19). Corresponding laboratory internal S-EVA number is associated with each sample as per their initial publication.

S-EVA	δ^{66}Zn	δ^{66}Zn SD	δ^{66}Zn normalized	δ^{67}Zn	δ^{68}Zn	[Zn] µg/g	Zn µg
34818	0.53	0.04		1.06	1.12	54	482
34819	0.62	0.04		1.21	1.32	38	414
34820	0.95	0.03		1.67	1.95	39	373
34821	0.69	0.03		1.3	1.44	36	352
34822	0.42	na		1.29	1.14	27	332
34823	0.61	na		1.53	1.49	31	368
34824	0.8	0.02		1.41	1.65	66	675
34825	0.82	0.00		1.42	1.67	141	734
34826	0.89	0.00		1.46	1.81	128	1223
34827	0.68	0.03		1.39	1.53	37	345
34828	0.72	na		1.7	1.76	26	341
34829	0.85	0.02		1.61	1.86	39	386
34830	0.6	0.02		1.18	1.31	38	363
34831	0.73	0.02		1.42	1.57	23	228
34832	0.69	0.03		1.43	1.46	19	190
34833	0.55	0.03		1.19	1.22	25	263
34834	0.75	0.01		1.31	1.55	71	679
34835	0.68	0.02		1.19	1.38	69	637
34836	0.91	0.01		1.55	1.85	78	759
34837	0.78	0.00		1.4	1.61	59	568
34838	0.87	na		1.47	1.75	56	411
34839	0.72	0.01		1.58	1.62	42	651
34840	0.72	na		1.53	1.59	41	390
34841	0.45	na		1.16	1.09	43	502
34842	0.5	0.02		1.14	1.12	30	324
34843	0.35	0.25		1.16	0.98	23	249
34844	0.26	0.01		0.96	0.69	22	224
34845	0.19	0.14		1.01	0.66	21	232
34846	0.37	0.00		0.99	0.86	21	225
34847	0.36	0.01		1.08	0.86	17	190
34848	0.02	0.24		0.15	0.07	12	265
34849	0.25	0.01		0.76	0.63	33	341
34850	0.3	0.01		0.73	0.66	43	678
34851	0.17	0.02		0.74	0.48	22	240
34852	-0.04	0.00		0.17	-0.01	64	447
34853	0.14	0.00		0.62	0.4	26	269
34854	-0.01	0.01		0.2	0.04	77	800
34855	-0.1	0.00		0.19	-0.11	62	659
34856	0.17	0.01		0.51	0.39	74	856
34857	0.26	0.01		0.61	0.57	122	1296
34858	0.45	0.00		0.82	0.94	162	1800
34859	0.3	0.01		0.68	0.64	96	1013
34860	0.3	0.01		0.61	0.66	101	1118
34861	0.39	0.01		0.82	0.8	105	1107

34862	0.74	na		1.46	1.61	8	136
34863	0.56	na		0.8	1.15	15	85
34864	0.81	0.01		1.61	1.71	32	340
34865	1.15	0.01		2.01	2.37	59	606
34866	0.82	na		1.43	1.72	90	911
34867	0.47	na		0.94	1.04	72	830
34868	0.56	na		1.2	1.23	33	378
34869	0.61	na		1.19	1.33	39	417
34870	0.41	na		1.23	1.07	21	235
34871	0.34	na		1.06	0.82	23	244
34872	0.55	0.14		0.74	1.11	10	232
34873	0.41	na		0.69	0.87	275	3095
34874	0.53	na		0.96	1.12	245	2515
34875	0.53	na		0.89	1.08	306	3252
34876	0.68	na		1.32	1.45	45	463
34877	1.26	na	1.06	1.92	2.51	556	6167
34878	0.76	na	0.56	1.25	1.61	55	680
34879	0.39	na	0.19	0.8	0.95	23	249
34880	0.47	na	0.27	0.85	1.04	59	609
34881	0.43	na	0.23	0.82	1	41	418
34882	0.66	na	0.46	1.12	1.38	69	784
34883	0.7	na	0.50	1.18	1.47	46	507
34884	0.94	na	0.74	1.56	1.92	116	1233
34885	0.68	na	0.48	1.19	1.44	54	587
34886	0.73	na	0.53	1.31	1.55	42	441
34887	0.68	na	0.48	1.16	1.42	69	763
34888	0.96	na	0.76	1.55	1.98	65	660
34889	0.59	0.00	0.39	1.13	1.38	32	381
34890	0.68	0.00	0.48	1.18	1.46	80	926
34891	0.87	0.01	0.67	1.48	1.85	64	814
34891	0.67	na	0.47	1.27	1.36	15	176
34893	0.59	0.01	0.39	1.02	1.29	77	922
34894	0.42	0.00	0.22	0.81	0.98	66	752
34895	0.72	na	0.52	1.13	1.45	46	684
34896	0.34	0.01	0.14	0.6	0.78	80	987
34897	0.56	0.01	0.36	1.03	1.31	57	673
34898	0.66	0.00	0.46	0.99	1.33	493	3489
34899	0.78	0.01	0.58	1.21	1.58	256	2975
34900	0.81	0.01	0.61	1.29	1.66	250	2915
34901	0.66	0.01	0.46	1.03	1.37	220	2847
34902	0.64	0.00	0.44	0.98	1.33	219	1096
34903	0.94	0.01	0.74	1.41	1.9	134	1692
34904	0.53	0.00	0.33	0.81	1.07	145	1754
34905	0.37	0.01	0.17	0.58	0.78	150	1780
34906	0.34	0.01	0.14	0.56	0.71	269	3356
34907	0.17	0.01	-0.03	0.32	0.41	87	1043

34908	0.18	0.00	-0.02	0.34	0.44	285	3480
34909	0.25	0.02	0.05	0.43	0.55	96	1100
34910	0.57	0.01	0.37	0.93	1.18	70	786
34911	0.49	0.00	0.29	0.87	1.05	99	1261
34912	0.21	0.01	0.01	0.41	0.47	116	1525
34913	0.57	0.00	0.37	0.92	1.19	64	780
34914	0.85	0.00	0.65	1.32	1.73	60	726
34915	0.69	0.01	0.49	1.07	1.43	26	337
34916	0.74	0.01	0.54	1.18	1.54	29	346
34917	0.57	0.00	0.37	0.94	1.21	50	618
34918	0.62	0.01	0.42	0.96	1.29	42	485
34919	0.85	0.01	0.65	1.29	1.73	40	450
34920	0.69	0.00	0.49	1.09	1.41	80	999
34922	0.75	0.00	0.55	1.17	1.54	71	2284
34923	0.9	0.01	0.70	1.42	1.84	93	855
34924	0.93	0.00	0.73	1.51	1.94	64	1130
34925	1.1	0.00	0.90	1.71	2.23	65	846
34926	0.53	0.00	0.33	0.83	1.11	37	798
34927	0.89	0.00	0.69	1.42	1.8	35	434
34928	0.71	0.01	0.51	1.17	1.47	57	442
34929	0.67	0.00	0.47	1.1	1.39	42	716
34930	0.81	0.00	0.61	1.33	1.68	46	489
34931	0.93	0.01	0.73	1.46	1.87	64	553
34932	1.11	0.01	0.91	1.75	2.25	63	676
34933	1	0.01	0.80	1.57	2.04	66	726
34934	0.91	0.01	0.71	1.4	1.87	55	760
34935	0.95	0.00	0.75	1.54	1.93	80	675
34936	1.11	0.01	0.91	1.85	2.34	62	969
34937	1.07	0.01	0.87	1.68	2.18	65	755
34938	1.08	0.00	0.88	1.69	2.17	770	793
34939	0.99	0.00	0.79	1.52	2.01	52	1286
34940	0.71	na	0.51	1.26	1.57	43	619
34941	0.96	na	0.76	1.63	2.07	25	503
34942	0.82	0.01	0.62	1.39	1.74	25	289
34943	0.72	0.01	0.52	1.19	1.52	26	297
34944	1.04	0.01	0.84	1.65	2.12	32	311
34945	0.66	na	0.46	1.21	1.45	40	399
34946	1.05	na	0.85	1.73	2.17	49	461
34947	0.84	na	0.64	1.39	1.83	37	579
34948	1.15	0.03	0.95	1.82	2.34	53	451
34949	0.99	na	0.79	1.66	2.13	45	658
34950	0.83	na	0.63	1.3	1.72	51	550
34951	1.09	0.01	0.89	1.67	2.18	143	632
34952	0.97	0.01	0.77	1.53	1.99	31	1626
34953	0.55	0.00	0.35	0.89	1.16	67	383
34954	0.78	0.00	0.58	1.25	1.6	76	820

34955	0.61	na	0.41	1.08	1.34	35	903
34956	0.69	0.01	0.49	1.11	1.4	68	424
34957	0.85	0.00	0.65	1.34	1.73	38	814
34958	0.77	0.00	0.57	1.22	1.54	157	450
34959	0.65	0.01	0.45	1.08	1.42	72	1784
34960	0.84	0.01	0.64	1.31	1.67	767	792
34961	1.22	na	1.02	2.04	2.53	102	8533
35039	0.47	na		1.24	1.09	50	530

Table S5. Zn isotope, concentration, and content data from Duoi U'Oi and Coc Moi. Corresponding laboratory number, δ^{66}Zn and SD, δ^{67}Zn, and δ^{68}Zn values (‰ JMC-Lyon), Zn concentration (µg/g), and Zn content (µg) for each sample. The δ^{66}Zn normalized values correspond to Coc Muoi's δ^{66}Zn values normalized to the global mean of the other sites (i.e., Tam Hay Marklot, Pà Hang Mountain, and Duoi U'Oi).

S-EVA	$\delta^{15}N_{enamel}$	Blank F_{sample} (%)	N content (µMol)
34818	7.7	8.4	1.9
34819	9.7	1.6	3.5
34820	7.2	5.0	2.5
34821	8.5	10.1	2.3
34822	8.8	2.2	3.9
34822	8.8	1.8	3.3
34823	7.5	3.3	4.3
34823	7.5	2.4	7.0
34824	8.7	7.3	2.9
34825	7.0	10.4	3.1
34825	6.8	8.9	3.4
34826	8.5	5.4	1.9
34827	8.2	5.6	2.6
34828	8.7	1.1	7.8
34828	8.5	1.0	6.5
34829	8.9	1.7	6.6
34829	8.8	1.4	6.6
34830	8.3	0.9	6.5
34831	7.5	1.4	5.3
34832	7.6	1.2	5.1
34833	9.4	1.1	5.1
34833	9.0	1.1	6.5
34834	6.2	7.2	3.1
34835	6.9	3.7	3.9
34836	7.3	5.5	2.9
34837	7.9	4.5	3.3
34838	8.6	4.5	4.8
34839	7.7	7.4	2.6
34840	9.0	9.7	2.6
34840	8.4	4.8	2.5
34841	6.7	2.1	5.1
34841	8.6	1.5	4.0
34842	5.9	3.8	4.0
34842	7.8	2.6	3.1
34843	8.1	2.0	4.5
34843	9.0	1.5	3.9
34844	6.8	1.7	3.9
34845	6.6	2.4	4.3
34845	7.5	1.4	3.7
34846	6.7	2.0	4.9
34846	7.7	1.5	4.0
34847	7.1	1.9	3.1

34848	4.5	2.6	3.3
34848	4.7	2.0	2.6
34849	11.6	5.9	7.4
34850	11.3	0.8	6.0
34851	12.0	1.0	7.3
34851	13.7	0.8	6.0
34852	12.8	1.0	4.8
34853	10.8	2.6	5.8
34854	12.5	0.8	7.0
34855	11.3	2.0	5.4
34856	10.4	1.0	5.5
34857	11.7	0.7	9.6
34858	7.4	0.8	7.2
34859	7.1	1.4	7.9
34859	8.3	1.0	6.2
34860	8.9	1.3	8.9
34860	10.0	1.2	6.7
34861	6.6	2.3	8.0
34861	8.1	1.0	6.4
34862	5.8	2.2	6.9
34862	6.7	1.9	5.8
34863	5.0	10.2	1.1
34864	6.3	12.1	1.4
34865	7.2	6.0	1.6
34865	4.6	5.7	2.5
34866	3.7	8.1	1.7
34867	4.7	7.4	1.7
34868	4.5	6.7	2.2
34869	4.6	10.6	1.3
34870	7.1	2.0	2.6
34871	8.5	6.4	1.6
34872	7.1	2.9	2.3
34872	6.3	2.6	2.8
34873	5.7	1.1	6.4
34874	5.3	1.2	8.9
34874	7.0	1.0	7.0
34875	5.2	1.6	7.5
34875	6.9	0.6	12.4
34876	9.0	4.7	3.7
34876	7.2	4.2	5.0
34877	8.9	0.7	6.0
34878	8.1	1.2	4.7
34878	8.4	0.8	5.1
34879	7.1	1.1	5.0
34879	6.1	0.8	4.8
34880	6.0	1.1	5.1

34881	6.8	1.1	4.8
34882	8.1	0.7	7.2
34883	8.2	0.8	6.7
34884	8.0	1.9	3.0
34885	7.1	2.8	2.7
34885	6.6	1.5	3.1
34886	6.5	1.4	3.9
34887	7.1	1.9	3.2
34887	6.3	1.7	3.4
34888	9.6	1.1	3.5
34889	7.1	1.6	3.6
34890	8.5	1.5	4.2
34891	10.0	3.0	3.3
34891	7.0	0.7	4.8
34892	8.4	1.1	3.7
34893	8.6	1.7	3.6
34893	8.3	1.2	3.5
34894	7.6	0.9	4.1
34895	9.6	1.1	4.3
34896	9.4	1.4	4.5
34897	9.2	2.1	3.8
34897	8.7	1.0	4.1
34898	7.9	3.8	3.5
34898	8.2	2.5	4.3
34899	6.8	0.7	6.1
34900	6.7	0.7	5.9
34901	7.8	1.1	4.7
34901	7.7	1.0	5.2
34902	8.0	1.0	8.3
34903	7.5	0.9	4.3
34904	8.6	1.3	5.4
34904	8.2	0.6	5.9
34905	8.2	1.0	6.6
34905	8.2	0.6	7.1
34906	7.3	0.5	8.8
34907	11.1	1.6	6.2
34908	10.1	0.9	5.1
34909	11.3	1.1	5.2
34909	11.3	0.9	5.5
34910	13.2	1.1	5.9
34911	9.0	2.1	5.0
34912	10.6	1.4	5.4
34912	10.4	1.0	4.2
34913	5.4	1.2	3.5
34914	4.2	1.0	5.1
34915	7.0	1.4	3.7

34916	7.7	1.1	3.2
34917	7.1	4.0	3.3
34917	7.0	1.2	3.4
34918	7.7	1.7	3.4
34919	4.4	1.6	3.7
34920	5.1	3.2	3.1
34920	5.3	1.8	3.0
34922	7.0	1.1	4.2
34923	7.1	1.5	4.4
34924	6.2	1.2	5.4
34924	5.5	0.8	5.3
34925	9.4	0.7	5.4
34926	7.8	1.2	5.5
34926	7.4	0.7	5.8
34927	8.8	0.8	6.8
34928	7.2	0.4	8.4
34929	8.3	0.9	6.2
34930	9.1	0.7	6.2
34931	9.1	1.9	6.2
34932	8.0	2.0	4.7
34932	8.3	1.3	4.7
34933	7.7	0.6	6.3
34934	7.6	0.7	5.5
34935	7.8	3.3	2.6
34936	9.2	2.6	3.8
34937	7.5	1.2	6.3
34937	7.3	0.7	7.4
34938	9.1	2.4	3.3
34939	8.3	2.8	3.9
34940	7.4	4.3	3.1
34941	7.3	1.7	4.1
34941	7.7	1.1	4.0
34942	5.5	4.0	1.6
34943	7.2	0.8	5.0
34944	5.5	0.7	5.2
34945	8.8	1.1	3.3
34946	6.5	2.0	4.2
34947	5.6	1.1	4.3
34948	4.9	5.5	1.4
34949	7.7	2.1	4.2
34949	7.3	0.9	4.3
34950	5.4	0.8	4.7
34951	7.6	3.5	2.3
34952	7.9	0.7	6.3
34953	5.0	2.2	2.7
34953	4.6	1.4	2.7

34954	5.4	4.1	2.2
34955	6.9	2.3	3.0
34956	4.6	6.8	1.3
34957	7.8	2.2	3.0
34957	7.1	1.2	2.9
34958	8.0	0.8	7.9
34959	4.5	1.3	2.8
34960	7.7	3.9	5.7
34961	6.1	1.0	7.9
35039	7.7	3.8	4.0
35039	6.5	2.1	6.5

Table S6. Nitrogen isotope and related data from Duoi U'Oi and Coc Moi. Corresponding laboratory number, fraction of the blank on the total N content (%), N content (µMol), and $\delta^{15}N_{enamel}$ values (‰ AIR) for each sample (*n* = 144) and replicate (*n* = 47, for a total of *n* = 191).

ID	δ^{66}Zn	δ^{66}Zn SD	δ^{67}Zn	δ^{68}Zn	[Zn] µg/g
AZE_39	1.56	0.00	2.58	3.14	171
AZE_40	1.56	0.01	2.53	3.13	165
AZE_41	1.58	0.01	2.62	3.25	138
AZE_42	1.63	0.01	2.59	3.30	143
AZE_43	1.61	0.02	2.65	3.28	144
AZE_44	1.62	0.01	2.55	3.26	144
AZE_46	1.60		2.46	3.24	139
AZE_47	1.58		2.33	3.19	134
AZE_48	1.59		2.35	3.18	136
AZE_49	1.56		2.43	3.16	140
NIST-SRM 1400 107	0.92	0.02	1.64	1.91	137
NIST-SRM 1400 108	0.91	0.01	1.59	1.88	122
NIST-SRM 1400 112	0.92	0.00	1.54	1.92	163
NIST-SRM 1400 113	0.94	0.00	1.63	1.98	167
NIST-SRM 1400 114	0.93	0.01	1.52	1.94	170
NIST-SRM 1400 124	0.95		1.51	1.93	178
NIST-SRM 1400 126	0.99		1.49	2.04	162
NIST-SRM 1400 127	0.96		1.47	1.92	160
NIST-SRM 1400 130	0.94		1.47	1.96	171

Table S7. Zinc reference materials data. Corresponding laboratory ID, δ^{66}Zn and standard deviation (SD), δ^{67}Zn, and δ^{68}Zn values (‰ JMC-Lyon), and Zn concentration (µg/g) for reference material sample (NIST-SRM 1400 and in-house bovine liver AZE).

ID	Std. type	$\delta^{15}N$	n	SD	$\delta^{15}N$ longterm average	$\delta^{15}N$ longterm SD
CF1	Coral	5.7	17	0.1	5.6	0.1
AG-Lox	Modern Tooth Enamel	3.7	17	0.6	4.2	0.4
Mammy	Modern Tooth Enamel	6.2	15	0.4	6.8	0.4
PO2	Coral	11.9	19	0.2	11.7	0.1
USGS-40	Amino acid	-4.4	18	0.8	-4.5	0.1
USGS-65	Amino acid	20.0	18	1.1	20.7	0.1

Table S8. Nitrogen reference materials data. Corresponding laboratory ID, $\delta^{15}N$ (‰ AIR) and standard deviation (SD), and internal long-term $\delta^{15}N$ (‰ AIR) values and SD for reference material sample (in-house coral CF1 and PO2, in-house enamel AG-Lox and Mammy, and international amino acid IAEA USGS-40 and USGS-65).

**Predictor**	**Estimate (β)**	**Standard Error**	**Degrees of Freedom (df)**	***t*-value**	***p*-value**	***Lower CI***	***Upper CI***
Intercept	1.524	0.245	16	6.221	1.22e-05	1.07	1.98
Status: Extinct(1)	-0.613	0.208	16	-2.943	0.0096	-1.00	-0.23
Diet: Omnivore(2)	0.026	0.304	16	0.085	0.933	-0.53	0.59
Diet: Herbivore(2)	0.273	0.259	16	1.052	0.308	-0.20	0.75

(1) Dummy coded with "Extant" as the reference category.
(2) Dummy coded with "Carnivore" as the reference category.

Table S9. Fixed effects from the linear mixed model (LMM) testing the effect of species' status (extant vs. extinct (including both globally and regionally extinct (i.e., extirpated) taxon) and dietary category (carnivore, omnivore, herbivore) on isotope range. Taxon was included as a random intercept to account for species-level variation. The model was fit using restricted maximum likelihood (REML), and *p*-values were computed using Satterthwaite's approximation of degrees of freedom. The intercept represents the mean isotope range for extant carnivores (the reference group). Confidence intervals (95%) are based on profile likelihood estimates.

**Random Effect**	**Variance (σ²)**	**Standard Deviation (1 σ)**	**Lower CI**	**Upper CI**
Taxon	0.074	0.272	0.00	0.44
Residual	0.491	0.701	0.59	0.84

Table S10. Random effects variance components from the LMM. The random intercept for taxon captures species-specific deviations from the overall fixed effects structure. The residual variance represents within-species variation in isotope range not explained by the fixed effects. Variance, standard deviation (1 σ), and confidence intervals (95%; CI) are reported for each random component.

Supplementary Material – Text 1: δ^{13}C species-specific enrichment factors

Trophic enrichment in δ^{13}C between diet and consumer varies both across animal taxa and among different tissue types (*114*), with several comparative modern datasets existing to support this. More recently, Tejada-Lara et al. (*115*) proposed a predictive model linking δ^{13}C enrichment to body mass in mammals, offering species-specific diet-to-enamel enrichment factors.

In this study, δ^{13}C values derived from previously published sources were adjusted using the body mass-based enrichment equations of Tejada-Lara et al. (*115*) (see $\delta^{13}C_{carbon\ source}$ and δ^{13}C values in **Supplementary Material table S2** and **S4**). These equations rely on the natural logarithm of body mass (ln BM), and the resulting enrichment factor (ε*) is subsequently exponentiated to yield the value in per mil (‰). The following equations were applied:

General: $\varepsilon^*_{diet\text{-}bioapatite} = e^{2.4 + 0.034\ (\ln BM)}$

Foregut fermenter: $\varepsilon^*_{diet\text{-}bioapatite} = e^{2.34 + 0.05\ (\ln BM)}$

Hindgut fermenter: $\varepsilon^*_{diet\text{-}bioapatite} = e^{2.42 + 0.032\ (\ln BM)}$

For *Ailuropoda melanoleuca* (giant panda), we did not apply the Tejada-Lara equation, as the authors themselves caution that the resulting enrichment estimate for this species is inaccurate. Instead, we used the empirically derived value of +9.7 ‰ from Han et al. (*116*). For members of the carnivore guild, including Canidae, large and small Felidae (e.g., *Panthera pardus*, *Panthera tigris*), and Ursidae, we first added the 1.3 ‰ enrichment factor between prey and predator (*117*) and then used the general formula to get δ^{13}C values in line with vegetation values, as done for other species.

Supplementary Material – Text 2: Animal ecological flexibility during the Last Glacial Period

The ability of each taxon to adapt over time is explored for each species through changes in δ^{66}Zn, δ^{13}C, δ^{18}O, and $\delta^{15}N_{enamel}$ values across all studied sites: Tam Hay Marklot, Pà Hang Mountain, Tam Pà Ling, Nam Lot, Duoi U'Oi, and Coc Muoi. The δ^{13}C and δ^{18}O values are taken from Bacon et al. (*10*, *46*) and Bourgon et al. (*27*, *28*), and δ^{13}C values are converted to those of the food web's primary carbon sources for better comparability between species (see Bacon et al. (*10*) and Bourgon et al. (*28*) for details). The δ^{66}Zn and $\delta^{15}N_{enamel}$ values from Tam Hay Marklot are taken from Bourgon et al. (*27*) and Leichliter et al. (*19*), and the δ^{66}Zn values from Tam Pà Ling and Nam Lot from Bourgon et al. (*28*). Large-sized Bovidae (which can consist of *Bos javanicus*, *Bos gaurus*, *Bos sauveli*, and *Bubalus arnee*), sambar deer (*Rusa unicolor*), orangutans (*Pongo* sp.), giant tapirs (*Megatapirus* sp.), and stegodons (*Stegodon* sp.) are excluded here, as they are already discussed in detail in the main manuscript.

Extant species

Caprinae

Among extant species, large-sized Bovidae, Caprinae, including serows (*Capricornis* sp.) and gorals (*Naemorhedus* sp.), and porcupines (Hystricidae) exhibit the largest variability in their isotopic values (**figs. S9-16**). Despite this variability, caprine isotopic ranges (**fig. S9**) are notably broad only in the later periods, at Tam Pà Ling and Tam Hay Marklot. Specifically, the large variability and elevated δ^{13}C values at those sites suggest foraging in more diverse environments, while the δ^{66}Zn values indicate a wider variety of resources (e.g., leaves, stems, grasses, fruits, etc.). However, at Coc Muoi, we observe tightly clustered δ^{13}C values associated with greater variability in δ^{66}Zn values. Between Coc Muoi and Duoi U'Oi, δ^{13}C and δ^{66}Zn values are similar, but lower $\delta^{15}N_{enamel}$ values are observed at the latter site. A possible explanation could be a diet with a higher proportion of stems, which typically exhibit lower δ^{15}N values than leaves from the same plant (*49, 53*). Overall, all isotope systems highlight the exceptional ecological flexibility of caprines, demonstrating their capability of thriving in different habitats while consuming a variety of plant species or/and organs.

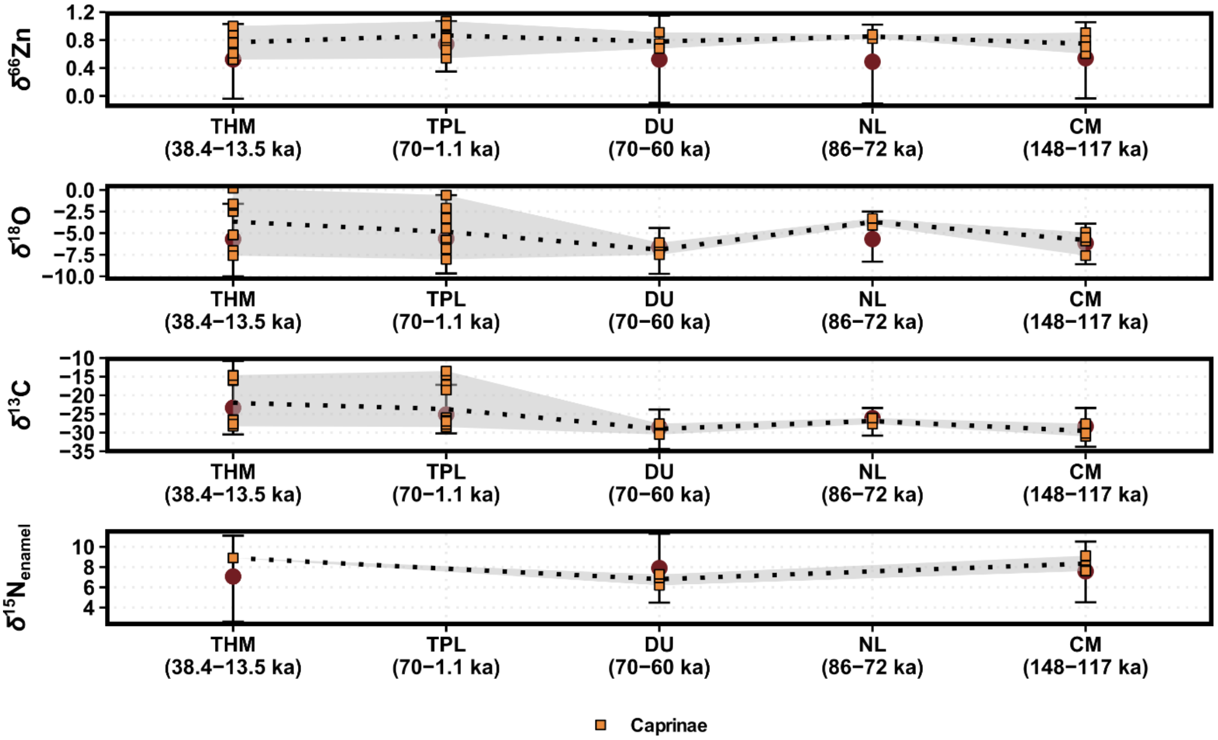

Figure S9. The $\delta^{15}N_{enamel}$ (‰ AIR), $\delta^{66}Zn$ (‰ JMC-Lyon), $\delta^{13}C$ (‰ VPDB), and $\delta^{18}O$ (‰ VPDB) values of caprine (Caprinae) between sites. The $\delta^{13}C$ values are converted to those of the food web's primary carbon sources for better comparability between species, and both $\delta^{13}C$ and $\delta^{18}O$ values are taken from Bacon et al. *(*10*,* 46*)* and Bourgon et al. *(*27*,* 28*)*. The $\delta^{66}Zn$ values from Tam Hay Marklot are taken from Bourgon et al. *(*27*)*, and those from Nam Lot and Tam Pà Ling from Bourgon et al. *(*28*)*. The $\delta^{15}N_{enamel}$ values from Tam Hay Marklot are taken from Leichliter et al. *(*19*)*. The whiskers represent the full range of values for each site, and the red dots correspond to the mean of each site. The dotted line is the moving average of caprines' values between sites, while the shaded area delimits the maximum and minimum values for caprines found between two sites. Tam Hay Marklot is denoted with the abbreviation THM, Tam Pà Ling with TPL, Duoi U'Oi with DU, Nam Lot with NL, and finally, Coc Muoi with CM.

Hystricidae

Porcupines (Hystricidae, most likely *Hystrix brachyura* and/or *Atherurus macrourus*) are found throughout Southeast Asia within various habitats, from forests to open areas. Within our dataset, they exhibit large variability in their $\delta^{66}Zn$, $\delta^{18}O$, and $\delta^{15}N_{enamel}$ values, but a narrow range of $\delta^{13}C$ values (**fig. S10**). This underscores the limitation of $\delta^{13}C$ values as a sole dietary tracer, reinforcing that they are better suited for identifying or reconstructing the (paleo)environments in which animals foraged rather than their diet. At Duoi U'Oi and Tam Hay Marklot, lower $\delta^{66}Zn$ values compared to other sites may suggest foraging on resources not usually preferred or perhaps no longer present or abundant. Some of the $\delta^{66}Zn$ values are quite low, occasionally overlapping with those of carnivores, though this pattern is not reflected in $\delta^{15}N_{enamel}$ values, which consistently indicate an herbivorous diet. The low $\delta^{66}Zn$ values could indicate a low proportion of animal-matter (including insects) in their diet, but not enough to cause a trophic level shift in $\delta^{15}N_{enamel}$ values. The large variability in porcupine $\delta^{18}O$ values likely results from different seasons recorded in their ever-growing incisor (the dental locus mostly found and sampled) and different resources ingested, driven either by seasonal availability or the porcupine's opportunistic feeding behavior.

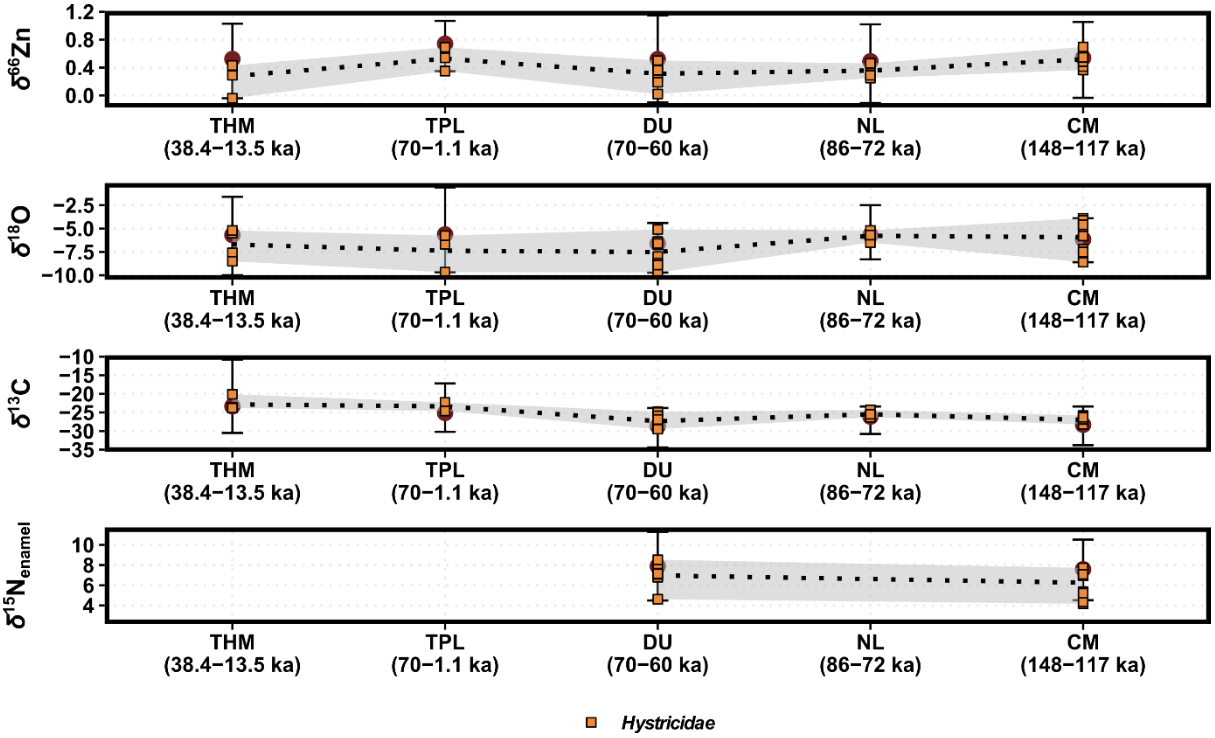

Figure S10. The $\delta^{15}N_{enamel}$ (‰ AIR), $\delta^{66}Zn$ (‰ JMC-Lyon), $\delta^{13}C$ (‰ VPDB), and $\delta^{18}O$ (‰ VPDB) values of porcupines (Hystricidae) between sites. The $\delta^{13}C$ values are converted to those of the food web's primary carbon sources for better comparability between species, and both $\delta^{13}C$ and $\delta^{18}O$ values are taken from Bacon et al. (*10*, *46*) and Bourgon et al. (*27*, *28*). The $\delta^{66}Zn$ values from Tam Hay Marklot are taken from Bourgon et al. (*27*), and those from Nam Lot and Tam Pà Ling from Bourgon et al. (*28*). The whiskers represent the full range of values for each site, and the red dots correspond to the mean of each site. The dotted line is the moving average of porcupines' values between sites, while the shaded area delimits the maximum and minimum values for porcupines found between two sites. Tam Hay Marklot is denoted with the abbreviation THM, Tam Pà Ling with TPL, Duoi U'Oi with DU, Nam Lot with NL, and finally, Coc Muoi with CM.

Sus sp.

Originally native to Eurasia, wild boars (*Sus* sp.) are now found on all continents except Antarctica, with diets varying wildly depending on habitats. Although often portrayed as a prime example of an omnivorous species, their diet is predominantly composed of various plant materials (*51*, *118*). Their $\delta^{13}C$ values remain consistently low across most sites, except at Tam Hay Marklot where higher values (**fig. S11**) suggest foraging in more diverse environments. The lowest $\delta^{15}N_{enamel}$ values are observed at Tam Hay Marklot, with similar and variable values at Coc Muoi and Duoi U'Oi (**fig. S11**). The $\delta^{66}Zn$ values vary across sites, but both $\delta^{66}Zn$ and $\delta^{15}N_{enamel}$ values suggest an herbivorous diet, in line with available ecological data (*51*, *118*). The narrower range in $\delta^{66}Zn$ values at Nam Lot could indicate a more restricted diet, possibly due to fewer available resources. The $\delta^{18}O$ values progressively vary more and more within sites as time progresses, which could suggest the consumption of resources with differing levels of chemically-bound oxygen (e.g., leaves vs. grasses). Although it does not align perfectly, $\delta^{18}O$ values follow more closely trends in $\delta^{66}Zn$ values than with $\delta^{13}C$ and $\delta^{15}N_{enamel}$ values (**fig. S11**).

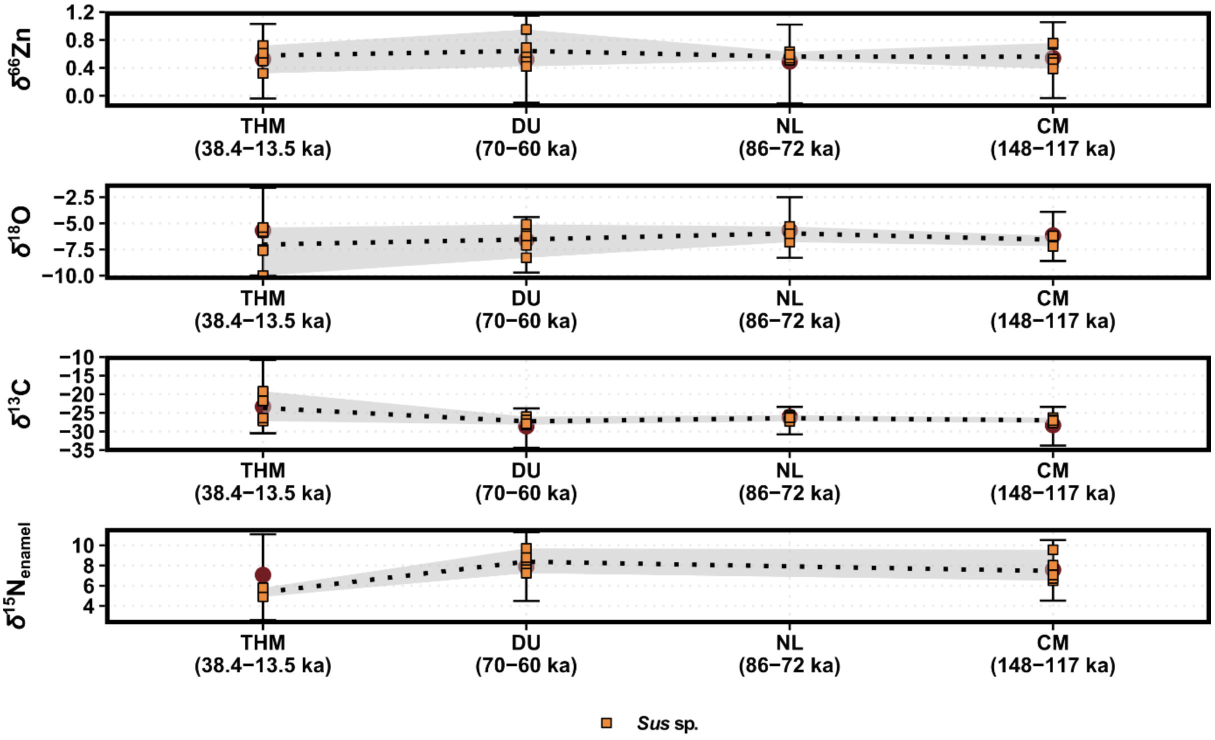

Figure S11. The $\delta^{15}N_{enamel}$ (‰ AIR), $\delta^{66}Zn$ (‰ JMC-Lyon), $\delta^{13}C$ (‰ VPDB), and $\delta^{18}O$ (‰ VPDB) values of wild boars (*Sus* sp.) between sites. The $\delta^{13}C$ values are converted to those of the food web's primary carbon sources for better comparability between species, and both $\delta^{13}C$ and $\delta^{18}O$ values are taken from Bacon et al. (*10*, *46*) and Bourgon et al. (*27*, *28*). The $\delta^{66}Zn$ values from Tam Hay Marklot are taken from Bourgon et al. (*27*), and those from Nam Lot from Bourgon et al. (*28*). The $\delta^{15}N_{enamel}$ values from Tam Hay Marklot are taken from Leichliter et al. (*19*). The whiskers represent the full range of values for each site, and the red dots correspond to the mean of each site. The dotted line is the moving average of boars' values between sites, while the shaded area delimits the maximum and minimum values for boars found between two sites. Tam Hay Marklot is denoted with the abbreviation THM, Duoi U'Oi with DU, Nam Lot with NL, and finally, Coc Muoi with CM.

Muntiacus sp.

Muntjaks' (*Muntiacus* sp., potentially including species such as *M. rooseveltorum*, *M. truongsonensis*, *M. vaginalis*, and *M. vuquangensis*) inhabit a range of habitats and elevation levels. However, except for the northern red muntjacs (*M. vaginalis*), all other species are strictly limited to evergreen and semi-evergreen forests (*119*–*122*). Although dietary information is limited, most species are known to consume fallen fruits. The Northern red muntjaks, for which more information is available, also consume various plant parts, particularly young organs such as buds, small seeds, twigs, seed pods, tender leaves, and young grass (*123*–*126*). In our dataset, muntjaks' $\delta^{13}C$ values are similar, low, and display little variability across all sites, suggesting a preference for forest habitats (**fig. S12**). The $\delta^{66}Zn$ values show greater variability at densely forested sites (i.e., lower $\delta^{13}C$ values) and at Tam Hay Marklot (**fig. S12**). This may indicate that the environment at Nam Lot imposed a more restricted dietary niche for muntjaks, yet still supported them while they eventually had to diversify their foraging environments at Tam Hay Marklot. The $\delta^{15}N_{enamel}$ values are slightly higher at Duoi U'Oi compared to Coc Muoi (**fig. S12**), mirroring trends in $\delta^{66}Zn$ values, possibly indicating a diet including more grasses or forbs and less browse (*52, 53*). The $\delta^{18}O$ values generally follow the trend between sites (i.e., lower or higher values at sites with lower or higher overall mean), suggesting that their $\delta^{18}O$ values are

primarily influenced by drinking water. However, similar to the variability in δ^{66}Zn values, δ^{18}O values vary within each site, potentially reflecting individual small differences in diets, in this case, with resources containing varying degrees of chemically-bound oxygen (e.g., leaves vs. grasses).

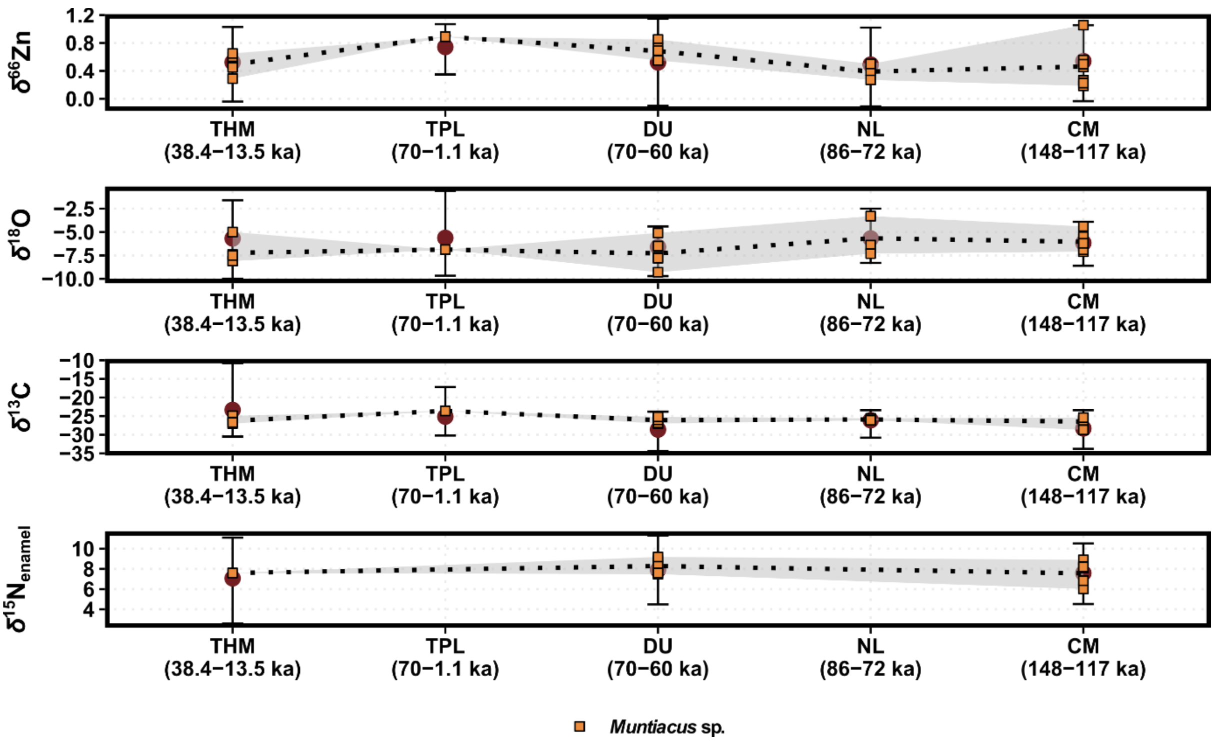

Figure S12. The $\delta^{15}N_{enamel}$ (‰ AIR), δ^{66}Zn (‰ JMC-Lyon), δ^{13}C (‰ VPDB), and δ^{18}O (‰ VPDB) values of muntjaks (*Muntiacus* sp.) between sites. The δ^{13}C values are converted to those of the food web's primary carbon sources for better comparability between species, and both δ^{13}C and δ^{18}O values are taken from Bacon et al. (*10*, *46*) and Bourgon et al. (*27*, *28*). The δ^{66}Zn values from Tam Hay Marklot are taken from Bourgon et al. (*27*), and those from Nam Lot and Tam Pà Ling from Bourgon et al. (*28*). The $\delta^{15}N_{enamel}$ values from Tam Hay Marklot are taken from Leichliter et al. (*19*). The whiskers represent the full range of values for each site, and the red dots correspond to the mean of each site. The dotted line is the moving average of muntjaks' values between sites, while the shaded area delimits the maximum and minimum values for muntjaks found between two sites. Tam Hay Marklot is denoted with the abbreviation THM, Tam Pà Ling with TPL, Duoi U'Oi with DU, Nam Lot with NL, and finally, Coc Muoi with CM.

Macaca sp.

Because the species identification is uncertain for the macaques (*Macaca* sp., which could include species such as *M. mulatta*, *M. assamensis*, *M. arctoides*, *M. leonina*, and *M. fascicularis*), comparisons with modern species are complicated because of the wide range of ecosystems they inhabit and the diversity of food resources they forage. The macaques' δ^{66}Zn values vary greatly within and between sites, with low values overlapping those of carnivores (**fig. S13**). At Duoi U'Oi, those low values are also associated with high $\delta^{15}N_{enamel}$ values (**fig. S13**), some of them falling in the range of those of carnivores. While not all specimens follow this pattern, it seemingly confirms that low δ^{66}Zn values within omnivore species are associated with a higher proportion of animal-matter (which could include insects) in their diet. The δ^{13}C values are relatively similar across sites, whereas δ^{18}O values show a greater variability at Duoi U'Oi, Nam Lot, and Coc Muoi (**fig. S13**). Large δ^{66}Zn variability at Duoi U'Oi

and Coc Muoi suggests a diverse diet, which could also account for the variable δ^{18}O values at these sites (**Figure S13**), and agrees with general perspectives that this taxon thrived through time (*77*).

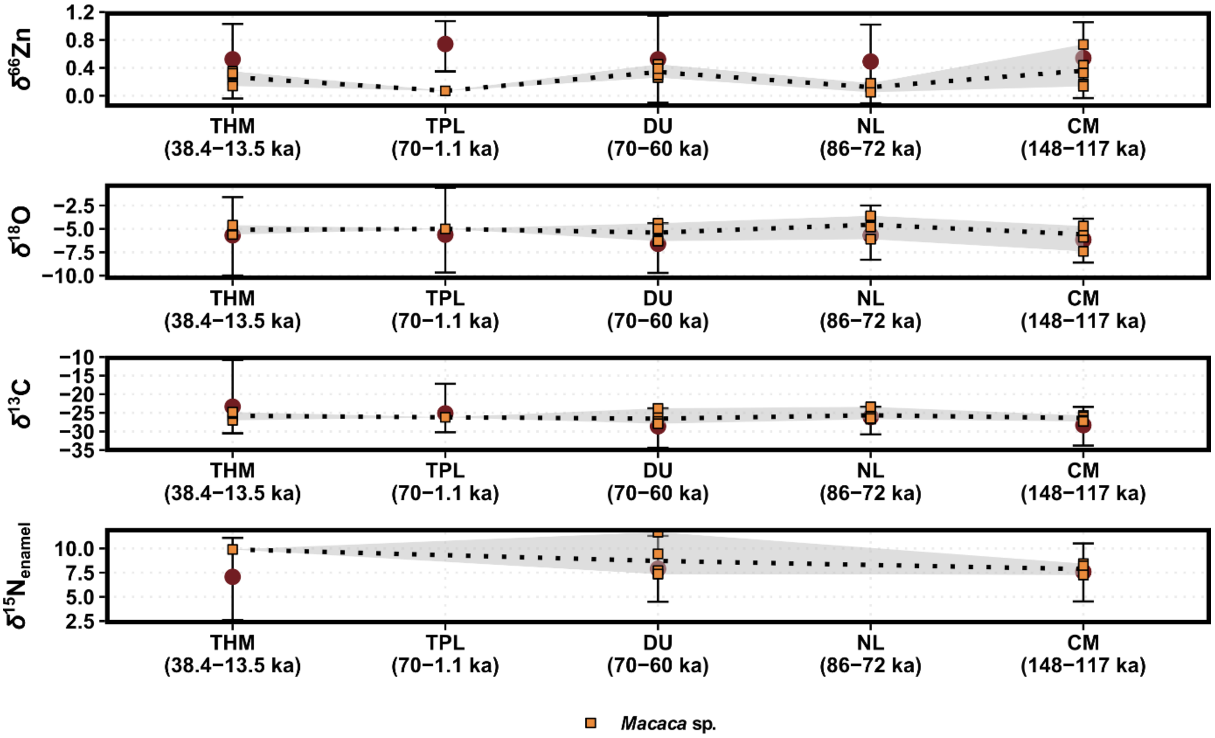

Figure S13. The $\delta^{15}N_{enamel}$ (‰ AIR), δ^{66}Zn (‰ JMC-Lyon), δ^{13}C (‰ VPDB), and δ^{18}O (‰ VPDB) values of macaques (*Macaca* sp.) between sites. The δ^{13}C values are converted to those of the food web's primary carbon sources for better comparability between species, and both δ^{13}C and δ^{18}O values are taken from Bacon et al. (*10*, *46*) and Bourgon et al. (*27*, *28*). The δ^{66}Zn values from Tam Hay Marklot are taken from Bourgon et al. (*27*), and those from Nam Lot and Tam Pà Ling from Bourgon et al. (*28*). The $\delta^{15}N_{enamel}$ values from Tam Hay Marklot are taken from Leichliter et al. (*19*). The whiskers represent the full range of values for each site, and the red dots correspond to the mean of each site. The dotted line is the moving average of macaques' values between sites, while the shaded area delimits the maximum and minimum values for macaques found between two sites. Tam Hay Marklot is denoted with the abbreviation THM, Tam Pà Ling with TPL, Duoi U'Oi with DU, Nam Lot with NL, and finally, Coc Muoi with CM.

Ursus sp.

Bears (*Ursus* sp., likely the Asiatic black bear, *U. thibetanus*) are found throughout Asia, occupying various forested habitats and moving seasonally to different habitats and elevations based on food abundance. In the tropical and subtropical regions, their diet is typically dominated by fruits (*127*), though this can vary with food availability (*128*). In some populations, a substantial proportion of animal matter may be included in their diet, primarily through scavenging on ungulate carcasses or eating ants or bees (*129–132*). Their δ^{66}Zn and $\delta^{15}N_{enamel}$ values show a similar pattern to those of the macaques (**fig. S14**), further supporting that low δ^{66}Zn values within omnivores are associated with a higher proportion of animal-matter in the diet. Their δ^{13}C values are low and similar across sites (**fig. S14**), but their δ^{18}O values vary within Duoi U'Oi, Nam Lot, and Coc Muoi (**fig. S14**). As seen with the macaques and the bears, their δ^{13}C values illustrate well that δ^{13}C values alone are better suited to establishing (paleo)environments where animals forage rather than their specific diet. Except at Tam

Hay Marklot, bears mostly show low δ^{18}O values, which could align well with feeding behaviors associated with foraging on the forest floor, and appear disconnected from the otherwise small range in δ^{13}C values.

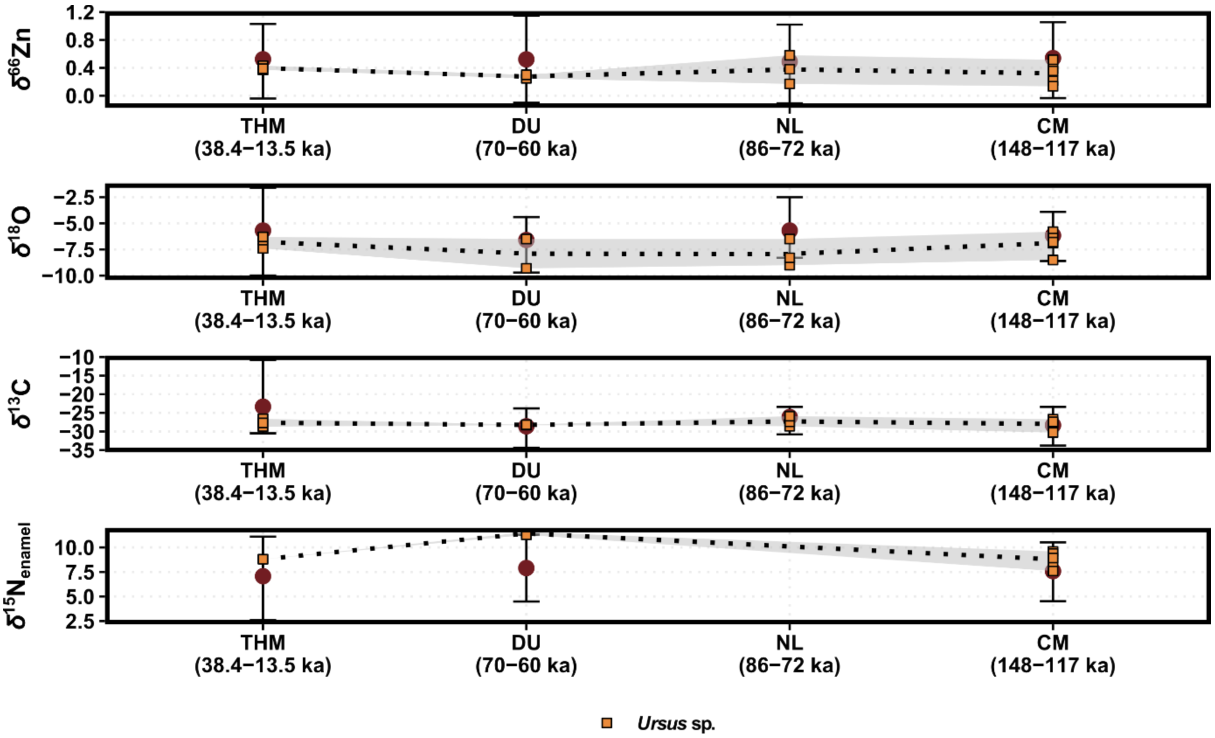

Figure S14. The $\delta^{15}N_{enamel}$ (‰ AIR), δ^{66}Zn (‰ JMC-Lyon), δ^{13}C (‰ VPDB), and δ^{18}O (‰ VPDB) values of bears (*Ursus* sp.) between sites. The δ^{13}C values are converted to those of the food web's primary carbon sources for better comparability between species, and both δ^{13}C and δ^{18}O values are taken from Bacon et al. (*10*, *46*) and Bourgon et al. (*27*, *28*). The δ^{66}Zn values from Tam Hay Marklot are taken from Bourgon et al. (*27*), and those from Nam Lot from Bourgon et al. (*28*). The $\delta^{15}N_{enamel}$ values from Tam Hay Marklot are taken from Leichliter et al. (*19*). The whiskers represent the full range of values for each site, and the red dots correspond to the mean of each site. The dotted line is the moving average of bears' values between sites, while the shaded area delimits the maximum and minimum values for bears found between two sites. Tam Hay Marklot is denoted with the abbreviation THM, Duoi U'Oi with DU, Nam Lot with NL, and finally, Coc Muoi with CM.

Canidae

Among extant species, Canidae (dhole, *Cuon alpinus)*, small-sized Felidae (possibly clouded leopards, *Neofelis nebulosa*, or leopard cats, *Prionailurus bengalensis)*, and elephants (*Elephas* sp.) show the least isotopic variability. However, the small sample size for the latter two taxa (n = 3 and n = 5, respectively) arguably limits comprehensive ecological assessments. Their current local conservation status in Laos and Vietnam also differs from that of other extant species. The Asian elephant population is decreasing, and the remaining population in the regions is small, fragmented, and highly threatened (*133*). The clouded leopard has become extremely rare and is likely extirpated from Laos (*134*), and with no recent records from Vietnam (*134*). Similarly, the dhole population is decreasing globally and is now confined to only two protected areas in Laos (*135*), while it is believed to be extirpated from Vietnam (*135*). Although hunting and snaring are the main threats to the small-sized Felidae (*134*), habitat loss and transformation are among the primary factors compromising elephants' and dhole's

ability to sustain their populations (*133*, *135*). Due to the limited number of small-sized felid samples, their ecological flexibility cannot be thoroughly assessed, but there are sufficient dhole and elephant samples to allow for tentative ecological evaluations.

Dholes present an interesting case among canid species, as they are one of the only three with specialized dental adaptations for an exclusively carnivorous diet, termed hypercarnivory (*136*). Variability in trophic level proxies, δ^{66}Zn and $\delta^{15}N_{enamel}$, should thus likely reflect different prey and animal tissue selection alone rather than the inclusion of plant resources. Moreover, sufficient ungulate prey biomass is believed to be the dhole's primary habitat requirement precisely because of the demands imposed by hypercarnivory. Although the dholes are known to prey on a diverse array of species with vastly different body masses (*137–140*), their preferred prey typically consists of ungulates weighing between 40 and 60 kg (*141*). When their favored prey size is not (seasonally) available or abundant enough within a given environment, dholes will selectively prey upon smaller and larger ungulates (*142*). Specifically, they appear to persist in smaller packs in tropical evergreen forests because of the low prey biomass and small size of ungulate prey compared to packs in open habitats. Comparatively, India's tropical dry and moist deciduous forests could illustrate their optimal habitats, as these areas support the largest dhole populations today (*143*, *144*). The δ^{13}C values of dhole samples remain similar across sites, while all other proxies show variability that likely indicates changes in prey selection (**fig. S15**). Despite differences in sample sizes across sites, their isotope values clearly show a larger variability at Tam Hay Marklot compared to all other sites. Based on their modern ecological preferences, lower variability would be expected for periods associated with a fragmenting of lowland rainforest into more open canopy seasonally dry forest and tropical montane forests, such as those at Tam Hay Marklot and Nam Lot. A larger sample size and additional sites, perhaps including some from India, could provide further insights into their ecological preferences and could clarify if their modern optimal habitats have always been consistent over time. Their δ^{18}O values follow the general trend between sites, suggesting that most of their δ^{18}O values primarily reflect drinking water, which is itself solely dependent on the different climatic conditions present at each site.

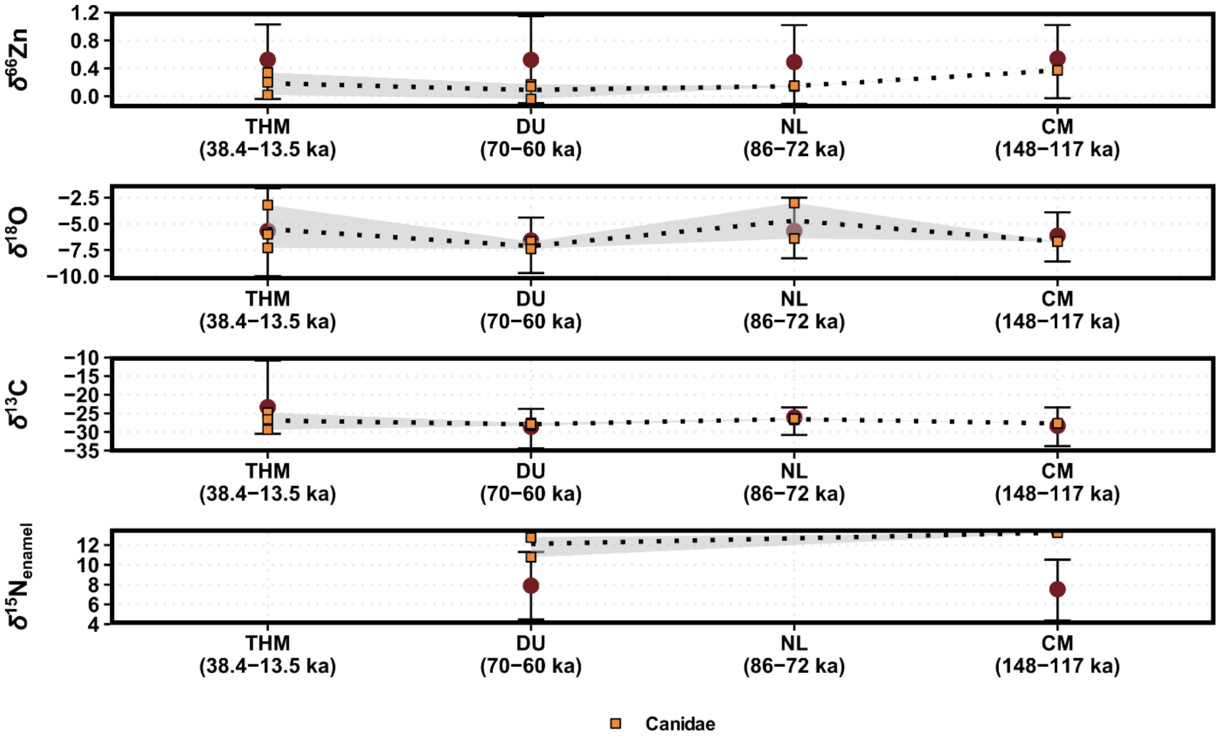

Figure S15. The $\delta^{15}N_{enamel}$ (‰ AIR), $\delta^{66}Zn$ (‰ JMC-Lyon), $\delta^{13}C$ (‰ VPDB), and $\delta^{18}O$ (‰ VPDB) values of canids (Canidae) between sites. The $\delta^{13}C$ values are converted to those of the food web's primary carbon sources for better comparability between species, and both $\delta^{13}C$ and $\delta^{18}O$ values are taken from Bacon et al. (*10*, *46*) and Bourgon et al. (*27*, *28*). The $\delta^{66}Zn$ values from Tam Hay Marklot are taken from Bourgon et al. (*27*), and those from Nam Lot from Bourgon et al. (*28*). The whiskers represent the full range of values for each site, and the red dots correspond to the mean of each site. The dotted line is the moving average of canids' values between sites, while the shaded area delimits the maximum and minimum values for canids found between two sites. Tam Hay Marklot is denoted with the abbreviation THM, Duoi U'Oi with DU, Nam Lot with NL, and finally, Coc Muoi with CM.

Elephas **sp.**

The modern Asian elephant is an herbivorous generalist, feeding on various plants depending on habitats and seasons. They inhabit a range of environments throughout the Asian continent, including grassland, tropical evergreen forests, semi-evergreen forests, moist deciduous forests, dry deciduous forests, and dry thorn forests (*145*–*148*). However, human modification of these landscapes complicates the assessment of their optimal habitat. Elephants show some degree of dietary flexibility, with different populations exhibiting varied diets between dry and wet seasons (*145*, *146*), as well as different overall annual diets (*145*–*148*). Throughout the studied period, most isotope values show minimal variation. The $\delta^{13}C$ values are low and consistent with foraging in closed-canopy forest habitats (**fig. S16**). The $\delta^{15}N_{enamel}$ values generally fall within the bulk of herbivores' values, as do the $\delta^{66}Zn$ values, except for one individual at Coc Muoi (**fig. S16**). Although the small sample size (n = 5) limits accurate assessment, all isotope proxies suggest a relatively narrow dietary niche for the Asian elephant.

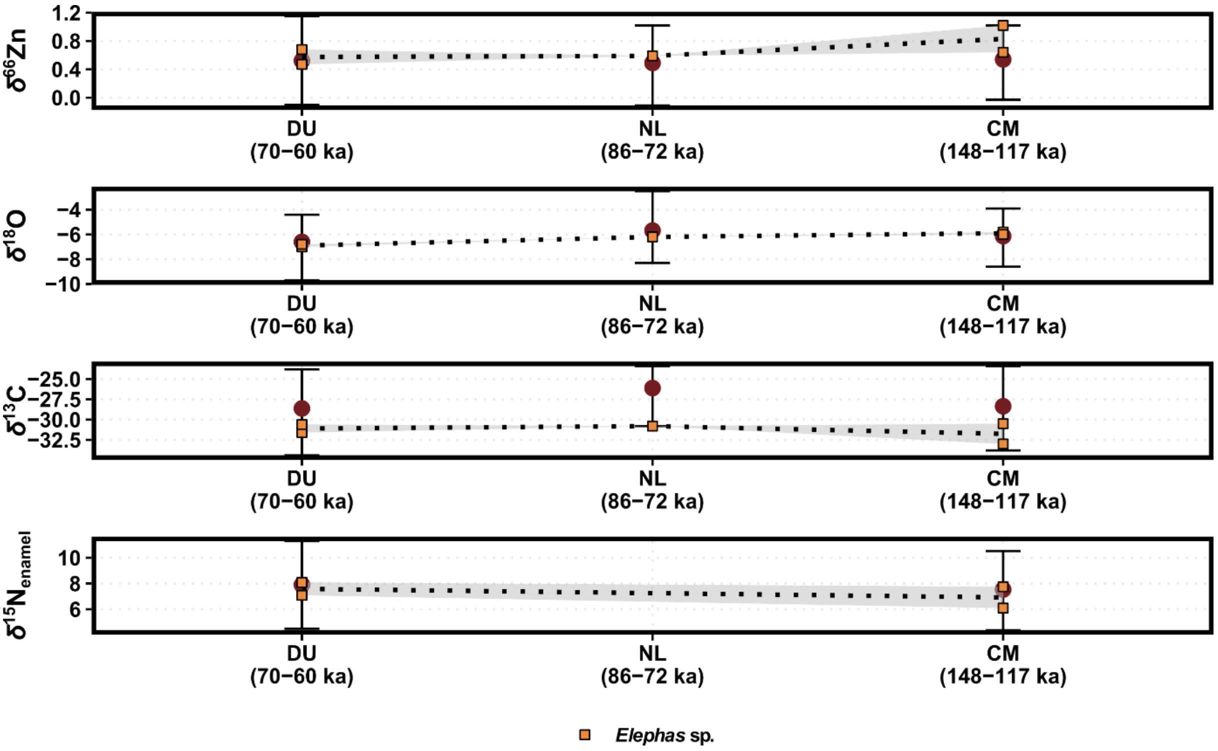

Figure S16. The $\delta^{15}N_{enamel}$ (‰ AIR), $\delta^{66}Zn$ (‰ JMC-Lyon), $\delta^{13}C$ (‰ VPDB), and $\delta^{18}O$ (‰ VPDB) values of Asian elephants (*Elephas* sp.) between sites. The $\delta^{13}C$ values are converted to those of the food web's primary carbon sources for better comparability between species, and both $\delta^{13}C$ and $\delta^{18}O$ values are taken from Bacon et al. (*10*, *46*) and Bourgon et al. (*28*). The $\delta^{66}Zn$ values those from Nam Lot from Bourgon et al. (*28*). The whiskers represent the full range of values for each site, and the red dots correspond to the mean of each site. The dotted line is the moving average of elephants' values between sites, while the shaded area delimits the maximum and minimum values for elephants found between two sites. Duoi U'Oi is denoted with the abbreviation DU, Nam Lot with NL, and finally, Coc Muoi with CM.

Extirpated species

Dicerorhinus sumatrensis

The Sumatran rhinoceros (*Dicerorhinus sumatrensis*) and the giant panda (*Ailuropoda melanoleuca*) are the two locally-extirpated species exhibiting the least variability in their isotopic values. Strictly limited to Indonesia today, the Sumatran rhinoceros inhabits tropical rainforests and montane moss forests, and they can usually be found in areas near water sources and salt licks (*149*). Their diet has been described as primarily folivorous (*150*). However, their restricted geographical range likely restricts their diet to resources available in the habitats they occupy. Within our dataset, they exhibit intermediate to high $\delta^{66}Zn$ values compared to other herbivores, suggesting a diet comprising low-growing vegetation (**fig. S17**). The low $\delta^{13}C$ values indicate a relatively strict preference for closed-canopy forest habitat (**fig. S17**). The analyzed rhinoceros teeth fragments from Nam Lot remain taxonomically unidentified, introducing uncertainty for that site and period, as those specimens could also belong to the greater one-horned rhinoceros (*Rhinoceros unicornis*) or Javan rhinoceros (*Rhinoceros sondaicus*). A large $\delta^{15}N_{enamel}$ variation is observed between Coc Muoi and Duoi U'Oi Sumatran rhinoceros samples (**fig. S17**), with values at the latter being approximately 4 ‰ lower, which may suggest a diet including more plant stems or bark. While they display intermediate $\delta^{18}O$ values

that closely align with each site's mean, the value of the rhinoceros individual at Tam Pà Ling, the latest site, is among the lowest for that site and very different from all other sites.

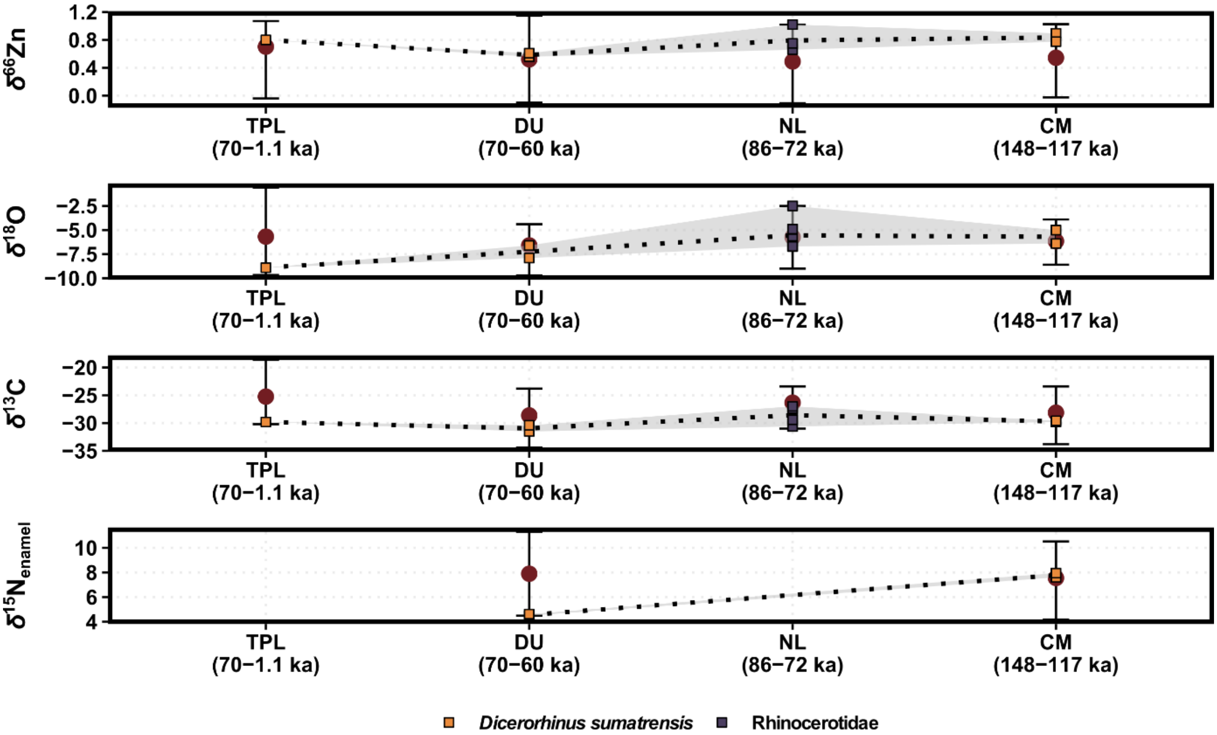

Figure S17. The $\delta^{15}N_{enamel}$ (‰ AIR), δ^{66}Zn (‰ JMC-Lyon), δ^{13}C (‰ VPDB), and δ^{18}O (‰ VPDB) values of Sumatran rhinoceros *(Dicerorhinus sumatrensis)* between sites. The δ^{13}C values are converted to those of the food web's primary carbon sources for better comparability between species, and both δ^{13}C and δ^{18}O values are taken from Bacon et al. (*10*, *46*) and Bourgon et al. (*28*). The δ^{66}Zn values from Nam Lot and Tam Pà Ling from Bourgon et al. (*28*). The whiskers represent the full range of values for each site, and the red dots correspond to the mean of each site. The dotted line is the moving average of Sumatran rhinoceros' values between sites, while the shaded area delimits the maximum and minimum values for Sumatran rhinoceros found between two sites. Rhinoceros specimens from Nam Lot (Rhinocerotidae) are not identified at the species level and thus present a certain degree of uncertainty, as those specimens could equally be greater one-horned rhinoceros (*Rhinoceros unicornis*), Javan rhinoceros (*Rhinoceros sondaicus*) or Sumatran rhinoceros (*Dicerorhinus sumatrensis*). Tam Pà Ling is denoted with the abbreviation TPL, Duoi U'Oi with DU, Nam Lot with NL, and finally, Coc Muoi with CM.

Rhinoceros sondaicus

Although today's Javan rhinoceros is found in the vicinity of water in lowland tropical rainforest areas (*151*), little is known about its preferred "natural" habitat. This species, considered a primarily herbaceous monocotyledons browser (*150*), shows the largest variability in its isotopic values among extirpated species. The δ^{13}C values stay low throughout the studied period and are associated with closed-canopy forest habitat, consistent with its modern habitat preference (**fig. S18**). They exhibit large inter- and intra-site variability in δ^{66}Zn values (**fig. S18**), which could accordingly suggest a diet including plant leaves and stems, except at Tam Pà Ling, where they show high values, typically associated with low-growing plants. Taken together, this would suggest that this species forages on different plants and plant parts, which corresponds well to its known diet that can include grasses, fruit, leaves, shrubs, and tree branches (*152*), but within one given habitat. While the δ^{18}O values closely follow the general trend between sites (i.e., lower or higher values at sites with lower or higher

overall mean), a more varied diet with a lesser or greater proportion of water obtained from the diet could explain the higher intra-site variability displayed at Coc Muoi.

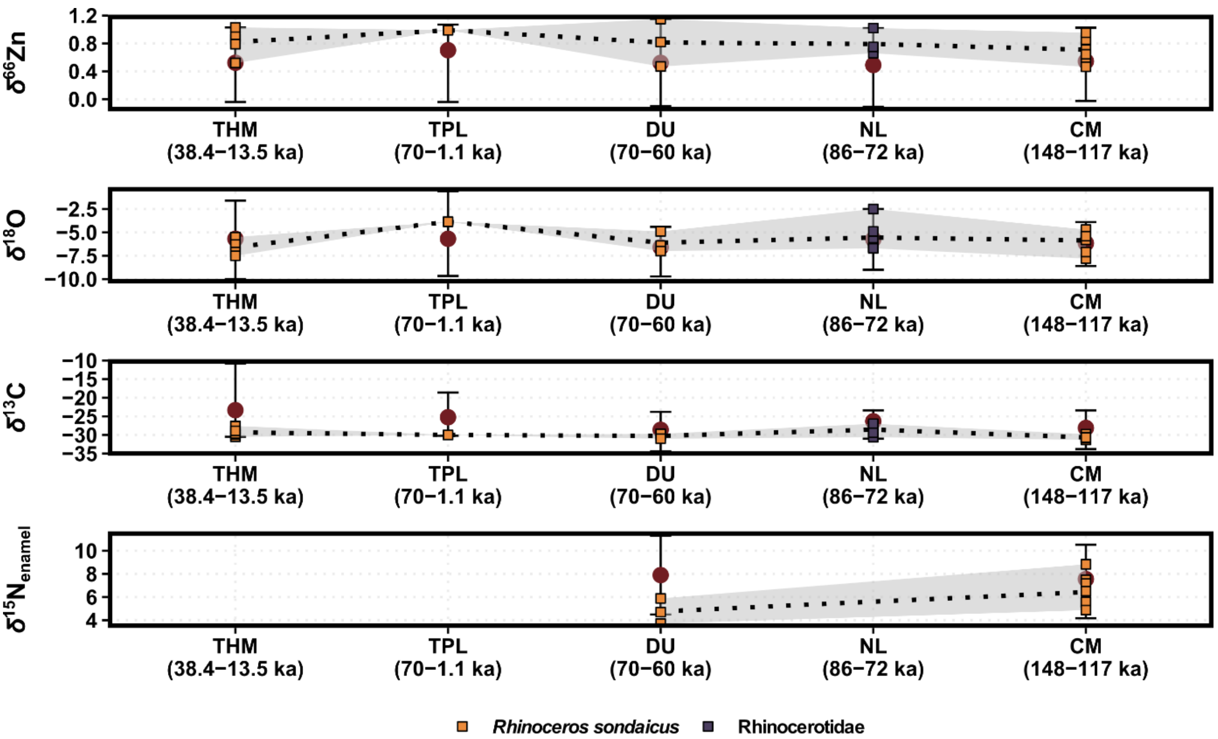

Figure S18. The $\delta^{15}N_{enamel}$ (‰ AIR), δ^{66}Zn (‰ JMC-Lyon), δ^{13}C (‰ VPDB), and δ^{18}O (‰ VPDB) values of Javan rhinoceros (*Rhinoceros sondaicus*) between sites. The δ^{13}C values are converted to those of the food web's primary carbon sources for better comparability between species, and both δ^{13}C and δ^{18}O values are taken from Bacon et al. (*10*, *46*) and Bourgon et al. (*27*, *28*). The δ^{66}Zn values from Tam Hay Marklot are taken from Bourgon et al. (*27*), and those from Nam Lot and Tam Pà Ling from Bourgon et al. (*28*). The whiskers represent the full range of values for each site, and the red dots correspond to the mean of each site. The dotted line is the moving average of Javan rhinoceros' values between sites, while the shaded area delimits the maximum and minimum values for Javan rhinoceros found between two sites. Rhinoceros specimens from Nam Lot (Rhinocerotidae) are not identified at the species level and thus present a certain degree of uncertainty, as those specimens could equally be greater one-horned rhinoceros (*Rhinoceros unicornis*), Javan rhinoceros (*Rhinoceros sondaicus*) or Sumatran rhinoceros (*Dicerorhinus sumatrensis*). Tam Hay Marklot is denoted with the abbreviation THM, Tam Pà Ling with TPL, Duoi U'Oi with DU, Nam Lot with NL, and finally, Coc Muoi with CM.

Rhinoceros unicornis

The last of the three Asian rhinoceros species analyzed, the greater one-horned rhinoceros, shows little variability in its isotopic values. It is known to inhabit alluvial plain grassland (*150*), and its δ^{66}Zn values are intermediate within herbivores range (**fig. S19**), suggesting a diet that includes plant leaves and stems, except at Tam Pà Ling, with a high value, typically associated with low-growing plants. However, their consistently low δ^{13}C values indicate a strong preference for closed-canopy forest habitats (**fig. S19**), which contrasts with their known ecological preferences for grasslands. This could suggest that this species was more of a generalist in the past or that it now thrives more in grasslands, the last habitat where they are still found today. Although more specimens would be needed to clarify the reasons for their extirpation, our data could suggest that, within closed-canopy forest habitats, the greater one-horned rhinoceroses relied on a specific food resource that became scarce over time.

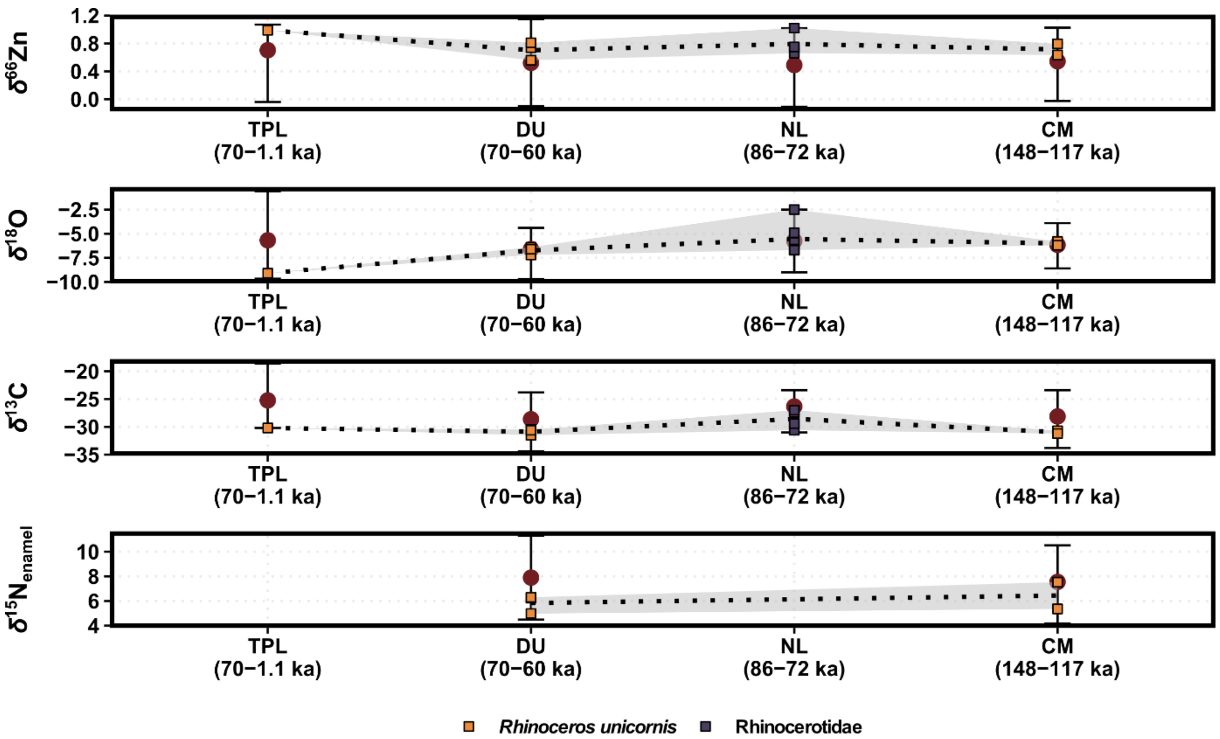

Figure S19. The $\delta^{15}N_{enamel}$ (‰ AIR), $\delta^{66}Zn$ (‰ JMC-Lyon), $\delta^{13}C$ (‰ VPDB), and $\delta^{18}O$ (‰ VPDB) values of greater one-horned rhinoceros (*Rhinoceros unicornis*) between sites. The $\delta^{13}C$ values are converted to those of the food web's primary carbon sources for better comparability between species, and both $\delta^{13}C$ and $\delta^{18}O$ values are taken from Bacon et al. (*10*, *46*) and Bourgon et al. (*28*). The $\delta^{66}Zn$ values from Nam Lot and Tam Pà Ling from Bourgon et al. (*28*). The whiskers represent the full range of values for each site, and the red dots correspond to the mean of each site. The dotted line is the moving average of greater one-horned rhinoceros' values between sites, while the shaded area delimits the maximum and minimum values for greater one-horned rhinoceros found between two sites. Rhinoceros specimens from Nam Lot (Rhinocerotidae) are not identified at the species level and thus present a certain degree of uncertainty, as those specimens could equally be greater one-horned rhinoceros (*Rhinoceros unicornis*), Javan rhinoceros (*Rhinoceros sondaicus*) or Sumatran rhinoceros (*Dicerorhinus sumatrensis*). Tam Pà Ling is denoted with the abbreviation TPL, Duoi U'Oi with DU, Nam Lot with NL, and finally, Coc Muoi with CM.

Ailuropoda melanoleuca

The giant panda (*Ailuropoda melanoleuca*) is the species showing the lowest variability in its isotopic values, in line with today's obligate bamboo specialist behaviors, with a clear reliance on this resource. Although their geographic range in the fossil record was much wider, they are nowadays solely found in temperate montane forests at altitudes of 1,500–3,000 m (*153*), especially associated with old-growth forests (*154*). Accordingly, the intermediate-to-low $\delta^{66}Zn$ and intermediate-to-low $\delta^{13}C$ values within the C_3 range are similar throughout all sites, indicating a consistent diet and ecological niche over time (**fig. S20**). The $\delta^{13}C$ values are also consistent with bamboo values (*155*).

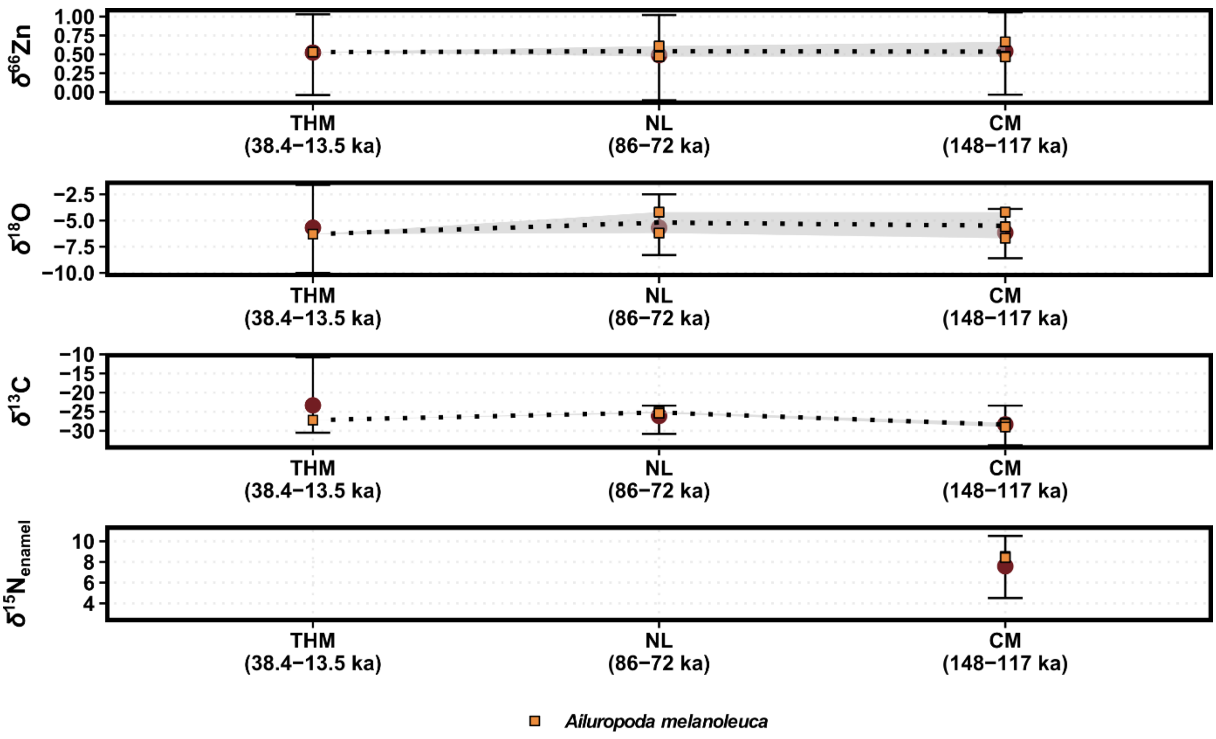

Figure S20. The $\delta^{15}N_{enamel}$ (‰ AIR), $\delta^{66}Zn$ (‰ JMC-Lyon), $\delta^{13}C$ (‰ VPDB), and $\delta^{18}O$ (‰ VPDB) values of giant pandas (*Ailuropoda melanoleuca*) between sites. The $\delta^{13}C$ values are converted to those of the food web's primary carbon sources for better comparability between species, and both $\delta^{13}C$ and $\delta^{18}O$ values are taken from Bacon et al. (*10*, *46*) and Bourgon et al. (*27*, *28*). The $\delta^{66}Zn$ values from Tam Hay Marklot are taken from Bourgon et al. (*27*), and those from Nam Lot from Bourgon et al. (*28*). The whiskers represent the full range of values for each site, and the red dots correspond to the mean of each site. The dotted line is the moving average of giant pandas' values between sites, while the shaded area delimits the maximum and minimum values for giant pandas found between two sites. Tam Hay Marklot is denoted with the abbreviation THM, Nam Lot with NL, and finally, Coc Muoi with CM.

Panthera pardus

The leopard (*Panthera pardus*) has the widest range of habitats among any of the large Old-World cats (*156*), but is predominantly found in rainforests in Southeast Asia (*156*). Their diet is usually related to prey availability and the presence of other competitors. Although they prefer medium-sized ungulate prey (10–40 kg) (*157*), their diet has been known to be highly varied (*157*). Interestingly, some leopard individuals are known to become specialists in a particular prey, feeding almost exclusively on a single species. Within the studied period, specimens show little variability in their $\delta^{66}Zn$ but a larger range in $\delta^{13}C$ values at Tam Hay Marklot (**fig. S21**). This could suggest predation on the same species (given the narrow range of $\delta^{66}Zn$ values) that forage in different habitats, as exhibited by the $\delta^{13}C$ values, such as the large-sized bovids or caprines. The panthers' $\delta^{18}O$ values decrease across sites from older to younger periods.

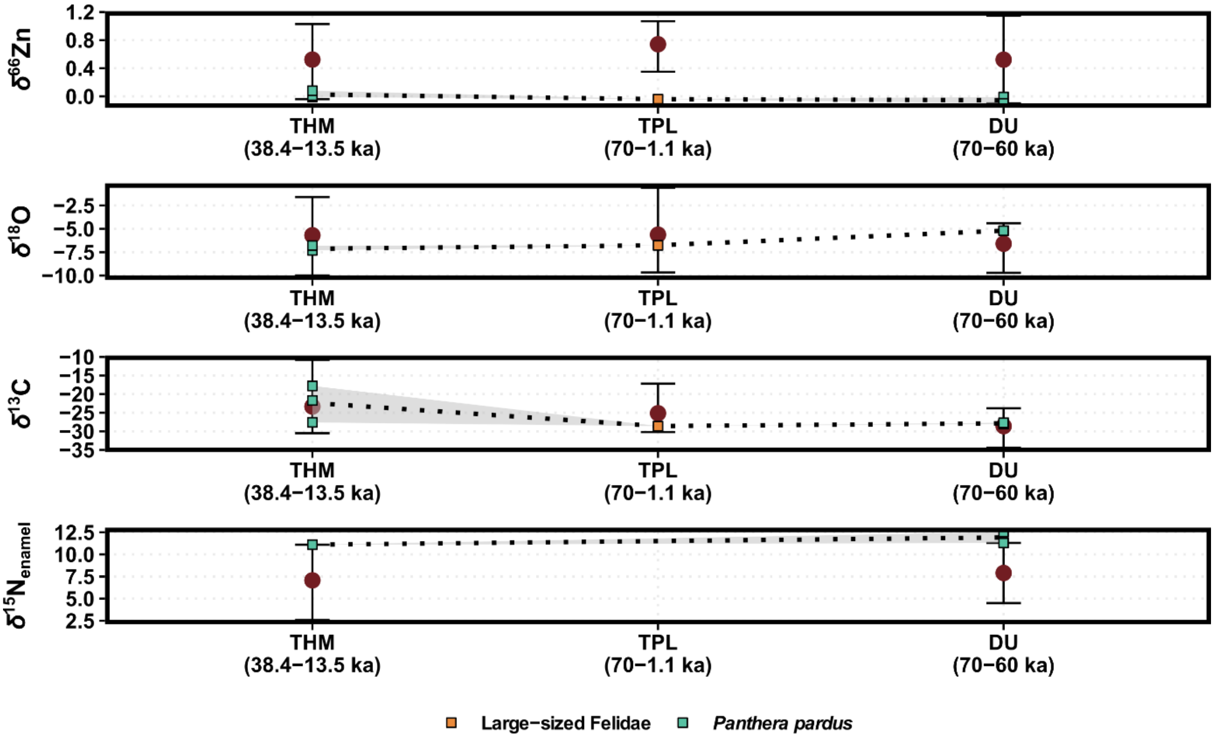

Figure S21. The $\delta^{15}N_{enamel}$ (‰ AIR), $\delta^{66}Zn$ (‰ JMC-Lyon), $\delta^{13}C$ (‰ VPDB), and $\delta^{18}O$ (‰ VPDB) values of leopards (*Panthera pardus*) between sites. The $\delta^{13}C$ values are converted to those of the food web's primary carbon sources for better comparability between species, and both $\delta^{13}C$ and $\delta^{18}O$ values are taken from Bacon et al. (*10*) and Bourgon et al. (*27*, *28*). The $\delta^{66}Zn$ values from Tam Hay Marklot are taken from Bourgon et al. (*27*), and those from Tam Pà Ling from Bourgon et al. (*28*). The $\delta^{15}N_{enamel}$ values from Tam Hay Marklot are taken from Leichliter et al. (*19*). The whiskers represent the full range of values for each site, and the red dots correspond to the mean of each site. The dotted line is the moving average of leopards' values between sites, while the shaded area delimits the maximum and minimum values for leopards found between two sites. The specimen from Tam Pà Ling (large-sized Felidae) is not identified at the species level and thus present a certain degree of uncertainty, as it could equally be a tiger (*Panthera tigris*). Tam Hay Marklot is denoted with the abbreviation THM, Tam Pà Ling with TPL, and, finally, Duoi U'Oi with DU.

Panthera tigris

Tigers (*Panthera tigris*) inhabit tropical, subtropical, and temperate forests of Southeast Asia, and are a generalist species found even in Palearctic regions (*158*). Like the leopards, their primary habitat requirement is a sufficient prey base, though they tend to favor boars and deer of various species (*159*). Within the range of carnivores, the $\delta^{66}Zn$ values of tigers vary comparatively more than those of leopards, and have higher $\delta^{13}C$ values at Tam Hay Marklot compared to the low values at other sites (**fig. S22**). The $\delta^{13}C$ values align well with those of boars, suggesting they may have been the tigers' preferred prey, similar to modern populations. Compared to many species, the tigers' $\delta^{18}O$ values follow an opposite trend relative to that of the site: they exhibit lower values in sites with comparatively higher values, and vice versa.

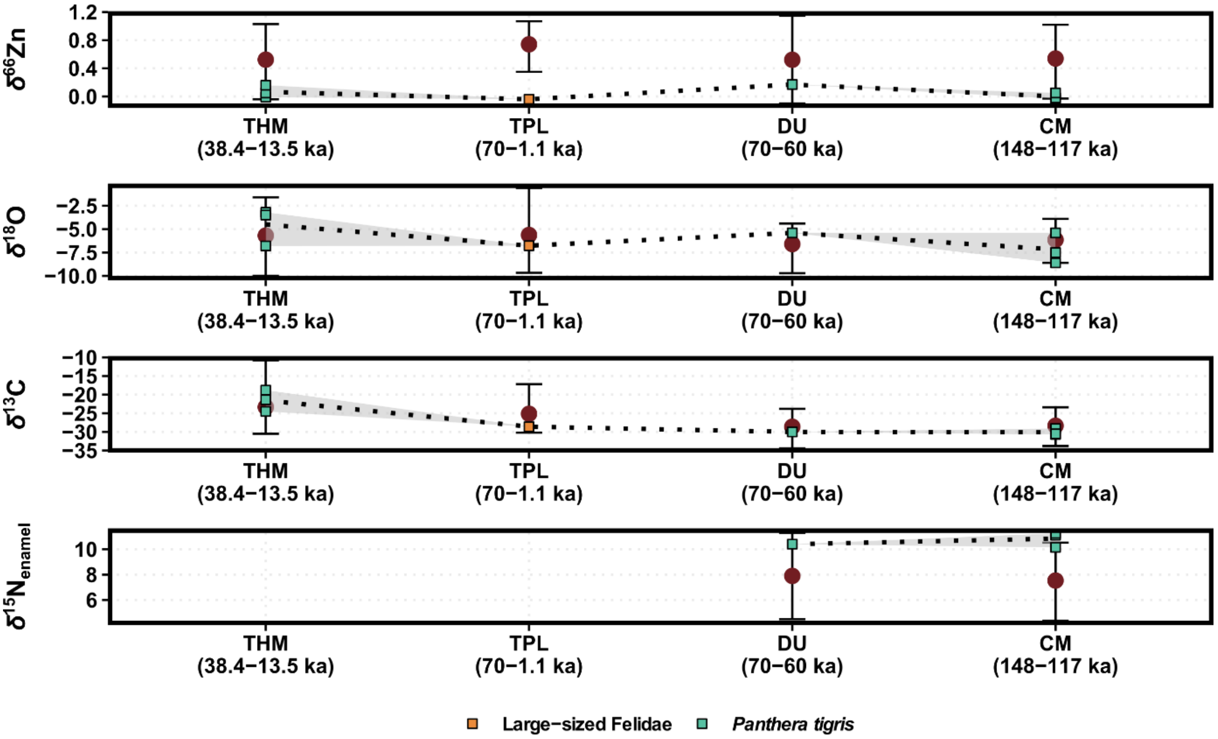

Figure S22. The $\delta^{15}N_{enamel}$ (‰ AIR), $\delta^{66}Zn$ (‰ JMC-Lyon), $\delta^{13}C$ (‰ VPDB), and $\delta^{18}O$ (‰ VPDB) values of tigers (*Panthera tigris*) between sites. The $\delta^{13}C$ values are converted to those of the food web's primary carbon sources for better comparability between species, and both $\delta^{13}C$ and $\delta^{18}O$ values are taken from Bacon et al. (*10*) and Bourgon et al. (*27*, *28*). The $\delta^{66}Zn$ values from Tam Hay Marklot are taken from Bourgon et al. (*27*), and those from Tam Pà Ling from Bourgon et al. (*28*). The whiskers represent the full range of values for each site, and the red dots correspond to the mean of each site. The dotted line is the moving average of tigers' values between sites, while the shaded area delimits the maximum and minimum values for tigers found between two sites. The specimen from Tam Pà Ling (large-sized Felidae) is not identified at the species level and thus present a certain degree of uncertainty, as it could equally be a leopard (*Panthera pardus*). Tam Hay Marklot is denoted with the abbreviation THM, Tam Pà Ling with TPL, Duoi U'Oi with DU, and, finally, Coc Muoi with CM.

Tapirus indicus

Today, Malay tapirs (*Tapirus indicus*) occupy tropical moist forest areas and wetland areas. It has been suggested that a more seasonal climate and harsher dry season could be the driver for this species' extirpation in some regions of Southeast Asia, including Laos and Vietnam (*160*). Within our dataset, the $\delta^{13}C$ and $\delta^{18}O$ values of tapirs follow the climatic fluctuations associated with each site (**fig. S23**); i.e., while their absolute $\delta^{13}C$ values change, these species' position relative to others remains similar in each site. The low $\delta^{13}C$ values specifically indicate foraging in the understory of a canopy forest (*13*). This, in turn, suggests remaining and foraging within a gradually changing habitat whose $\delta^{13}C$ values change as the canopy effect lessens with the progress of deciduous/mixed dry forests at the expense of lowland rainforests. Accordingly, $\delta^{66}Zn$ and $\delta^{15}N_{enamel}$ values mostly remain similar across sites (**fig. S23**). The relatively low values for both tracers, compared to other herbivorous taxa, suggest a diet likely including fruits and/or young leaves and shoots (*49, 52*), a dietary behavior consistent with modern observation (*161*). Overall, the combination of specific plant organs (e.g., young leaves, fruits, etc.) and/or species within the understory potentially appears to be the main driver in the tapir's dietary niche.

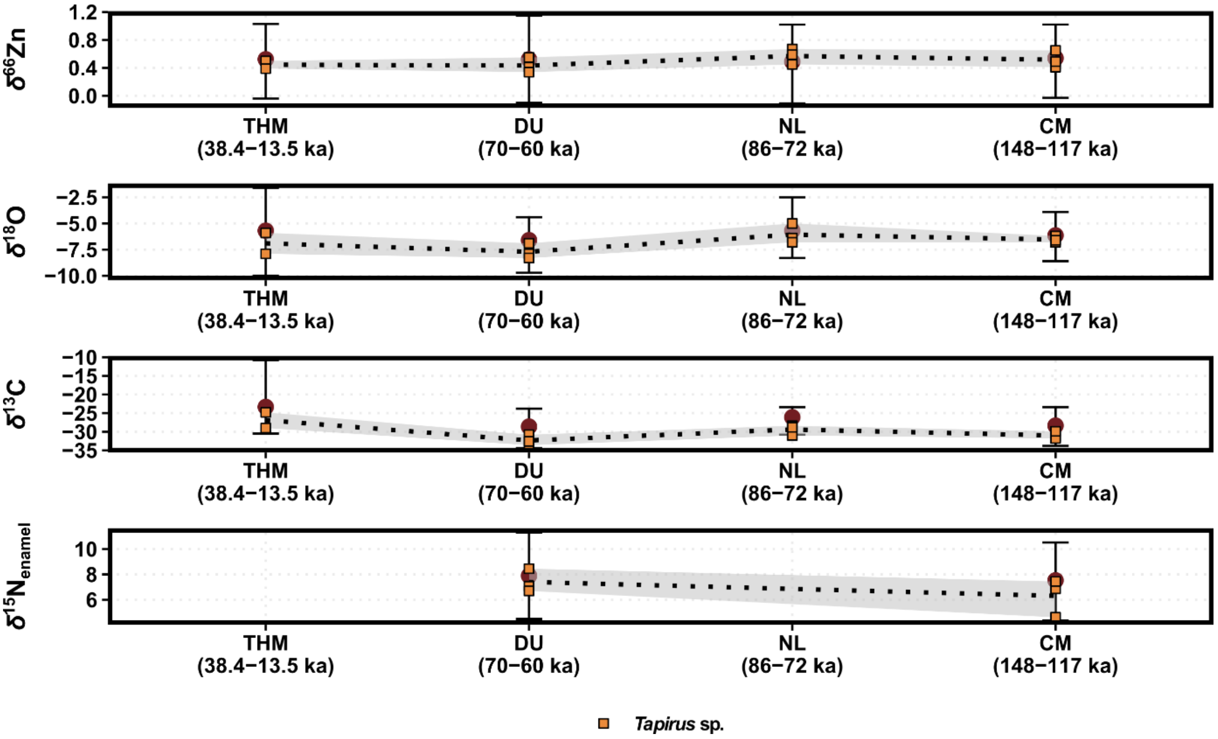

Figure S23. The $\delta^{15}N_{enamel}$ (‰ AIR), $\delta^{66}Zn$ (‰ JMC-Lyon), $\delta^{13}C$ (‰ VPDB), and $\delta^{18}O$ (‰ VPDB) values of Malay tapirs (*Tapirus indicus*) between sites. The $\delta^{13}C$ values are converted to those of the food web's primary carbon sources for better comparability between species, and both $\delta^{13}C$ and $\delta^{18}O$ values are taken from Bacon et al. (*10*, *46*) and Bourgon et al. (*27*, *28*). The $\delta^{66}Zn$ values from Tam Hay Marklot are taken from Bourgon et al. (*27*), and those from Nam Lot from Bourgon et al. (*28*). The whiskers represent the full range of values for each site, and the red dots correspond to the mean of each site. The dotted line is the moving average of tapirs' values between sites, while the shaded area delimits the maximum and minimum values for tapirs found between two sites. Tam Hay Marklot is denoted with the abbreviation THM, Duoi U'Oi with DU, Nam Lot with NL, and finally, Coc Muoi with CM.

Supplementary Material – Text 3: Data normalization and statistical analyses robustness

Even in their simplest form, multi-isotope studies are inherently complex, as numerous factors must be carefully accounted for, and the raw data are rarely suitable for direct comparison. This complexity is further amplified by confounding variables such as differences in locality, chronology, and faunal composition. When multiple sites are included in the analysis, two main concerns emerge (though not exclusively): (1) ensuring comparability between different isotopic proxies, each with distinct baseline behaviors and interpretive frameworks, and (2) achieving meaningful comparisons between sites, which may vary in environmental context, temporal resolution, and taxonomic representation.

In multi-isotope analyses, a key methodological challenge lies in the comparability between isotopic proxies, each of which reflects distinct aspects of an organism's ecology and operates, for example, on differing baseline dynamics, spatial scales, and biochemical pathways. Moreover, each isotope system is measured on a different scale and exhibits differing degrees of variability within and between taxa, making direct visualization and statistical integration challenging without appropriate normalization or standardization. As such, care must be taken not only in the interpretation of individual proxies but also in the frameworks used to integrate them, as apparent patterns of ecological differentiation may emerge or be obscured depending on the analytical treatment of each isotopic axis.

Because isotopic breadth was the primary focus of this study, data normalization was essential to evaluate the relative contribution of each proxy. Several normalization methods exist, each with specific strengths and limitations, and careful consideration is required. In our case, z-score normalization provided a key advantage by enabling comparability across isotopic proxies that differ in scale and variability. Unlike min-max scaling, which compresses values into a fixed range and is highly sensitive to outliers, z-scoring preserves the relative distribution by centering each variable on its mean and scaling by its standard deviation. This approach is particularly useful when integrating proxies with differing units or baseline variability, allowing for combined analyses without distorting ecological signals, especially important in ecological, paleoecological, and archaeological contexts.

Moreover, z-score normalization is a widely used statistical method that standardizes data since it removes units and places all variables on a common scale, which is particularly important when comparing or combining variables that differ in magnitude, range, or measurement units. It prevents any single variable from disproportionately influencing model outcomes, improves interpretability of effect sizes, and enhances numerical stability in linear and multivariate analyses. Z-scoring also helps meet assumptions such as homoscedasticity and comparability of coefficients, making it a valuable step in preparing complex datasets for robust statistical inference.

When applying this approach, it is important to consider that z-score normalization can be applied either across the entire dataset or within predefined groups (e.g., by site). Standardizing across the whole dataset assumes a shared baseline and uses the global mean and standard deviation, which may be suitable in some contexts. However, when group means or variances differ, this approach risks flattening or inflating group-specific variation, potentially obscuring ecologically meaningful differences.

By contrast, z-scoring within groups (e.g., by site) preserves each group's internal structure by standardizing values relative to that group's mean and standard deviation. This is particularly useful when baseline shifts between groups are expected (due to environmental or temporal variation, for example), as it corrects for such offsets and enables fairer comparison of variability across sites, but group-wise z-scoring is not without limitations. A key concern is that it can inadvertently amplify small differences in groups with naturally low variability: when the standard deviation is small, even minor absolute differences can translate into large z-scores. In our case, this may overstate variability in taxa from more homogeneous environments, potentially skewing interpretations of ecological flexibility or niche breadth. However, this effect applies equally to all taxa within a given group and does not introduce systematic bias in favor of specific taxonomic or conservation groups. Nonetheless, it represents an important caveat when interpreting results across sites with differing intrinsic variability.

Although arguably unnecessary, a normalization procedure was also applied to Coc Muoi's δ^{66}Zn values in this study (**fig. S24**). While nearly all variables are consistent across sites (including the absolute isotopic ranges within food webs and dietary groups, the faunal composition, and the trophic spacing), Coc Muoi exhibited a slight but consistent baseline shift in δ^{66}Zn values across taxa relative to the other localities. While this would be corrected through the z-score transformation described above, we felt it important, given the relative novelty of δ^{66}Zn as a dietary proxy, to also present the values on their native scale. To that end, we normalized Coc Muoi's δ^{66}Zn values relative to the combined mean of the three other comparable sites (Pà Hang, Tam Hay Marklot, and Duoi U'Oi). It is worth noting that this approach is possible and justified by the strong comparability across these localities in terms of faunal composition, environmental context, and isotopic structure, including trophic spacing and absolute ranges.

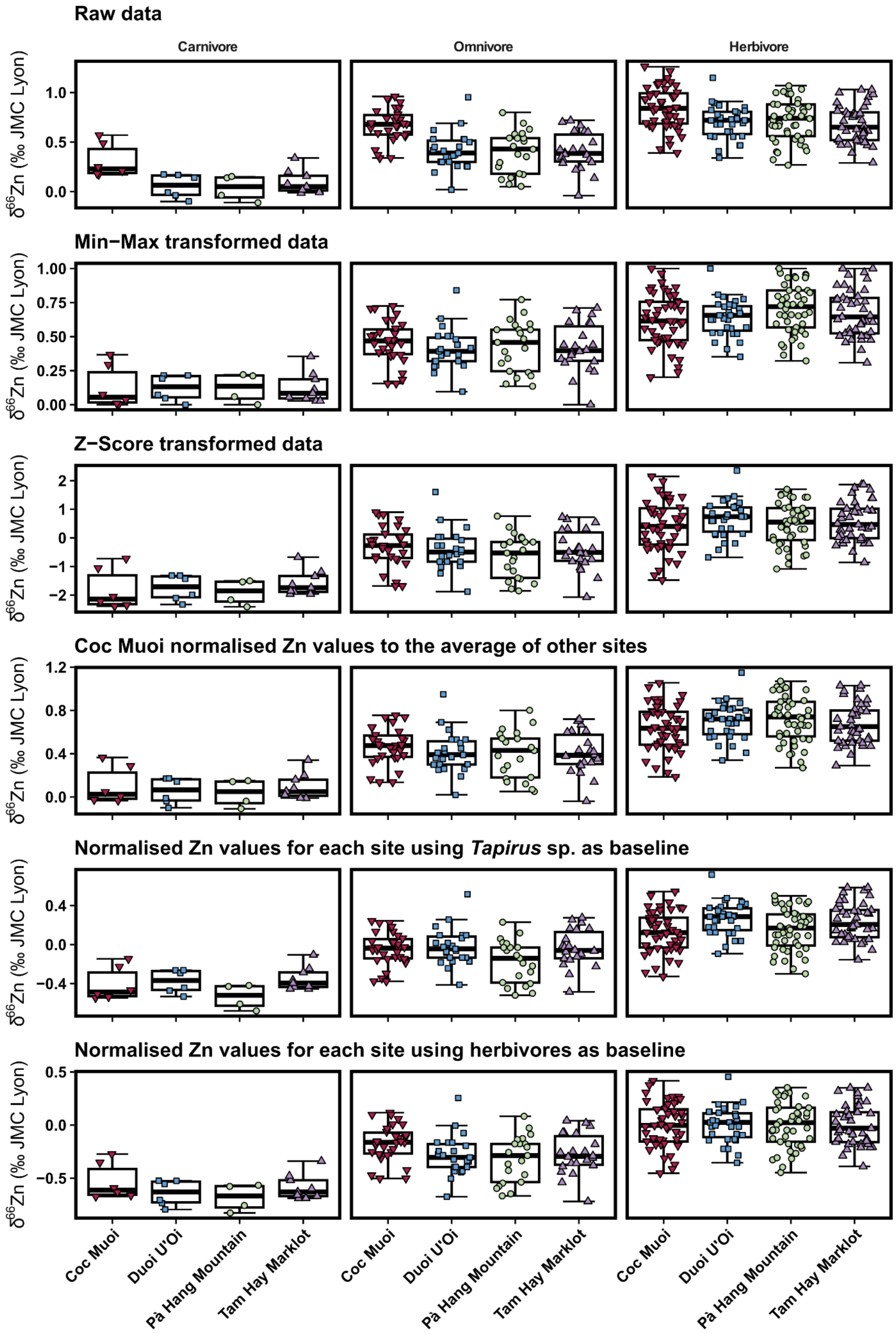

Figure S24. Box and whisker plots of δ^{66}Zn values (‰ JMC-Lyon) in tooth enamel of Pleistocene Southeast Asian mammals, and compared using various normalization methods. Each of colors and symbols represents specimens from different sites:

upside-down burgundy triangles for Coc Muoi, blue squares for Duoi U'Oi, green circles for Pà Hang Mountain (which include Tam Pà Ling and Nam Lot), and purple triangles for Tam Hay Marklot. "Z-score" refers to the standard-score method ((x-μ)/σ). The δ^{66}Zn values from Tam Hay Marklot are taken from Bourgon et al. (27) and those from Tam Pà Ling and Nam Lot from Bourgon et al. (28). The boxes represent the 25th–75th percentiles, with the median represented by a bold horizontal line.

In contrast, site-specific normalization (e.g., using the local mean of a shared taxon or dietary group) risks compressing or expanding trophic spacing in ways that obscure meaningful ecological variation (**fig. S24**). Because the δ^{66}Zn signal reflects not only trophic level (i.e., plant vs. animal consumption) but also plant type (e.g., leaves vs. grasses), preserving the relative positioning of taxa is essential for interpreting dietary breadth and shifts. Normalizing each site independently can distort these relationships by adjusting each site's trophic structure in isolation, thereby reducing the interpretability of between-site comparisons. This is particularly problematic in contexts where intra-taxon dietary flexibility exists, even across similar environmental settings, as this could introduce artificial distortion because each site's scaling factor would differ, even if the variation seems minor. In such cases, small shifts in baseline taxa (e.g., slightly higher or lower δ^{66}Zn values for *Tapirus* at a given site) can shift the entire site's isotopic structure up or down, altering the perceived spacing between taxa (**fig. S24**). As a result, taxon- or diet-specific normalization can disrupt otherwise consistent trophic patterns (such as those observed at Coc Muoi) and introduce artificial variation, which we view as especially problematic for inter-site analyses. By contrast, the normalization strategy we employed avoids these distortions, maintains the ecological signal encoded in trophic spacing, and limits data transformation to the one site where a modest baseline correction was warranted. It thereby ensures a more robust framework for inter-site comparisons while preserving interpretability in both transformed and native scales.

In addition to ensuring comparability between different isotopic proxies, data normalization also facilitates meaningful comparisons between sites. In the context of this study, this is particularly relevant for δ^{18}O values. As previously discussed, δ^{66}Zn values are largely consistent across taxa and sites, with the exception of Coc Muoi, whose values were normalized to align with the others. Similarly, $\delta^{15}N_{enamel}$ values at Coc Muoi and Duoi U'Oi do not exhibit baseline offsets, showing comparable ranges and consistent trophic spacing. For δ^{13}C, the range is constrained by the well-documented C_3–C_4 plant distinction. While some sites may exhibit broader or narrower δ^{13}C ranges, this reflects ecological differences in plant community composition rather than baseline issues. By contrast, δ^{18}O values are more directly influenced by environmental variables such as climate, elevation, and vegetation cover.

In this study, all sites are located within a narrow latitudinal belt (between 20° and 23° N) spanning northern Laos and Vietnam. This geographic clustering helps minimize environmental variation due to latitudinal clines (e.g., temperature, rainfall amount and seasonality, and proximity to the coast),

thereby enabling more rigorous inter-site comparisons. The primary abiotic difference among sites is elevation, which ranges from lowland alluvial plains (e.g., Duoi U'Oi) to mid-elevation mountain environments (~1,120 m at Pà Hang Mountain). Each site comprises a mosaic of habitats whose proportions reflect both ecological heterogeneity and the site's chronological context, with varying representations of closed forest canopy, intermediate woodland/rainforest, and open environments typical of tropical wet and humid subtropical climates.

As the different sites encompass different ages, distinct climatic conditions will be the primary cause of variability in $\delta^{18}O$ values. Without normalization, comparing raw isotope ranges across sites would conflate ecological response with local abiotic conditions. For example, absolute $\delta^{18}O$ values can differ between sites due to rainfall regime (which is somewhat expected given the different ages of the sites), but the ecological meaning of variation (e.g., whether animals shift their water sources, diet, or canopy height use) should be evaluated relative to the site's own environmental context. By centering each site's isotope data, we thus preserve local ecological signals while minimizing confounding from non-behavioral baseline shifts.

While only $\delta^{18}O$ and $\delta^{66}Zn$ values strictly required normalization to enable coherent intra- and inter-site comparisons, this study examines isotopic breadth across all tracers and localities. Thus, a consistent normalization approach was adopted. For example, using Δ-values (i.e., max–min ranges) would retain the proxies' native scales and yield broadly similar patterns; namely, that extant taxa exhibit greater isotopic breadth than extirpated or extinct ones (**fig. S25** and **tables S11** and **S12**). However, Δ-values would also amplify inter-site variability driven by underlying environmental baselines rather than ecological behavior. This is particularly problematic for $\delta^{18}O$, where baseline values can differ markedly due to local climatic conditions, elevation, or vegetation, potentially confounding ecological interpretations. Unlike z-score transformation (which may inadvertently magnify small variations in sites with naturally narrow isotopic ranges but does so uniformly across taxa), inter-site comparison of raw Δ-values does not affect all groups equally. Instead, it introduces systematic bias by exaggerating the variability of taxa that already exhibit wide isotopic ranges, often aligning with specific taxonomic or conservation statuses. Lastly, even if one were to apply a form of centering across all proxies to mitigate baseline differences, data normalization would still be necessary to perform robust statistical analyses and ensure comparability across dimensions. Moreover, model fit statistics clearly favor the z-score-based model (**table S13**), which showed substantially lower AIC, higher marginal and conditional R^2 values, and lower residual variance compared to the model using raw Δ-values. While both models yielded broadly consistent results, with the direction and significance of the main predictor (i.e., reduced isotopic breadth in extirpated taxa) remaining stable, the z-score model offers improved performance and interpretability.

Predictor	Model	df	Estimate	SE	*t*	*p*	Lower CI	Upper CI
**Intercept**	Main LMM	16	1.52	0.25	6.22	< 0.001	1.07	1.98
	Δ LMM	20	2.88	0.71	4.04	0.001	1.56	4.20
	Log LMM	16	0.35	0.19	1.83	0.087	0.00	0.71
	Outlier	15	1.46	0.22	6.70	< 0.001	1.06	1.87
	All LMM	19	1.55	0.26	5.88	< 0.001	1.07	2.04
	!na.N LMM	13	1.35	0.35	3.86	0.002	0.71	1.99
	no N LMM	16	1.65	0.29	5.70	< 0.001	1.12	2.19
**Status: Extirpated/Extinct(1)**	Main LMM	16	-0.61	0.21	-2.94	0.010	-1.00	-0.23
	Δ LMM	17	-1.31	0.58	-2.28	0.036	-2.38	-0.25
	Log LMM	16	-0.51	0.16	-3.08	0.007	-0.81	-0.20
	Outlier	15	-0.49	0.19	-2.57	0.021	-0.85	-0.14
	All LMM	16	-0.66	0.22	-3.08	0.007	-1.06	-0.27
	!na.N LMM	13	-0.53	0.27	-1.97	0.070	-1.03	-0.04
	no N LMM	16	-0.74	0.25	-3.02	0.008	-1.20	-0.29
**Diet: Omnivore(2)**	Main LMM	16	0.03	0.30	0.09	0.933	-0.53	0.59
	Δ LMM	18	-0.39	0.85	-0.46	0.652	-1.96	1.18
	Log LMM	16	0.02	0.24	0.08	0.940	-0.42	0.46
	Outlier	15	0.06	0.27	0.23	0.822	-0.43	0.56
	All LMM	17	0.01	0.32	0.04	0.970	0.58	0.60
	!na.N LMM	13	0.27	0.40	0.69	0.505	-0.45	1.00
	no N LMM	16	-0.07	0.36	-0.19	0.853	-0.73	0.59
**Diet: Herbivore(2)**	Main LMM	16	0.27	0.26	1.05	0.308	-0.20	0.75
	Δ LMM	19	0.46	0.74	0.62	0.542	-0.91	1.84
	Log LMM	16	0.09	0.20	0.42	0.681	-0.29	0.46
	Outlier	15	0.18	0.23	0.76	0.460	-0.25	0.61
	All LMM	18	0.31	0.28	1.10	0.285	-0.21	0.82
	!na.N LMM	13	0.33	0.35	0.93	0.371	-0.31	0.96
	no N LMM	16	0.28	0.31	0.90	0.382	-0.29	0.84

(1) Dummy coded with "Extant" as the reference category.
(2) Dummy coded with "Carnivore" as the reference category.

Table S11. Summary of fixed effects across linear mixed models testing isotopic range variation by status and diet. Summary of fixed effect estimates from various linear mixed models (LMM) assessing the influence of conservation status and dietary classification on isotopic breadth. Models include the main z-score normalized model ("Main LMM"), a model using raw Δ-values ("Δ LMM"), a log-transformed model ("Log LMM"), a model excluding Large-sized Bovidae ("Outlier"), a model including all available isotope ranges ("All LMM"), a model excluding specimens with missing $\delta^{15}N_{enamel}$ values ("!na.N LMM"), and a model entirely excluding the $\delta^{15}N_{enamel}$ proxy ("no N LMM"). All models include taxon as a random intercept.

Random Effect	Model	Variance	σ	Lower CI	Upper CI
**Taxon**	Main LMM	0.074	0.27	0.00	0.44
	Δ LMM	0.826	0.91	0.17	1.31
	Log LMM	0.048	0.22	0.00	0.35
	Outlier	0.063	0.25	0.00	0.40
	All LMM	0.146	0.38	0.18	0.52
	!na.N LMM	0.144	0.38	0.00	0.56
	no N LMM	0.093	0.31	0.00	0.52
**Residuals**	Main LMM	0.491	0.70	0.59	0.84
	Δ LMM	9.443	3.07	2.84	3.34
	Log LMM	0.299	0.55	0.46	0.66
	Outlier	0.366	0.60	0.51	0.73
	All LMM	0.896	0.95	0.87	1.03
	!na.N LMM	0.506	0.71	0.59	0.88
	no N LMM	0.547	0.74	0.60	0.92

Table S12. Random effect variance and residual estimates across model variants. Variance (and associated standard deviation, σ) of the random effect (taxon-level intercepts) and residuals for each linear mixed-effects model (LMM) variant used in sensitivity testing. Confidence intervals (95%) are based on profile likelihood estimates. Models include the main z-score normalized model ("Main LMM"), a model using raw Δ-values ("Δ LMM"), a log-transformed model ("Log LMM"), a model excluding Large-sized Bovidae ("Outlier"), a model including all available isotope ranges ("All LMM"), a model excluding specimens with missing $\delta^{15}N_{enamel}$ values ("!na.N LMM"), and a model entirely excluding the $\delta^{15}N_{enamel}$ proxy ("no N LMM"). While the main LMM (z-scored data) displays relatively low between-taxon variance and residual error, alternative models, such as the Δ-value model, show markedly higher unexplained variance, especially in residuals. This supports the choice of the z-score model for improved model fit and interpretability.

Model	AIC	R^2 conditional	R^2 marginal
Main LMM	193.58	0.26	0.14
Δ LMM	1552.587	0.12	0.04
Log LMM	156.32	0.27	0.15
Outlier	164.09	0.25	0.12
All LMM	842.76	0.22	0.09
!na.n LMM	171.68	0.32	0.13
no N LMM	155.03	0.30	0.18

Table S13. Model fit statistics across LMM variants used in sensitivity analyses. Model comparison table showing Akaike Information Criterion (AIC), conditional R^2 (variance explained by both fixed and random effects), and marginal R^2 (variance explained by fixed effects only) across linear mixed-effects model (LMM) variants. Models include the main z-score normalized model ("Main LMM"), a model using raw Δ-values ("Δ LMM"), a log-transformed model ("Log LMM"), a model excluding Large-sized Bovidae ("Outlier"), a model including all available isotope ranges ("All LMM"), a model excluding specimens with missing $\delta^{15}N_{enamel}$ values ("!na.N LMM"), and a model entirely excluding the $\delta^{15}N_{enamel}$ proxy ("no N LMM"). The z-scored model ("Main LMM") demonstrates a favorable balance of model fit and explanatory power. Although the log-transformed model ("Log LMM") shows slightly improved fit (lower AIC and marginal R^2), the differences are minor, and core conclusions remain stable. The Δ-value model exhibits a markedly poorer fit (AIC > 1500), reinforcing the rationale for preferring z-scored data in the main analysis.

Even after careful normalization of the data, datasets are often not perfectly equivalent and ready for statistical analyses and may require further transformation. Notably, ranges (i.e., including isotopic ranges calculated in the current study from z-scored data) are always non-negative by definition, regardless of the distribution of the underlying data, and will thus tend to be right-skewed (i.e., follow a log-normal distribution, although not systematically). Such LMM models may produce biased estimates, inflated Type I error rates, or misleading inferences when the response variable follows a log-normal distribution. While it is common to log-transform log-normal data to approximate normality and stabilize variance, this transformation alters the scale and interpretability of the response variable. Moreover, log-transforming already standardized or ratio-based data (such as z-scored ranges or proportions) can complicate interpretation and introduce new artifacts, especially when values are close to zero.

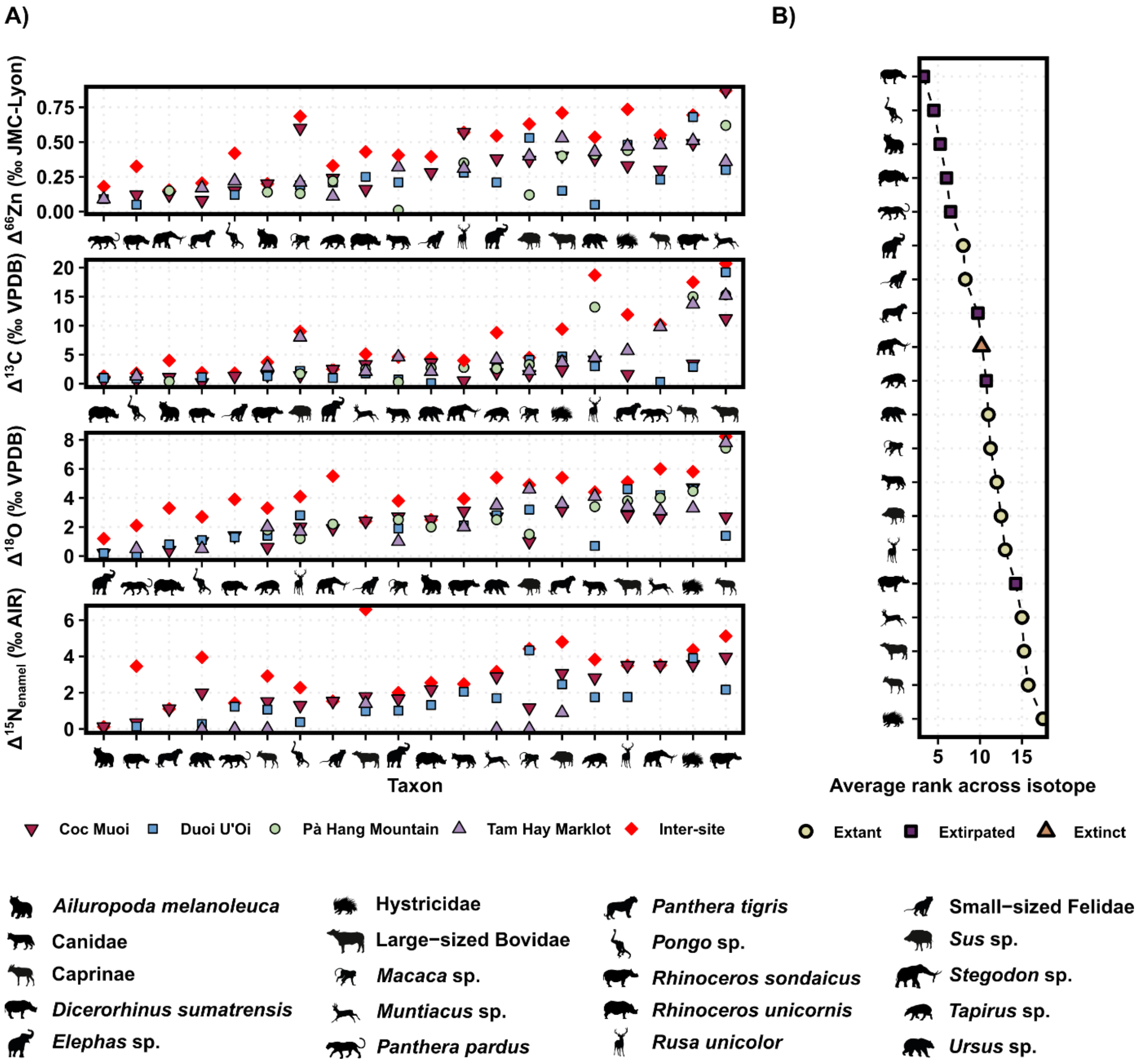

Figure S25. Isotopic breadth across proxies and taxa using native scale ranges (Δ-values). Dot plots showing the isotopic range (Δ; max–min) for each taxon across four isotopic proxies (A): $\delta^{13}C$ (VPDB), $\delta^{15}N_{enamel}$ (AIR), $\delta^{18}O$ (VPDB), and $\delta^{66}Zn$ (JMC Lyon). Each subplot corresponds to a different isotope proxy, with taxa ordered in ascending range specific to that proxy. Each data point represents the range value of a given taxon at one locality. Animal silhouettes are used on the x-axis to aid

visual clarity, with corresponding taxon names listed in the legend. Sites are distinguished by shape and color: upside-down burgundy triangles for Coc Muoi (148–117 ka), blue squares for Duoi U'Oi (70–60 ka), green circles for Pà Hang Mountain (Tam Pà Ling and Nam Lot, 70–1.1 ka and 86–72 ka, respectively), purple triangles for Tam Hay Marklot (38.4–13.5 ka), and red diamonds for inter-site comparisons. Cumulative rank-based comparison of isotopic ranges across proxies (B). For each taxon, ranks (1 = smallest range; 20 = largest range) were assigned within each proxy based on their position in Panel A. These four ranks were then summed, and taxa were ordered in ascending total rank (i.e., from smallest to largest overall isotopic breadth). Taxa are plotted using distinct colors and shapes based on conservation status: extant (light green circles), extirpated (purple squares), and extinct (orange triangles). The $\delta^{13}C$ values were converted to those of the food web's primary carbon sources for better comparability between taxa (see **Supplementary Material Text 1**). Both $\delta^{13}C$ and $\delta^{18}O$ values are taken from Bacon et al. (10, 46) and Bourgon et al. (27, 28). The $\delta^{66}Zn$ values from Tam Hay Marklot are taken from Bourgon et al. (27), and those from Tam Pà Ling and Nam Lot from Bourgon et al. (28). The $\delta^{15}N_{enamel}$ values from Tam Hay Marklot are taken from Leichliter et al. (19). The $\delta^{66}Zn$ values of Coc Muoi are normalized to the average $\delta^{66}Zn$ value of all other Southeast Asian sites (i.e., Tam Hay Marklot, Pà Hang Mountain, and Duoi U'Oi).

For these reasons, we prioritized using untransformed ranges calculated from z-scored data for our linear mixed model analyses, while also conducting a log-transformed model to assess the sensitivity of our results. The log-transformed model yielded improved fit statistics (**table S13**), including a notably lower AIC (ΔAIC ≈ 37) and slightly higher marginal and conditional R^2 values (0.152 and 0.269) compared to the original model (0.144 and 0.257). Posterior predictive checks similarly showed better alignment between observed and predicted distributions. Nevertheless, some diagnostic issues (such as mild non-linearity and heteroscedasticity) persisted, particularly at the extremes of the fitted range. Given that log-transforming z-scored ranges reduces ecological interpretability and that the overall conclusions remained unchanged (with the direction and significance of the main predictor, namely the reduced isotopic range in extirpated taxa, remaining stable; **fig. S26** and **tables S11** and **S12**), we opted to retain the untransformed model in the main text.

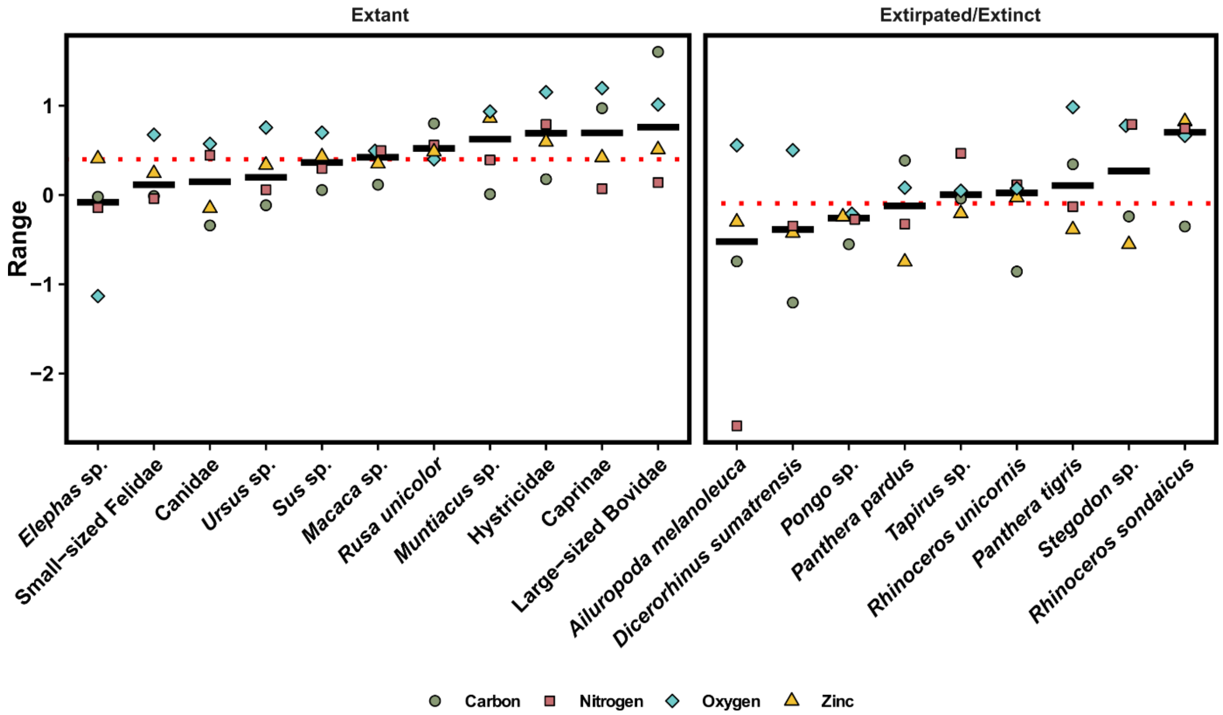

Figure S26. Average isotopic range after log transformation of $\delta^{15}N_{enamel}$ (‰ AIR), $\delta^{66}Zn$ (‰ JMC-Lyon), $\delta^{13}C$ (‰ VPDB), and $\delta^{18}O$ (‰ VPDB) of extant, locally-extirpated, and extinct taxa for the studied period. All isotopic values are standard-score

transformed to allow direct comparison between the different isotope systems, and then log transformed. Taxa found at only a single site were excluded, as their isotopic ranges only capture intra-site variability rather than temporal changes across sites. The range of every isotopic system was calculated for each taxon at each site (Tam Hay Marklot, Pà Hang Mountain, Duoi U'Oi, and Coc Muoi) and across sites (i.e., absolute maximum - absolute minimum), and then averaged into a single value for each isotopic system. The ranges calculated from a single individual (i.e., *n* = 1 for any given taxa at a given site) were excluded. The δ^{13}C values were converted to those of the food web's primary carbon sources for better comparability between taxa (see **Supplementary Material Text 1**). Both δ^{13}C and δ^{18}O values are taken from Bacon et al. *(*10, 46*)* and Bourgon et al. *(*27, 28*)*. The δ^{66}Zn values from Tam Hay Marklot are taken from Bourgon et al. *(*27*)*, and those from Tam Pà Ling and Nam Lot from Bourgon et al. *(*28*)*. The $\delta^{15}N_{enamel}$ values from Tam Hay Marklot are taken from Leichliter et al. *(*19*)*. The δ^{66}Zn values of Coc Muoi are normalized to the average δ^{66}Zn value of all other Southeast Asian sites (i.e., Tam Hay Marklot, Pà Hang Mountain, and Duoi U'Oi). The median range of each taxa is represented by a bold horizontal line, and the red dotted line represents the average range for each panel (extant, locally-extirpated, and extinct).

Lastly, although not directly related to data normalization, we conducted several additional sensitivity tests on the main z-scored dataset to assess the robustness of our findings. In particular, two model iterations were run to evaluate the potential impact of having an incomplete multi-dimensional dataset, as $\delta^{15}N_{enamel}$ values are not available for all specimens (**figs. S27** and **S28**, and **tables S11** and **S12**). One model excluded $\delta^{15}N_{enamel}$ entirely, while another excluded only those specimens lacking $\delta^{15}N_{enamel}$ data.

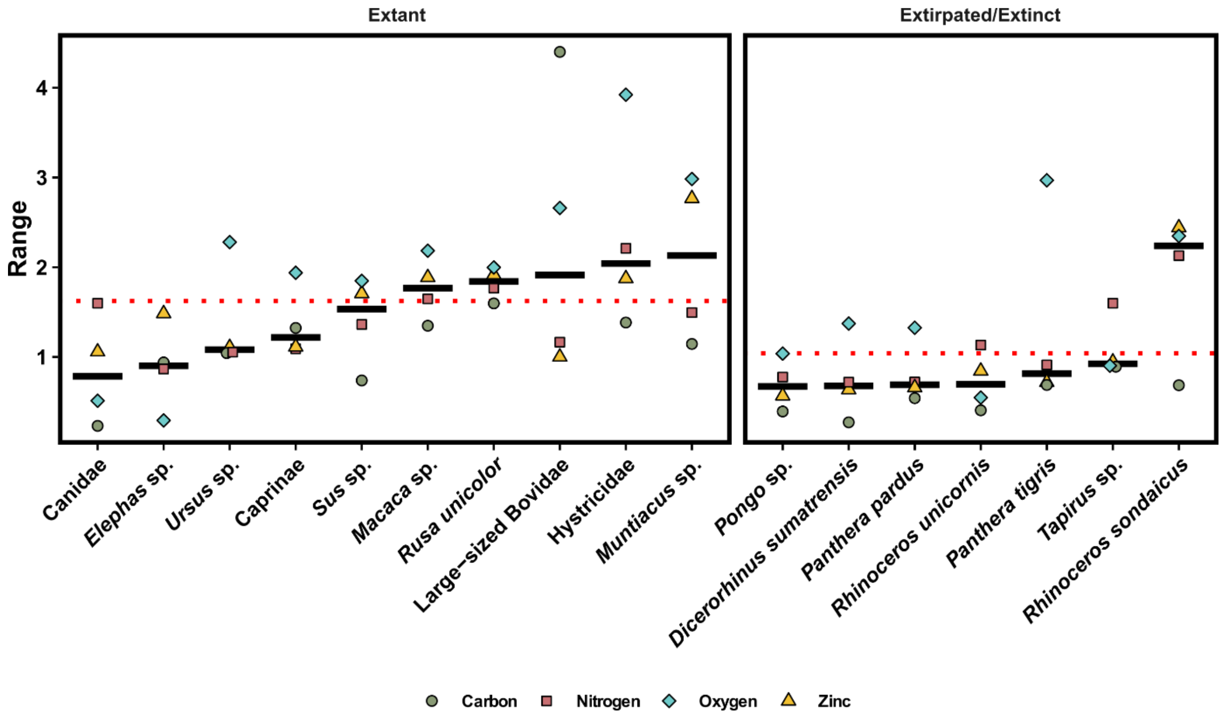

Figure S27. Average isotopic range of $\delta^{15}N_{enamel}$ (‰ AIR), δ^{66}Zn (‰ JMC-Lyon), δ^{13}C (‰ VPDB), and δ^{18}O (‰ VPDB) of extant, locally-extirpated, and extinct taxa for the studied period with the exclusion of specimen lacking associated $\delta^{15}N_{enamel}$ values. All isotopic values are standard-score transformed to allow direct comparison between the different isotope systems. Taxa found at only a single site were excluded, as their isotopic ranges only capture intra-site variability rather than temporal changes across sites, and taxa lacking $\delta^{15}N_{enamel}$ values were also excluded. The range of every isotopic system was calculated for each taxon at each site (Tam Hay Marklot, Pà Hang Mountain, Duoi U'Oi, and Coc Muoi) and across sites (i.e., absolute maximum - absolute minimum), and then averaged into a single value for each isotopic system. The ranges calculated from a single individual (i.e., *n* = 1 for any given taxa at a given site) were excluded. The δ^{13}C values were converted to those of the food web's primary carbon sources for better comparability between taxa (see **Supplementary Material Text 1**). Both δ^{13}C and δ^{18}O values are taken from Bacon et al. *(*10, 46*)* and Bourgon et al. *(*27, 28*)*. The δ^{66}Zn values from Tam Hay Marklot are taken from Bourgon et al. *(*27*)*, and those from Tam Pà Ling and Nam Lot from Bourgon et al. *(*28*)*. The $\delta^{15}N_{enamel}$ values from Tam Hay Marklot are taken from Leichliter et al. *(*19*)*. The δ^{66}Zn values of Coc Muoi are normalized to the average δ^{66}Zn value

of all other Southeast Asian sites (i.e., Tam Hay Marklot, Pà Hang Mountain, and Duoi U'Oi). The median range of each taxa is represented by a bold horizontal line, and the red dotted line represents the average range for each panel (extant, locally-extirpated, and extinct).

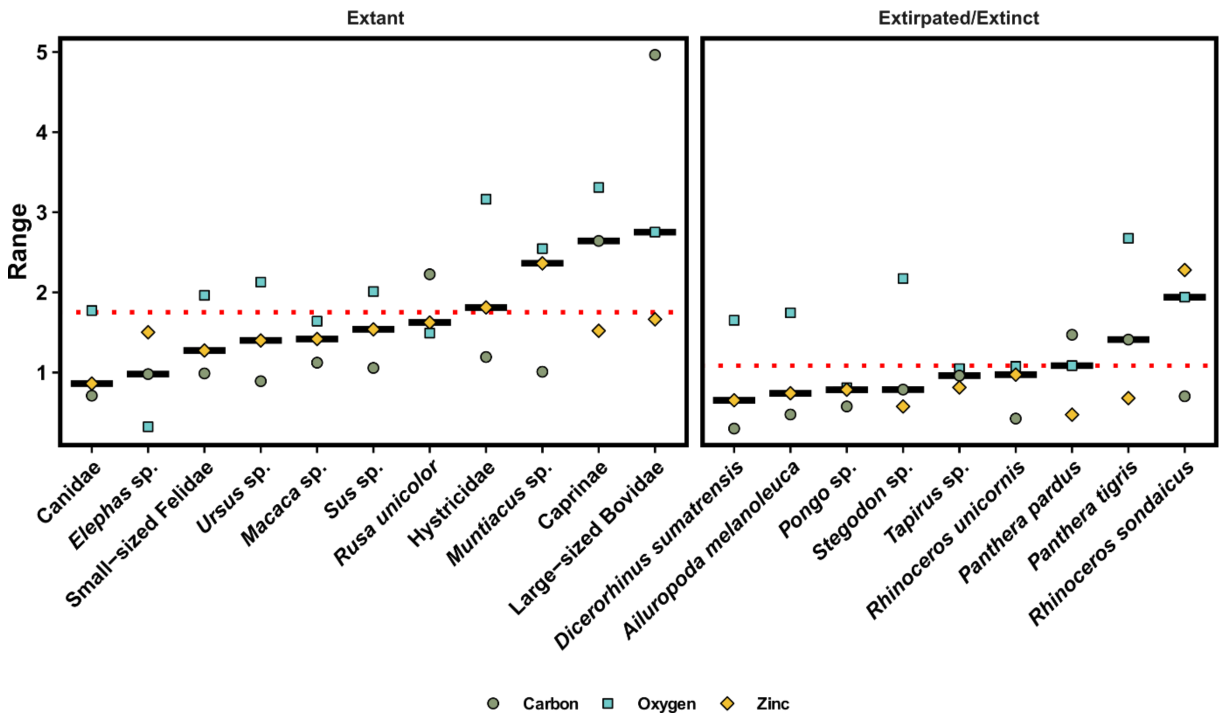

Figure S28. Average isotopic range of δ^{66}Zn (‰ JMC-Lyon), δ^{13}C (‰ VPDB), and δ^{18}O (‰ VPDB) of extant, locally-extirpated, and extinct taxa for the studied period. All isotopic values are standard-score transformed to allow direct comparison between the different isotope systems. Taxa found at only a single site were excluded, as their isotopic ranges only capture intra-site variability rather than temporal changes across sites. The range of every isotopic system was calculated for each taxon at each site (Tam Hay Marklot, Pà Hang Mountain, Duoi U'Oi, and Coc Muoi) and across sites (i.e., absolute maximum - absolute minimum), and then averaged into a single value for each isotopic system. The ranges calculated from a single individual (i.e., *n* = 1 for any given taxa at a given site) were excluded. The δ^{13}C values were converted to those of the food web's primary carbon sources for better comparability between taxa (see **Supplementary Material Text 1**). Both δ^{13}C and δ^{18}O values are taken from Bacon et al. *(*10*,* 46*)* and Bourgon et al. *(*27*,* 28*)*. The δ^{66}Zn values from Tam Hay Marklot are taken from Bourgon et al. *(*27*),* and those from Tam Pà Ling and Nam Lot from Bourgon et al. *(*28*)*. The δ^{66}Zn values of Coc Muoi are normalized to the average δ^{66}Zn value of all other Southeast Asian sites (i.e., Tam Hay Marklot, Pà Hang Mountain, and Duoi U'Oi). The median range of each taxa is represented by a bold horizontal line, and the red dotted line represents the average range for each panel (extant, locally-extirpated, and extinct).

A third sensitivity test addressed the issue of broad taxonomic assignments (e.g., "large Bovidae" or "Caprinae"), which may encompass multiple species and therefore introduce elevated isotopic variability. While such groupings are often unavoidable in fragmentary fossil assemblages, their exclusion would disproportionately reduce the ecological and temporal representativeness of the dataset and potentially introduce other biases. As such, we chose to retain these categories while acknowledging their limitations. To evaluate their influence, we ran a sensitivity analysis excluding the group with the highest isotopic variability: large-sized Bovidae (**fig. S29** and **tables S11** and **S12**). In a fourth and last sensitivity test, we assessed whether aggregating isotopic ranges across sites per proxy might blur or oversimplify meaningful variation (**fig. S30** and **tables S11** and **S12**).

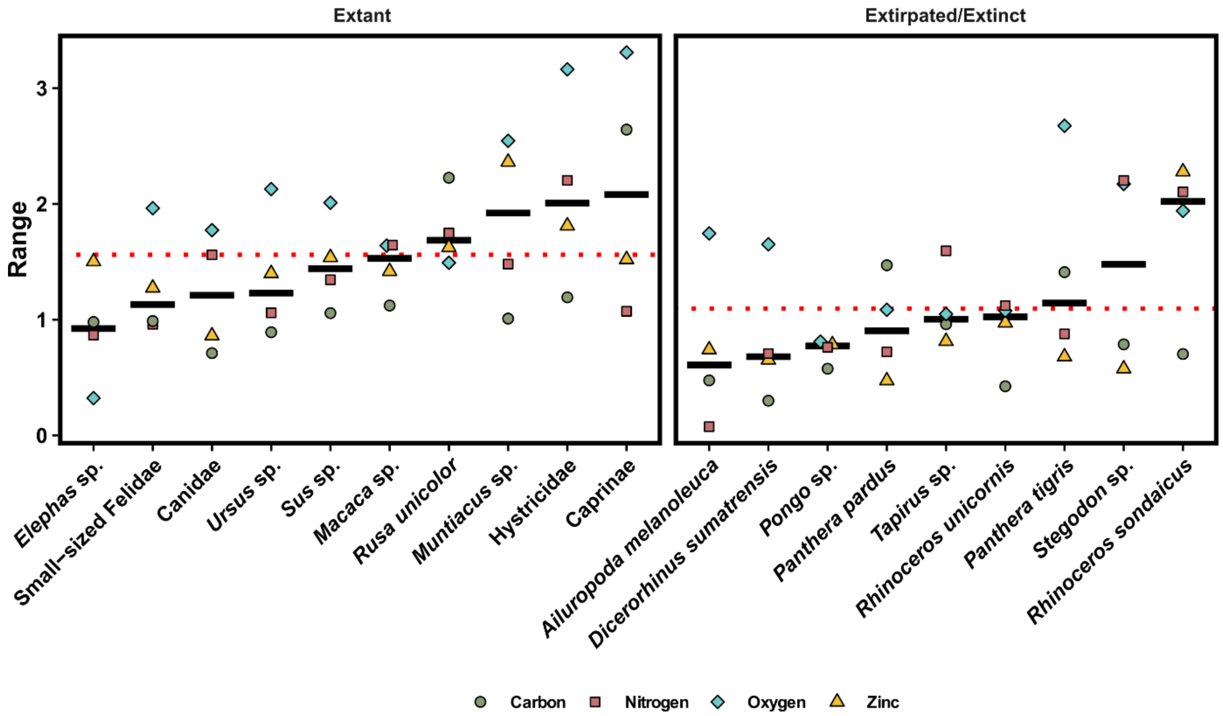

Figure S29. Average isotopic range of $\delta^{15}N_{enamel}$ (‰ AIR), $\delta^{66}Zn$ (‰ JMC-Lyon), $\delta^{13}C$ (‰ VPDB), and $\delta^{18}O$ (‰ VPDB) of extant, locally-extirpated, and extinct taxa for the studied period with the exclusion of the outlier group, Large-sized Bovidae. All isotopic values are standard-score transformed to allow direct comparison between the different isotope systems. Taxa found at only a single site were excluded, as their isotopic ranges only capture intra-site variability rather than temporal changes across sites. The range of every isotopic system was calculated for each taxon at each site (Tam Hay Marklot, Pà Hang Mountain, Duoi U'Oi, and Coc Muoi) and across sites (i.e., absolute maximum - absolute minimum), and then averaged into a single value for each isotopic system. The ranges calculated from a single individual (i.e., n = 1 for any given taxa at a given site) were excluded. The $\delta^{13}C$ values were converted to those of the food web's primary carbon sources for better comparability between taxa (see **Supplementary Material Text 1**). Both $\delta^{13}C$ and $\delta^{18}O$ values are taken from Bacon et al. *(*10*,* 46*)* and Bourgon et al. *(*27*,* 28*)*. The $\delta^{66}Zn$ values from Tam Hay Marklot are taken from Bourgon et al. *(*27*),* and those from Tam Pà Ling and Nam Lot from Bourgon et al. *(*28*)*. The $\delta^{15}N_{enamel}$ values from Tam Hay Marklot are taken from Leichliter et al. *(*19*)*. The $\delta^{66}Zn$ values of Coc Muoi are normalized to the average $\delta^{66}Zn$ value of all other Southeast Asian sites (i.e., Tam Hay Marklot, Pà Hang Mountain, and Duoi U'Oi). The median range of each taxa is represented by a bold horizontal line, and the red dotted line represents the average range for each panel (extant, locally-extirpated, and extinct).

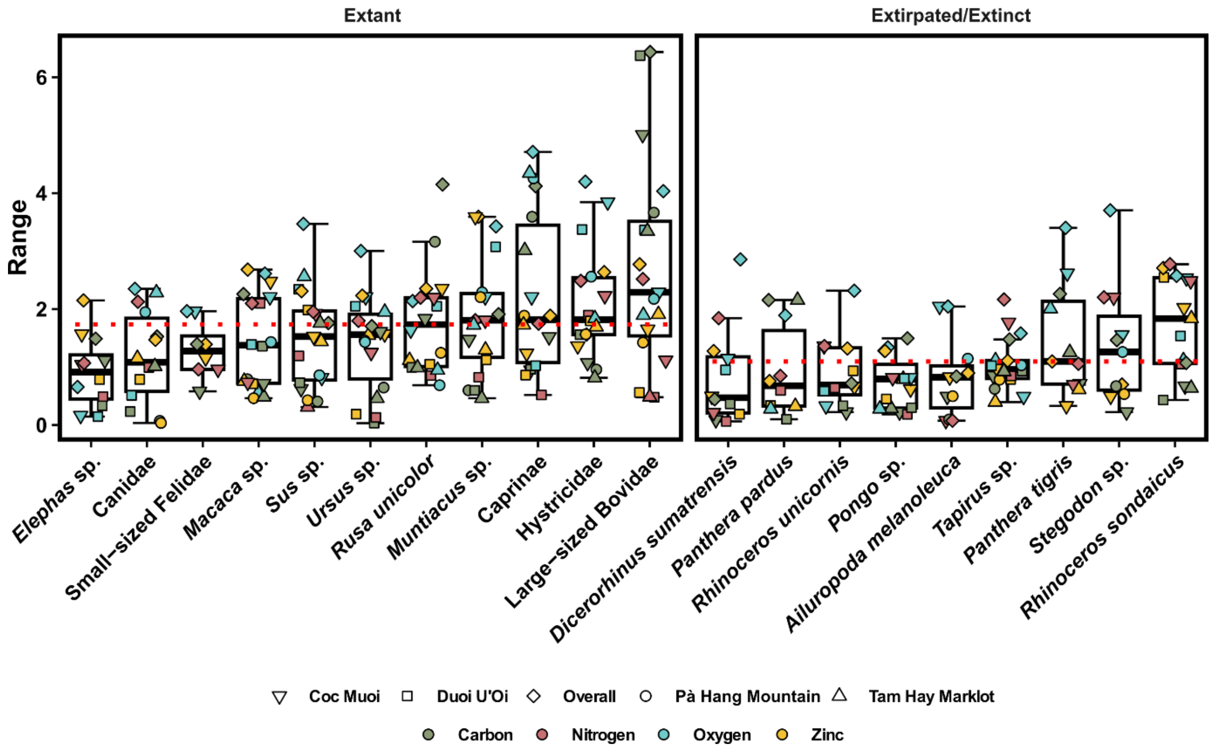

Figure S30. Average isotopic range of $\delta^{15}N_{enamel}$ (‰ AIR), $\delta^{66}Zn$ (‰ JMC-Lyon), $\delta^{13}C$ (‰ VPDB), and $\delta^{18}O$ (‰ VPDB) of extant, locally-extirpated, and extinct taxa for the studied period for all sites. All isotopic values are standard-score transformed to allow direct comparison between the different isotope systems. Taxa found at only a single site were excluded, as their isotopic ranges only capture intra-site variability rather than temporal changes across sites. The range of every isotopic system was calculated for each taxon at each site (Tam Hay Marklot, Pà Hang Mountain, Duoi U'Oi, and Coc Muoi) and across sites (i.e., absolute maximum - absolute minimum). The ranges calculated from a single individual (i.e., *n* = 1 for any given taxa at a given site) were excluded. The $\delta^{13}C$ values were converted to those of the food web's primary carbon sources for better comparability between taxa (see **Supplementary Material Text 1**). Both $\delta^{13}C$ and $\delta^{18}O$ values are taken from Bacon et al. *(*10*,* 46*)* and Bourgon et al. *(*27*,* 28*)*. The $\delta^{66}Zn$ values from Tam Hay Marklot are taken from Bourgon et al. *(*27*),* and those from Tam Pà Ling and Nam Lot from Bourgon et al. *(*28*)*. The $\delta^{15}N_{enamel}$ values from Tam Hay Marklot are taken from Leichliter et al. *(*19*)*. The $\delta^{66}Zn$ values of Coc Muoi are normalized to the average $\delta^{66}Zn$ value of all other Southeast Asian sites (i.e., Tam Hay Marklot, Pà Hang Mountain, and Duoi U'Oi). The boxes represent the 25th–75th percentiles, with the median represented by a bold horizontal line, and the red dotted line represents the average range for each panel (extant, locally-extirpated, and extinct).

Across all four sensitivity tests, our key predictors (particularly extinction status) remained stable in both direction and significance. These results collectively suggest that our main conclusions are not disproportionately driven by incomplete datasets, taxonomic lumping, or the choice to aggregate range values.

REFERENCES AND NOTES

1. N. Myers, R. A. Mittermeier, C. G. Mittermeier, G. A. B. da Fonseca, J. Kent, Biodiversity hotspots for conservation priorities. *Nature* **403**, 853–858 (2000).

2. R. C. Estoque, M. Ooba, V. Avitabile, Y. Hijioka, R. DasGupta, T. Togawa, Y. Murayama, The future of Southeast Asia's forests. *Nat. Commun.* **10**, 1829 (2019).

3. W. F. Laurance, J. Sayer, K. G. Cassman, Agricultural expansion and its impacts on tropical nature. *Trends. Ecol. Evol.* **29**, 107–116 (2014).

4. W. F. Laurance, G. R. Clements, S. Sloan, C. S. O'Connell, N. D. Mueller, M. Goosem, O. Venter, D. P. Edwards, B. Phalan, A. Balmford, R. Van Der Ree, I. B. Arrea, A global strategy for road building. *Nature* **513**, 229–232 (2014).

5. N. S. Sodhi, L. P. Koh, B. W. Brook, P. K. L. Ng, Southeast Asian biodiversity: An impending disaster. *Trends. Ecol. Evol.* **19**, 654–660 (2004).

6. L. P. Koh, N. S. Sodhi, Conserving Southeast Asia's imperiled biodiversity: Scientific, management, and policy challenges. *Biodivers. Conserv.* **19**, 913–917 (2010).

7. J. Louys, P. Roberts, Environmental drivers of megafauna and hominin extinction in Southeast Asia. *Nature* **586**, 402–406 (2020).

8. The IUCN Red List of Threatened Species, *IUCN Red List of Threatened Species* (2024). https://iucnredlist.org/fr.

9. D. Drucker, H. Bocherens, Carbon and nitrogen stable isotopes as tracers of change in diet breadth during Middle and Upper Palaeolithic in Europe. *Int. J. Osteoarchaeol.* **14**, 162–177 (2004).

10. A.-M. Bacon, N. Bourgon, F. Welker, E. Cappellini, D. Fiorillo, O. Tombret, N. T. M. Huong, N. A. Tuan, T. Sayavonkhamdy, V. Souksavatdy, P. Sichanthongtip, P.-O. Antoine, P. Duringer, J.-L. Ponche, K. Westaway, R. Joannes-Boyau, Q. Boesch, E. Suzzoni, S. Frangeul, E. Patole-Edoumba, A. Zachwieja, L. Shackelford, F. Demeter, J.-J. Hublin, É. Dufour, A

multi-proxy approach to exploring *Homo sapiens*' arrival, environments and adaptations in Southeast Asia. *Sci. Rep.* **11**, 21080 (2021).

11. P.-A. Christin, C. P. Osborne, The evolutionary ecology of C_4 plants. *New Phytol.* **204**, 765–781 (2014).

12. G. D. Farquhar, J. R. Ehleringer, K. T. Hubick, Carbon isotope discrimination and photosynthesis. *Annu. Rev. Plant Physiol. Plant Mol. Biol.* **40**, 503–537 (1989).

13. N. J. van der Merwe, E. Medina, The canopy effect, carbon isotope ratios and foodwebs in Amazonia. *J. Archaeol. Sci.* **18**, 249–259 (1991).

14. S. Pederzani, K. Britton, Oxygen isotopes in bioarchaeology: Principles and applications, challenges and opportunities. *Earth Sci. Rev.* **188**, 77–107 (2019).

15. M. J. Schoeninger, M. J. DeNiro, Nitrogen and carbon isotopic composition of bone collagen from marine and terrestrial animals. *Geochim. Cosmochim. Acta* **48**, 625–639 (1984).

16. R. D. Evans, Physiological mechanisms influencing plant nitrogen isotope composition. *Trends Plant Sci.* **6**, 121–126 (2001).

17. M. M. Casey, D. M. Post, The problem of isotopic baseline: Reconstructing the diet and trophic position of fossil animals. *Earth Sci. Rev.* **106**, 131–148 (2011).

18. J. N. Leichliter, T. Lüdecke, A. D. Foreman, N. N. Duprey, D. E. Winkler, E. R. Kast, H. Vonhof, D. M. Sigman, G. H. Haug, M. Clauss, T. Tütken, A. Martínez-García, Nitrogen isotopes in tooth enamel record diet and trophic level enrichment: Results from a controlled feeding experiment. *Chem. Geol.* **563**, 120047 (2021).

19. J. N. Leichliter, T. Lüdecke, A. D. Foreman, N. Bourgon, N. N. Duprey, H. Vonhof, V. Souksavatdy, A.-M. Bacon, D. M. Sigman, T. Tütken, A. Martínez-García, Tooth enamel nitrogen isotope composition records trophic position: A tool for reconstructing food webs. *Commun. Biol.* **6**, 373 (2023).

20. T. Lüdecke, J. N. Leichliter, V. Aldeias, M. K. Bamford, D. Biro, D. R. Braun, C. Capelli, J. D. Cybulski, N. N. Duprey, M. J. Ferreira da Silva, A. D. Foreman, J. M. Habermann, G. H. Haug, F. I. Martínez, J. Mathe, A. Mulch, D. M. Sigman, H. Vonhof, R. Bobe, S. Carvalho, A. Martínez-García, Carbon, nitrogen, and oxygen stable isotopes in modern tooth enamel: A case study from Gorongosa National Park, central Mozambique. *Front. Ecol. Evol.* **10**, doi.org/10.3389/fevo.2022.958032 (2022).

21. S. Caut, E. Angulo, F. Courchamp, Variation in discrimination factors (Δ^{15}N and Δ^{13}C): The effect of diet isotopic values and applications for diet reconstruction. *J. Appl. Ecol.* **46**, 443–453 (2009).

22. B. E. Lowry, R. M. Wittig, J. Pittermann, V. M. Oelze, Stratigraphy of stable isotope ratios and leaf structure within an African rainforest canopy with implications for primate isotope ecology. *Sci. Rep.* **11**, 14222 (2021).

23. Y. Zhang, K. E. Westaway, S. Haberle, J. K. Lubeek, M. Bailey, R. Ciochon, M. W. Morley, P. Roberts, J. Zhao, M. Duval, A. Dosseto, Y. Pan, S. Rule, W. Liao, G. A. Gully, M. Lucas, J. Mo, L. Yang, Y. Cai, W. Wang, R. Joannes-Boyau, The demise of the giant ape *Gigantopithecus blacki*. *Nature* **625**, 535–539 (2024).

24. K. Jaouen, M. Beasley, M. Schoeninger, J.-J. Hublin, M. P. Richards, Zinc isotope ratios of bones and teeth as new dietary indicators: Results from a modern food web (Koobi Fora, Kenya). *Sci. Rep.* **6**, srep26281 (2016).

25. K. Jaouen, P. Szpak, M. P. Richards, Zinc isotope ratios as indicators of diet and trophic level in arctic marine mammals. *PLOS ONE* **11**, e0152299 (2016).

26. K. Jaouen, V. Villalba-Mouco, G. M. Smith, M. Trost, J. Leichliter, T. Lüdecke, P. Méjean, S. Mandrou, J. Chmeleff, D. Guiserix, N. Bourgon, F. Blasco, J. Mendes Cardoso, C. Duquenoy, Z. Moubtahij, D. C. Salazar Garcia, M. Richards, T. Tütken, J.-J. Hublin, P. Utrilla, L. Montes, A Neandertal dietary conundrum: Insights provided by tooth enamel Zn isotopes from Gabasa, Spain. *Proc. Natl. Acad. Sci. U.S.A.* **119**, e2109315119 (2022).

27. N. Bourgon, K. Jaouen, A.-M. Bacon, K. P. Jochum, E. Dufour, P. Duringer, J.-L. Ponche, R. Joannes-Boyau, Q. Boesch, P.-O. Antoine, M. Hullot, U. Weis, E. Schulz-Kornas, M. Trost, D. Fiorillo, F. Demeter, E. Patole-Edoumba, L. L. Shackelford, T. E. Dunn, A. Zachwieja, S. Duangthongchit, T. Sayavonkhamdy, P. Sichanthongtip, D. Sihanam, V. Souksavatdy, J.-J. Hublin, T. Tütken, Zinc isotopes in Late Pleistocene fossil teeth from a Southeast Asian cave setting preserve paleodietary information. *Proc. Natl. Acad. Sci. U.S.A.* **117**, 4675–4681 (2020).

28. N. Bourgon, K. Jaouen, A.-M. Bacon, E. Dufour, J. McCormack, N.-H. Tran, M. Trost, D. Fiorillo, T. E. Dunn, C. Zanolli, A. Zachwieja, P. Duringer, J.-L. Ponche, Q. Boesch, P.-O. Antoine, K. E. Westaway, R. Joannes-Boyau, E. Suzzoni, S. Frangeul, F. Crozier, F. Aubaile, E. Patole-Edoumba, T. Luangkhoth, V. Souksavatdy, S. Boualaphane, T. Sayavonkhamdy, P. Sichanthongtip, D. Sihanam, F. Demeter, L. L. Shackelford, J.-J. Hublin, T. Tütken, Trophic ecology of a Late Pleistocene early modern human from tropical Southeast Asia inferred from zinc isotopes. *J. Hum. Evol.* **161**, 103075 (2021).

29. J. McCormack, P. Szpak, N. Bourgon, M. Richards, C. Hyland, P. Méjean, J.-J. Hublin, K. Jaouen, Zinc isotopes from archaeological bones provide reliable trophic level information for marine mammals. *Commun. Biol.* **4**, 26281 (2021).

30. J. McCormack, M. L. Griffiths, S. L. Kim, K. Shimada, M. Karnes, H. Maisch, S. Pederzani, N. Bourgon, K. Jaouen, M. A. Becker, N. Jöns, G. Sisma-Ventura, N. Straube, J. Pollerspöck, J.-J. Hublin, R. A. Eagle, T. Tütken, Trophic position of *Otodus megalodon* and great white sharks through time revealed by zinc isotopes. *Nat. Commun.* **13**, 2980 (2022).

31. Z. Moubtahij, J. McCormack, N. Bourgon, M. Trost, V. Sinet-Mathiot, B. T. Fuller, G. M. Smith, H. Temming, S. Steinbrenner, J.-J. Hublin, A. Bouzouggar, E. Turner, K. Jaouen, Isotopic evidence of high reliance on plant food among Later Stone Age hunter-gatherers at Taforalt, Morocco. *Nat. Ecol. Evol.* **8**, 1035–1045 (2024).

32. Y. Wang, T. E. Cerling, A model of fossil tooth and bone diagenesis: Implications for paleodiet reconstruction from stable isotopes. *Palaeogeogr. Palaeoclimatol. Palaeoecol.* **107**, 281–289 (1994).

33. M. J. Kohn, M. J. Schoeninger, W. W. Barker, Altered states: Effects of diagenesis on fossil tooth chemistry. *Geochim. Cosmochim. Acta* **63**, 2737–2747 (1999).

34. J. d. D. Teruel, A. Alcolea, A. Hernández, A. J. O. Ruiz, Comparison of chemical composition of enamel and dentine in human, bovine, porcine and ovine teeth. *Arch. Oral Biol.* **60**, 768–775 (2015).

35. A. Martínez-García, J. Jung, X. E. Ai, D. M. Sigman, A. Auderset, N. N. Duprey, A. Foreman, F. Fripiat, J. Leichliter, T. Lüdecke, S. Moretti, T. Wald, Laboratory assessment of the impact of chemical oxidation, mineral dissolution, and heating on the nitrogen isotopic composition of fossil-bound organic matter. *Geochem. Geophys. Geosyst.* **23**, e2022GC010396 (2022).

36. S. Opfergelt, J. T. Cornélis, D. Houben, C. Givron, K. W. Burton, N. Mattielli, The influence of weathering and soil organic matter on Zn isotopes in soils. *Chem. Geol.* **466**, 140–148 (2017).

37. M. Wiggenhauser, M. Bigalke, M. Imseng, A. Keller, C. Archer, W. Wilcke, E. Frossard, Zinc isotope fractionation during grain filling of wheat and a comparison of zinc and cadmium isotope ratios in identical soil–plant systems. *New Phytol.* **219**, 195–205 (2018).

38. J. McCormack, K. Jaouen, N. Bourgon, G. Sisma-Ventura, T. J. G. Tacail, W. Müller, T. Tütken, Zinc isotope composition of enameloid, bone and muscle of gilt-head seabreams (*Sparus aurata*) raised in pisciculture and their relation to diet. *Mar. Biol.* **171**, 65 (2024).

39. N. Bourgon, T. Tacail, K. Jaouen, J. N. Leichliter, J. McCormack, D. E. Winkler, M. Clauss, T. Tütken, Dietary and homeostatic controls of Zn isotopes in rats: A controlled feeding experiment and modeling approach. *Metallomics* **16**, mfae026 (2024).

40. V. Balter, A. Lamboux, A. Zazzo, P. Télouk, Y. Leverrier, J. Marvel, A. P. Moloney, F. J. Monahan, O. Schmidt, F. Albarède, Contrasting Cu, Fe, and Zn isotopic patterns in organs and body fluids of mice and sheep, with emphasis on cellular fractionation. *Metallomics* **5**, 1470–1482 (2013).

41. F. Moynier, T. Fujii, A. S. Shaw, M. L. Borgne, Heterogeneous distribution of natural zinc isotopes in mice. *Metallomics* **5**, 693–699 (2013).

42. T. Fujii, F. Moynier, J. Blichert-Toft, F. Albarède, Density functional theory estimation of isotope fractionation of Fe, Ni, Cu, and Zn among species relevant to geochemical and biological environments. *Geochim. Cosmochim. Acta* **140**, 553–576 (2014).

43. B. Mahan, F. Moynier, A. L. Jørgensen, M. Habekost, J. Siebert, Examining the homeostatic distribution of metals and Zn isotopes in Göttingen minipigs. *Metallomics* **10**, 1264–1281 (2018).

44. A.-M. Bacon, P.-O. Antoine, N. T. M. Huong, K. Westaway, N. A. Tuan, P. Duringer, J. Zhao, J.-L. Ponche, S. C. Dung, T. H. Nghia, T. T. Minh, P. T. Son, M. Boyon, N. T. K. Thuy, A. Blin, F. Demeter, A rhinocerotid-dominated megafauna at the MIS6–5 transition: The late Middle Pleistocene Coc Muoi assemblage, Lang Son province, Vietnam. *Quat. Sci. Rev.* **186**, 123–141 (2018).

45. A.-M. Bacon, K. Westaway, P.-O. Antoine, P. Duringer, A. Blin, F. Demeter, J.-L. Ponche, J.-X. Zhao, L. M. Barnes, T. Sayavonkhamdy, N. T. K. Thuy, V. T. Long, E. Patole-Edoumba, L. Shackelford, Late Pleistocene mammalian assemblages of Southeast Asia: New dating, mortality profiles and evolution of the predator–prey relationships in an environmental context. *Palaeogeogr. Palaeoclimatol. Palaeoecol.* **422**, 101–127 (2015).

46. A.-M. Bacon, N. Bourgon, E. Dufour, C. Zanolli, P. Duringer, J.-L. Ponche, P.-O. Antoine, L. Shackelford, N. T. M. Huong, T. Sayavonkhamdy, E. Patole-Edoumba, F. Demeter, Nam Lot (MIS 5) and Duoi U'Oi (MIS 4) Southeast Asian sites revisited: Zooarchaeological and isotopic evidences. *Palaeogeogr. Palaeoclimatol. Palaeoecol.* **512**, 132–144 (2018).

47. A.-M. Bacon, F. Demeter, P. Duringer, C. Helm, M. Bano, V. T. Long, N. T. Kim Thuy, P.-O. Antoine, B. Thi Mai, N. T. M. Huong, Y. Dodo, F. Chabaux, S. Rihs, The Late Pleistocene Duoi U'Oi cave in northern Vietnam: Palaeontology, sedimentology, taphonomy and palaeoenvironments. *Quat. Sci. Rev.* **27**, 1627–1654 (2008).

48. S. E. Freidline, K. E. Westaway, R. Joannes-Boyau, P. Duringer, J.-L. Ponche, M. W. Morley, V. C. Hernandez, M. S. McAllister-Hayward, H. McColl, C. Zanolli, P. Gunz, I. Bergmann, P. Sichanthongtip, D. Sihanam, S. Boualaphane, T. Luangkhoth, V. Souksavatdy, A. Dosseto, Q. Boesch, E. Patole-Edoumba, F. Aubaile, F. Crozier, E. Suzzoni, S. Frangeul, N. Bourgon, A. Zachwieja, T. E. Dunn, A.-M. Bacon, J.-J. Hublin, L. Shackelford, F. Demeter, Early presence of *Homo sapiens* in Southeast Asia by 86–68 kyr at Tam Pà Ling, Northern Laos. *Nat. Commun.* **14**, 3193 (2023).

49. S. A. Blumenthal, J. M. Rothman, K. L. Chritz, T. E. Cerling, Stable isotopic variation in tropical forest plants for applications in primatology. *Am. J. Primatol.* **78**, 1041–1054 (2016).

50. S. A. Blumenthal, K. L. Chritz, J. M. Rothman, T. E. Cerling, Detecting intraannual dietary variability in wild mountain gorillas by stable isotope analysis of feces. *Proc. Natl. Acad. Sci. U.S.A.* **109**, 21277–21282 (2012).

51. S. A. Ballari, M. N. Barrios-García, A review of wild boar *Sus scrofadiet* and factors affecting food selection in native and introduced ranges. *Mamm. Rev.* **44**, 124–134 (2014).

52. M. Wiggenhauser, R. E. T. Moore, P. Wang, G. P. Bienert, K. H. Laursen, S. Blotevogel, Stable isotope fractionation of metals and metalloids in plants: A review. *Front. Plant Sci.* **13**, 840941 (2022).

53. K. J. Kolb, R. D. Evans, Implications of leaf nitrogen recycling on the nitrogen isotope composition of deciduous plant tissues. *New Phytol.* **156**, 57–64 (2002).

54. S. Pederzani, K. Britton, M. Trost, H. Fewlass, N. Bourgon, J. McCormack, K. Jaouen, H. Dietl, H.-J. Döhle, A. Kirchner, T. Lauer, M. Le Corre, S. P. McPherron, H. Meller, D. Mylopotamitaki, J. Orschiedt, H. Rougier, K. Ruebens, T. Schüler, V. Sinet-Mathiot, G. M. Smith, S. Talamo, T. Tütken, F. Welker, E. I. Zavala, M. Weiss, J.-J. Hublin, Stable isotopes show *Homo sapiens* dispersed into cold steppes ~45,000 years ago at Ilsenhöhle in Ranis, Germany. *Nat. Ecol. Evol.* **8**, 578–588 (2024).

55. K. Jaouen, M. Trost, N. Bourgon, R. Colleter, A. Le Cabec, T. Tütken, R. E. Oliveira, M. L. Pons, P. Méjean, S. Steinbrenner, J. Chmeleff, A. Strauss, Zinc isotope variations in

archeological human teeth (Lapa do Santo, Brazil) reveal dietary transitions in childhood and no contamination from gloves. *PLOS ONE* **15**, e0232379 (2020).

56. H. Thanh Tran, B. Van Dang, C. Kim Ngo, Q. Dinh Hoang, Q. Minh Nguyen, Structural controls on the occurrence and morphology of karstified assemblages in northeastern Vietnam: A regional perspective. *Environ. Earth Sci.* **70**, 511–520 (2013).

57. S. Pichat, C. Douchet, F. Albarède, Zinc isotope variations in deep-sea carbonates from the eastern equatorial Pacific over the last 175 ka. *Earth Planet. Sci. Lett.* **210**, 167–178 (2003).

58. L. E. Lisiecki, M. E. Raymo, A Pliocene-Pleistocene stack of 57 globally distributed benthic δ^{18}O records. *Paleoceanography* **20**, doi.org/10.1029/2004PA001071 (2005).

59. W. Dansgaard, H. B. Clausen, N. Gundestrup, C. U. Hammer, S. F. Johnsen, P. M. Kristinsdottir, N. Reeh, A new Greenland deep ice core. *Science* **218**, 1273–1277 (1982).

60. N. Amano, F. Rivals, A.-M. Moigne, T. Ingicco, F. Sémah, T. Simanjuntak, Paleoenvironment in East Java during the last 25,000 years as inferred from bovid and cervid dental wear analyses. *J. Archaeol. Sci. Rep.* **10**, 155–165 (2016).

61. R. Hamilton, N. Amano, C. J. A. Bradshaw, F. Saltré, R. Patalano, D. Penny, J. Stevenson, J. Wolfhagen, P. Roberts, Forest mosaics, not savanna corridors, dominated in Southeast Asia during the Last Glacial Maximum. *Proc. Natl. Acad. Sci. U.S.A.* **121**, e2311280120 (2024).

62. F. S. Ahrestani, M. Sankaran, Eds., *The Ecology of Large Herbivores of South and Southeast Asia* (Springer Netherlands, 2016), vol. 225 of *Ecological Studies*.

63. N. L. Boivin, M. A. Zeder, D. Q. Fuller, A. Crowther, G. Larson, J. M. Erlandson, T. Denham, M. D. Petraglia, Ecological consequences of human niche construction: Examining long-term anthropogenic shaping of global species distributions. *Proc. Natl. Acad. Sci. U.S.A.* **113**, 6388–6396 (2016).

64. K. E. Westaway, J. Louys, R. D. Awe, M. J. Morwood, G. J. Price, J.-x. Zhao, M. Aubert, R. Joannes-Boyau, T. M. Smith, M. M. Skinner, T. Compton, R. M. Bailey, G. D. van den Bergh, J. de Vos, A. W. G. Pike, C. Stringer, E. W. Saptomo, Y. Rizal, J. Zaim, W. D. Santoso,

A. Trihascaryo, L. Kinsley, B. Sulistyanto, An early modern human presence in Sumatra 73,000–63,000 years ago. *Nature* **548**, 322–325 (2017).

65. Y. Wang, H. Cheng, R. L. Edwards, X. Kong, X. Shao, S. Chen, J. Wu, X. Jiang, X. Wang, Z. An, Millennial- and orbital-scale changes in the East Asian monsoon over the past 224,000 years. *Nature* **451**, 1090–1093 (2008).

66. C. P. Groves, D. M. Leslie, Rhinoceros sondaicus (Perissodactyla: Rhinocerotidae). *Mamm. Species.* **43**, 190–208 (2011).

67. T. J. Foose, M. K. bin M. Khan, N. J. van Strien, *Asian Rhinos: Status Survey and Conservation Action Plan* (IUCN, 1997).

68. J. Louys, D. Curnoe, H. Tong, Characteristics of Pleistocene megafauna extinctions in Southeast Asia. *Palaeogeogr. Palaeoclimatol. Palaeoecol.* **243**, 152–173 (2007).

69. S. N. Spehar, D. Sheil, T. Harrison, J. Louys, M. Ancrenaz, A. J. Marshall, S. A. Wich, M. W. Bruford, E. Meijaard, Orangutans venture out of the rainforest and into the Anthropocene. *Sci. Adv.* **4**, e1701422 (2018).

70. E. A. Fox, C. P. van Schaik, A. Sitompul, D. N. Wright, Intra-and interpopulational differences in orangutan (*Pongo pygmaeus*) activity and diet: Implications for the invention of tool use. *Am. J. Phys. Anthropol.* **125**, 162–174 (2004).

71. S. A. Wich, S. S. Utami-Atmoko, T. Mitra Setia, S. Djoyosudharmo, M. L. Geurts, Dietary and energetic responses of *Pongo abelii* to fruit availability fluctuations. *Int. J. Primatol.* **27**, 1535–1550 (2006).

72. M. E. Hardus, A. R. Lameira, A. Zulfa, S. S. U. Atmoko, H. de Vries, S. A. Wich, Behavioral, ecological, and evolutionary aspects of meat-eating by Sumatran orangutans (*Pongo abelii*). *Int. J. Primatol.* **33**, 287–304 (2012).

73. J. Louys, Y. Zaim, Y. Rizal, Aswan, M. Puspaningrum, A. Trihascaryo, G. J. Price, A. Petherick, E. Scholtz, L. R. G. DeSantis, Sumatran orangutan diets in the Late Pleistocene as inferred from dental microwear texture analysis. *Quat. Int.* **603**, 74–81 (2021).

74. D. J. Weiss, T. F. D. Mason, F. J. Zhao, G. J. D. Kirk, B. J. Coles, M. S. A. Horstwood, Isotopic discrimination of zinc in higher plants. *New Phytol.* **165**, 703–710 (2005).

75. J. Viers, P. Oliva, A. Nonell, A. Gélabert, J. E. Sonke, R. Freydier, R. Gainville, B. Dupré, Evidence of Zn isotopic fractionation in a soil–plant system of a pristine tropical watershed (Nsimi, Cameroon). *Chem. Geol.* **239**, 124–137 (2007).

76. F. Moynier, S. Pichat, M.-L. Pons, D. Fike, V. Balter, F. Albarède, Isotopic fractionation and transport mechanisms of Zn in plants. *Chem. Geol.* **267**, 125–130 (2009).

77. N. Amano, Y. V. Wang, N. Boivin, P. Roberts, 'Emptying forests?' Conservation implications of past human–primate interactions. *Trends Ecol. Evol.* **36**, 345–359 (2021).

78. R. A. Delgado Jr., C. P. Van Schaik, The behavioral ecology and conservation of the orangutan (*Pongo pygmaeus*): A tale of two islands. *Evol. Anthropol.* **9**, 201–218 (2000).

79. A. E. Russon, S. A. Wich, M. Ancrenaz, T. Kanamori, C. D. Knott, N. Kuze, H. C. Morrogh-Bernard, P. Pratje, H. Ramlee, P. Rodman, A. Sawang, K. Sidiyasa, I. Singleton, C. P. van Schaik, "Geographic variation in orangutan diets" in *Orangutans: Geographic Variation in Behavioral Ecology and Conservation*, S. A. Wich, U.-A. S. Suci, T. M. Setia, C. P. van Schaik, Eds. (Oxford Univ. Press, 2008), pp. 135–156.

80. M. Ancrenaz, F. Oram, N. Nardiyono, M. Silmi, M. E. M. Jopony, M. Voigt, D. J. I. Seaman, J. Sherman, I. Lackman, C. Traeholt, S. A. Wich, T. Santika, M. J. Struebig, E. Meijaard, Importance of small forest fragments in agricultural landscapes for maintaining orangutan metapopulations. *Front. For. Glob. Change* **4**, 560944 (2021).

81. J. Ma, Y. Wang, C. Jin, Y. Yan, Y. Qu, Y. Hu, Isotopic evidence of foraging ecology of Asian elephant (*Elephas maximus*) in South China during the Late Pleistocene. *Quat. Int.* **443**, 160–167 (2017).

82. J. Ma, Y. Wang, C. Jin, Y. Hu, H. Bocherens, Ecological flexibility and differential survival of Pleistocene *Stegodon orientalis* and *Elephas maximus* in mainland southeast Asia revealed by stable isotope (C, O) analysis. *Quat. Sci. Rev.* **212**, 33–44 (2019).

83. T. W. Schoener, Theory of feeding strategies. *Annu. Rev. Ecol. Evol. Syst.* **2**, 369–404 (1971).

84. E. R. Pianka, Niche overlap and diffuse competition. *Proc. Natl. Acad. Sci. U.S.A.* **71**, 2141–2145 (1974).

85. J. H. Brown, On the relationship between abundance and distribution of species. *Am. Nat.* **124**, 255–279 (1984).

86. J. J. Wiens, C. H. Graham, Niche conservatism: Integrating evolution, ecology, and conservation biology. *Annu. Rev. Ecol. Evol. Syst.* **36**, 519–539 (2005).

87. K. Tamma, U. Ramakrishnan, Higher speciation and lower extinction rates influence mammal diversity gradients in Asia. *BMC Evol. Biol.* **15**, 11 (2015).

88. N. S. Sodhi, M. R. C. Posa, T. M. Lee, D. Bickford, L. P. Koh, B. W. Brook, The state and conservation of Southeast Asian biodiversity. *Biodivers. Conserv.* **19**, 317–328 (2010).

89. M. Castelletta, N. S. Sodhi, R. Subaraj, Heavy extinctions of forest avifauna in Singapore: Lessons for biodiversity conservation in Southeast Asia. *Conserv. Biol.* **14**, 1870–1880 (2000).

90. B. W. Brook, N. S. Sodhi, P. K. L. Ng, Catastrophic extinctions follow deforestation in Singapore. *Nature* **424**, 420–423 (2003).

91. R. J. Lee, A. J. Gorog, A. Dwiyahreni, S. Siwu, J. Riley, H. Alexander, G. D. Paoli, W. Ramono, Wildlife trade and implications for law enforcement in Indonesia: A case study from North Sulawesi. *Biol. Conserv.* **123**, 477–488 (2005).

92. P. Duringer, A.-M. Bacon, T. Sayavongkhamdy, T. K. T. Nguyen, Karst development, breccias history, and mammalian assemblages in Southeast Asia: A brief review. *C. R. Palevol* **11**, 133–157 (2012).

93. F. Moynier, F. Albarède, G. F. Herzog, Isotopic composition of zinc, copper, and iron in lunar samples. *Geochim. Cosmochim. Acta* **70**, 6103–6117 (2006).

94. J.-P. Toutain, J. Sonke, M. Munoz, A. Nonell, M. Polvé, J. Viers, R. Freydier, F. Sortino, J.-L. Joron, S. Sumarti, Evidence for Zn isotopic fractionation at Merapi volcano. *Chem. Geol.* **253**, 74–82 (2008).

95. A. J. McCoy-West, J. G. Fitton, M.-L. Pons, E. C. Inglis, H. M. Williams, The Fe and Zn isotope composition of deep mantle source regions: Insights from Baffin Island picrites. *Geochim. Cosmochim. Acta* **238**, 542–562 (2018).

96. H. Ren, D. M. Sigman, A. N. Meckler, B. Plessen, R. S. Robinson, Y. Rosenthal, G. H. Haug, Foraminiferal isotope evidence of reduced nitrogen fixation in the ice age Atlantic Ocean. *Science* **323**, 244–248 (2009).

97. A. N. Knapp, D. M. Sigman, F. Lipschultz, N isotopic composition of dissolved organic nitrogen and nitrate at the Bermuda Atlantic Time-series Study site. *Glob. Biogeochem. Cycles* **19**, doi.org/10.1029/2004GB002320 (2005).

98. D. M. Sigman, K. L. Casciotti, M. Andreani, C. Barford, M. Galanter, J. K. Böhlke, A bacterial method for the nitrogen isotopic analysis of nitrate in seawater and freshwater. *Anal. Chem.* **73**, 4145–4153 (2001).

99. M. A. Weigand, J. Foriel, B. Barnett, S. Oleynik, D. M. Sigman, Updates to instrumentation and protocols for isotopic analysis of nitrate by the denitrifier method. *Rapid Commun. Mass Spectrom.* **30**, 1365–1383 (2016).

100. R Core Team, R: A language and environment for statistical computing. *R Foundation for Statistical Computing, Vienna, Austria*, v 4.0.2 (2023).

101. M. Lysy, A. D. Stasko, H. K. Swanson, Niche region and niche overlap metrics for multidimensional ecological niches. v 1.1.12 (2023).

102. H. Wickham, M. Averick, J. Bryan, W. Chang, L. D. McGowan, R. François, G. Grolemund, A. Hayes, L. Henry, J. Hester, M. Kuhn, T. L. Pedersen, E. Miller, S. M. Bache, K. Müller, J. Ooms, D. Robinson, D. P. Seidel, V. Spinu, K. Takahashi, D. Vaughan, C. Wilke, K. Woo, H. Yutani, Welcome to the Tidyverse. *J. Open Source Softw.* **4**, 1686 (2019).

103. C. O. Wilke, Package “cowplot”: Streamlined plot theme and plot annotations for ‘ggplot2.’ 1.1.3 (2024).

104. A. Kassambara, rstatix: Pipe-friendly framework for basic statistical tests. 0.7.2 (2023).

105. D. Bates, M. Mächler, B. Bolker, S. Walker, Fitting linear mixed-effects models using lme4. *J. Stat. Softw.* **67**, 1–48 (2015).

106. A. Kuznetsova, P. B. Brockhoff, C. R. H. Bojesen, lmerTest: Tests in linear mixed effects models. *J. Stat. Softw.* **83**, 1–26 (2017).

107. M. C. Dean, J. Garrevoet, S. J. M. Van Malderen, F. Santos, M. Mirazón Lahr, R. Foley, A. Le Cabec, The distribution and biogenic origins of zinc in the mineralised tooth tissues of modern and fossil hominoids: Implications for life history, diet and taphonomy. *Biology* **12**, 1455 (2023).

108. S. Hillson, “Teeth” in *Cambridge Manuals in Archaeology* (Cambridge Univ. Press, ed. 2, 2005).

109. B. H. Smith, T. L. Crummett, K. L. Brandt, Ages of eruption of primate teeth: A compendium for aging individuals and comparing life histories. *Am. J. Phys. Anthropol.* **37**, 177–231 (1994).

110. S. A. Marks, A. W. Erickson, Age determination in the black bear. *J. Wildl. Manag.* **30**, 389–410 (1966).

111. M. Fortelius, Ungulate cheek teeth: Developmental, functional, and evolutionary interrelation. *Acta Zool. Fenn.* **180**, AC957J (1985).

112. M. W. Colbert, “Patterns of evolution and variation in the Tapiroidea (Mammalia: Perissodactyla),” thesis, The University of Texas at Austin, Austin, TX (1999).

113. R. M. Laws, Age criteria for the African elephant. *Afr. J. Ecol.* **4**, 1–37 (1966).

114. B. H. Passey, T. F. Robinson, L. K. Ayliffe, T. E. Cerling, M. Sponheimer, M. D. Dearing, B. L. Roeder, J. R. Ehleringer, Carbon isotope fractionation between diet, breath CO_2, and bioapatite in different mammals. *J. Archaeol. Sci.* **32**, 1459–1470 (2005).

115. J. V. Tejada-Lara, B. J. MacFadden, L. Bermudez, G. Rojas, R. Salas-Gismondi, J. J. Flynn, Body mass predicts isotope enrichment in herbivorous mammals. *Proc. R. Soc. B* **285**, 20181020 (2018).

116. H. Han, W. Wei, Y. Nie, W. Zhou, Y. Hu, Q. Wu, F. Wei, Distinctive diet-tissue isotopic discrimination factors derived from the exclusive bamboo-eating giant panda. *Integr. Zoo.* **11**, 447–456 (2016).

117. K. Fox-Dobbs, J. K. Bump, R. O. Peterson, D. L. Fox, P. L. Koch, Carnivore-specific stable isotope variables and variation in the foraging ecology of modern and ancient wolf populations: Case studies from Isle Royale, Minnesota, and La Brea. *Can. J. Zool*. **85**, 458–471 (2007).

118. M. N. Barrios-Garcia, S. A. Ballari, Impact of wild boar (*Sus scrofa*) in its introduced and native range: A review. *Biol. Invasions* **14**, 2283–2300 (2012).

119. R. J. Timmins, J. W. Duckworth, IUCN Red List of Threatened Species: *Muntiacus rooseveltorum. IUCN Red List of Threatened Species* (2015).

120. R. J. Timmins, J. W. Duckworth, IUCN Red List of Threatened Species: *Muntiacus truongsonensis. IUCN Red List of Threatened Species* (2014).

121. R. Timmins, J. W. Duckworth, W. Robichaud, B. Long, T. Gray, A. Tilker, IUCN Red List of Threatened Species: *Muntiacus vuquangensis. IUCN Red List of Threatened Species* (2015).

122. R. J. Timmins, R. Steinmetz, N. Samba Kumar, M. Anwarul Islam, H. Sagar Baral, IUCN Red List of Threatened Species: *Muntiacus vaginalis. IUCN Red List of Threatened Species* (2015).

123. C. Barrette, Some aspects of the behaviour of muntjacs in Wilpattu National Park. *Mammalia* **41**, 1–34 (1977).

124. J. Chen, X.-B. Deng, Z.-L. Bai, Q. Yang, G.-Q. Chen, Y. Liu, Z.-Q. Liu, Fruit characteristics and *Muntiacus muntijak vaginalis* (Muntjac) visits to individual plants of *Choerospondias axillaris*. *Biotropica* **33**, 718–722 (2001).

125. L. Teng, Z. Liu, Y.-L. Song, Z. Zeng, Forage and bed sites characteristics of Indian muntjac (*Muntiacus muntjak*) in Hainan Island, China. *Ecol. Res.* **19**, 675–681 (2004).

126. B. Lekagul, J. A. McNeely, *Mammals of Thailand* (Association for the Conservation of Wildlife, Bangkok, 1977).

127. R. Steinmetz, D. L. Garshelis, W. Chutipong, N. Seuaturien, Foraging ecology and coexistence of Asiatic black bears and sun bears in a seasonal tropical forest in Southeast Asia. *J. Mammal.* **94**, 1–18 (2013).

128. S. Koike, Long-term trends in food habits of Asiatic black bears in the Misaka Mountains on the Pacific coast of central Japan. *Mamm. Biol.* **75**, 17–28 (2010).

129. M.-H. Hwang, D. L. Garshelis, Y. Wang, Diets of Asiatic Black Bears in Taiwan, with methodological and geographical comparisons. *Ursus* **13**, 111–125 (2002).

130. R. Narita, A. Sugimoto, A. Takayanagi, Animal components in the diet of Japanese black bears *Ursus thibetanus japonicus* in the Kyoto area, Japan. *Wildl. Biol.* **12**, 375–384 (2006).

131. K. Yamazaki, C. Kozakai, S. Koike, H. Morimoto, Y. Goto, K. Furubayashi, Myrmecophagy of Japanese black bears in the grasslands of the Ashio area, Nikko National Park, Japan. *Ursus* **23**, 52–64 (2012).

132. Y. Hashimoto, Seasonal food habits of the Asiatic black bear (*Ursus thibetanus*) in the Chichibu Mountains, Japan. *Mamm. Study* **27**, 65–72 (2002).

133. C. Williams, S. K. Tiwari, V. R. Goswami, S. de Silva, A. Kumar, N. Baskaran, K. Yoganand, V. Menon, *IUCN Red List of Threatened Species: Elephas maximus*, (IUCN Red List of Threatened Species, 2019).

134. T. Gray, J. Borah, C. N. Z. Coudrat, Y. Ghimirey, A. Giordano, E. Greenspan, W. Petersen, S. Rostro-Garcia, M. Shariff, W. Wai-Ming, *IUCN Red List of Threatened Species: Neofelis nebulosa*, (IUCN Red List of Threatened Species, 2020).

135. J. F. Kamler, N. Songsasen, K. Jenks, A. Srivathsa, L. Sheng, (2015).

136. B. V. Valkenburgh, Iterative evolution of hypercarnivory in canids (Mammalia:Carnivora): Evolutionary interactions among sympatric predators. *Paleobiology* **17**, 340–362 (1991).

137. K. U. Karanth, M. E. Sunquist, Prey selection by tiger, leopard and dhole in tropical forests. *J. Anim. Ecol.* **64**, 439–450 (1995).

138. A. P. Andheria, K. U. Karanth, N. S. Kumar, Diet and prey profiles of three sympatric large carnivores in Bandipur Tiger Reserve, India. *J. Zool.* **273**, 169–175 (2007).

139. T. Ramesh, R. Kalle, K. Sankar, Q. Qureshi, Dietary partitioning in sympatric large carnivores in a tropical forest of Western Ghats, India. *Mamm. Study* **37**, 313–321 (2012).

140. K. M. Selvan, G. G. Veeraswami, S. Lyngdoh, B. Habib, S. A. Hussain, Prey selection and food habits of three sympatric large carnivores in a tropical lowland forest of the Eastern Himalayan Biodiversity Hotspot. *Mamm. Biol.* **78**, 296–303 (2013).

141. K. M. Selvan, G. G. Veeraswami, S. A. Hussain, Dietary preference of the Asiatic wild dog (*Cuon alpinus*). *Mamm. Biol.* **78**, 486–489 (2013).

142. J. F. Kamler, A. Johnson, C. Vongkhamheng, A. Bousa, The diet, prey selection, and activity of dholes (*Cuon alpinus*) in northern Laos. *J. Mammal.* **93**, 627–633 (2012).

143. A. J. T. Johnsingh, Reproductive and social behaviour of the Dhole, *Cuon alpinus* (Canidae). *J. Zool.* **198**, 443–463 (1982).

144. A. B. Venkataraman, Do dholes (*Cuon alpinus*) live in packs in response to competition with or predation by large cats? *Curr. Sci.* **69**, 934–936 (1995).

145. R. Sukumar, *The Asian Elephant: Ecology and Management* (Cambridge Univ. Press, ed. 2nd, 1992).

146. N. Baskaran, N. Baskaran, M. Balasubramanian, S. Swaminathan, A. A. Desai, Feeding ecology of the Asian Elephant *Elephas maximus Linnaeus* in the Nilgiri Biosphere Reserve, Southern India. *J. Bombay Nat. Hist. Soc.* **107**, 3–13 (2010).

147. A. Campos-Arceiz, S. Blake, Megagardeners of the forest—The role of elephants in seed dispersal. *Acta Oecol.* **37**, 542–553 (2011).

148. M. English, G. Gillespie, M. Ancrenaz, S. Ismail, B. Goossens, S. Nathan, W. Linklater, Plant selection and avoidance by the Bornean elephant (*Elephas maximus borneensis*) in tropical forest: Does plant recovery rate after herbivory influence food choices? *J. Trop. Ecol.* **30**, 371–379 (2014).

149. C. P. Groves, F. Kurt, Dicerorhinus sumatrensis. *Mamm. Species* **21**, 1–6 (1972).

150. M. Hullot, P.-O. Antoine, M. Ballatore, G. Merceron, Dental microwear textures and dietary preferences of extant rhinoceroses (Perissodactyla, Mammalia). *Mamm Res* **64**, 397–409 (2019).

151. R. Schenkel, L. Schenkel-Hulliger, The Javen rhinoceros (*Rh. sondaicus Desm.*) in Udjung Kulon Nature Reserve. Its ecology and behavior. Field study 1967 and 1968. *Acta Trop.* **26**, 97–135 (1969).

152. R. M. Nowak, *Walker's Mammals of the World* (Johns Hopkins Univ. Press, ed. 6th, 1999), vols. 1–2.

153. J. Hu, F. Wei, "Comparative ecology of giant pandas in the five mountain ranges of their distribution in China" in *Giant Pandas: Biology and Conservation*, D. Lindburg, K. Baragona, Eds. (Univ. of California Press, 2004), pp. 137–148, 10.1525/california/9780520238671.003.0015.

154. Z. Zhang, R. R. Swaisgood, S. Zhang, L. A. Nordstrom, H. Wang, X. Gu, J. Hu, F. Wei, Old-growth forest is what giant pandas really need. *Biol. Lett.* **7**, 403–406 (2011).

155. S. Wang, D. Epron, K. Kobayashi, S. Takanashi, M. Dannoura, Sources of carbon supporting the fast growth of developing immature moso bamboo (*Phyllostachys edulis*) culms: Inference from carbon isotopes and anatomy. *AoB Plants* **15**, plad046 (2023).

156. K. Nowell, P. Jackson, *Wild Cats: Status Survey and Conservation Action Plan* (IUCN, Gland, 1996). *IUCN/SSC Action Plans for the Conservation of Biological Diversity.*

157. M. W. Hayward, P. Henschel, J. O'Brien, M. Hofmeyr, G. Balme, G. I. H. Kerley, Prey preferences of the leopard (*Panthera pardus*). *J. Zool.* **270**, 298–313 (2006).

158. J. Goodrich, H. Wibisono, D. Miquelle, A. J. Lynam, E. Sanderson, S. Chapman, T. N. E. Gray, P. Chanchani, A. Haridar, *IUCN Red List of Threatened Species: Panthera tigris*, (IUCN Red List of Threatened Species, 2021).

159. M. Sunquist, F. Sunquist, *Wild Cats of the World* (Univ. of Chicago Press, 2002), 10.7208/chicago/9780226518237.001.0001.

160. C. Traeholt, W. Novarino, S. bin Saaban, N. M. Shwe, A. Lynam, Z. Zainuddin, B. Simpson, S. bin Mohd, *IUCN Red List of Threatened Species: Tapirus indicus. IUCN Red List of Threatened Species* (2014).

161. B. K. Simpson, M. N. Shukor, D. Magintan, Food selection of the Malayan tapir (Tapirus indicus) under semi-wild conditions. *AIP Conf. Proc.* **1571**, 317–324 (2013).
